# Wafer‐Scale Fabrication of Edge‐Contacted Nanosheet Transistors via Alloying‐Mediated Phase Engineering

**DOI:** 10.1002/smsc.202500320

**Published:** 2025-10-30

**Authors:** Sora Jang, Seunguk Song, Juwon Han, Aram Yoon, Jaewon Wang, Hyeonwoo Lee, Young Ho Jin, Yeoseon Sim, Zonghoon Lee, Changwook Jeong, Soon‐Yong Kwon

**Affiliations:** ^1^ Department of Materials Science and Engineering & Graduate School of Semiconductor Materials and Devices Engineering Ulsan National Institute of Science and Technology (UNIST) Ulsan 44919 Republic of Korea; ^2^ Department of Energy Science Sungkyunkwan University (SKKU) Suwon 16419 Republic of Korea; ^3^ Center for 2D Quantum Heterostructures (2DQH) Institute for Basic Science (IBS) Suwon 16419 Republic of Korea; ^4^ Center for Multidimensional Carbon Materials (CMCM) Institute for Basic Science (IBS) Ulsan 44919 Republic of Korea

**Keywords:** contact length scaling, contact resistance, edge contact, selective‐area growth, semimetal electrode, transition metal dichalcogenide

## Abstract

Edge contacts offer significant potential for scaling down 2D transistors due to their minimal contact resistance and reduced contact length. However, their intricate fabrication complicates reproducible large‐scale production and evaluation of electrical properties, particularly for p‐type channels. Here, the wafer‐scale production of p‐type nanosheet transistors with pure edge contacts by leveraging the alloying‐mediated phase engineering of 2D MoTe_2_ is demonstrated. The relative 1T’‐phase stability of W_*x*_Mo_1−*x*_Te_2_ facilitates the one‐pot growth of lateral polymorphic junctions by combining the 2H‐single‐crystalline MoTe_2_ channels with W_*x*_Mo_1−*x*_Te_2_ edge contacts. These edge‐contact transistors exhibit improved carrier transfer, which is attributed to the impurity‐free contact interface and suppressed metal‐induced gap states. Consequently, their electrical performance is both exceptional and reproducible, compared with that of transistors fabricated using two‐step metallization. Furthermore, irrespective of contact length scaling (8–15 nm), the contact resistivity remains consistently low (≈5.9 × 10^−7^ Ω cm^2^) owing to edge‐confined transport, providing a promising ultra‐scaled contact scheme for Ångström‐node 2D integrated circuits.

## Graphical abstract

This article presents a growth strategy for fabricating edge‐contact transistors composed of polymorphic MoTe_2_. Simultaneous assembly of electronic components facilitates their mass production and improved carrier transport at the metal‐semiconductor junction, attributed to an impurity‐free contact interface and the suppression of metal‐induced gap states. Additionally, the edge‐contact scheme offers advantages in immunity to contact length scaling.

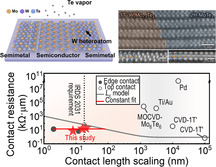

## 1 Introduction

The advancement of contemporary microelectronics is expected to be driven by field‐effect transistors (FETs) with 2D multisheet channels wrapped in gate‐dielectric stacks, which are suitable for gate‐all‐around or complementary FETs.^[^ ^1^, ^2^, ^3^ ^]^ The 2D FETs also offer high‐density integrability and scalability, making them promising for low‐power, low‐latency computing architectures through a 3D tier‐by‐tier integration of logic, memory, and sensor systems.^[^ ^3^, ^4^ ^]^ Nevertheless, these advancements necessitate the development of new technical processes and integration schemes to assemble electrical components while maintaining minimal series resistance. Specifically, the nonplanar structures of 2D multisheet FETs require the metallization of the 1D edge of the 2D channel rather than conventional top surface contacts, which have been extensively studied for 2D FETs with a planar structure.^[^ ^5^, ^6^, ^7^ ^]^ In addition, their contacts should satisfy scaling target of ≈18 nm by 2031,^[^ ^8^ ^]^ which is challenging for top contacts because of their larger physical contact lengths (*L*_c_). Hence, the edge contact utilized in 2D FETs serves as an alternative because its edge‐to‐edge covalent bonds are physically confined to atomic thickness, allowing *L*_c_ to reach approximately zero. In theory, their clean and sharp 1D contact interface offers an additional advantage over the top contact, providing a lower contact resistance (*R*_c_). This is attributed to the strong orbital overlap between the metal and semiconductor and the absence of van der Waals (vdW) gaps at the junction (≈3–4 Å) that could introduce a tunneling barrier for top‐contact geometry.^[^ ^9^, ^10^ ^]^

However, the complex fabrication process often impedes scalable production and the systematic investigation of edge contacts. Creating a pure edge contact with 3D metal requires additional protection layers, such as hBN and AlO_*x*_, to prevent metallization onto the basal plane of the 2D channel.^[^ ^11^, ^12^, ^13^ ^]^ The etch‐and‐deposition process exposes the edges of 2D planes to air, complicating the production of clean 3D edge contacts without oxidation.^[^ ^12^, ^14^, ^15^ ^]^ This two‐step metallization is particularly difficult for p‐type FETs because (i) 2D transition metal dichalcogenides (TMDs) that have shallow valence‐band maxima (e.g., WSe_2_ and MoTe_2_) are not stable in air,^[^ ^12^, ^14^, ^15^ ^]^ and (ii) high‐work‐function metals (e.g., Au and Pd) adhere poorly in edge‐contact configurations,^[^ ^16^ ^]^ as opposed to the extensively studied n‐type FETs. The lack of proper edge contact in p‐type FET hinders the high performance of 2D complementary FETs in logic gate applications.

2H‐phase vdW MoTe_2_ is considered one of the most promising p‐type channel materials among group‐VI 2D TMDs,^[^ ^17^ ^]^ which is attributed to its relatively high chalcogen vacancy formation energy^[^ ^18^ ^]^ and its low valence band maximum (*E*_VBM_ = 4.9–5.1 eV). In addition, the small formation energy difference between the polymorphic phases of MoTe_2_ (≈35 meV)^[^ ^19^ ^]^ enables approaches to realize edge‐contact 2D FETs through polymorphic phase control (between the semi‐metallic 1T’ and semiconducting 2H phases of TMDs)^[^ ^20^, ^21^ ^]^ or direct growth of in‐plane 2D‐2D metal‐semiconductor junction (MSJ).^[^ ^22^, ^23^, ^24^, ^25^ ^]^ However, most relevant strategies have encountered limitations in achieving scalable, impurity‐free, wafer‐scale production owing to narrow windows for phase transition or growth via chemical vapor deposition (CVD).^[^ ^22^, ^24^, ^25^ ^]^ In addition, hybrid contacts with top‐contact geometries are often mistakenly referred to as edge contacts, and their implementation in 2D flakes raises significant concerns regarding practical processability. To address these challenges, it is crucial to develop large‐area, single‐crystalline 2D nanosheet FETs with high‐quality edge contacts, applicable to scaled contact lengths of ≈18 nm for sub‐1 nm technology nodes in future microelectronics.^[^ ^8^ ^]^

In this study, we present the wafer‐scale, high‐quality production of MoTe_2_ FET arrays incorporating synthetic edge contacts composed of 1T’‐phase W_*x*_Mo_1−*x*_Te_2_ semimetal and a single‐crystalline 2H‐MoTe_2_ channel. The phase stability of the 1T’ polymorph of alloyed W_*x*_Mo_1−*x*_Te_2_ (W:MoTe_2_) during CVD provides substantial flexibility for incorporating the semi‐metallic element into lateral 2D circuits because spatially homogeneous MSJ patterns are achieved using conventional lithography techniques. The edge‐contact interface features an ultraclean and sharp atomic connection between the 2H and 1T’ structures of W_*x*_Mo_1−*x*_Te_2_, which enhances carrier transport. For instance, the edge‐contact FETs exhibit a high on‐to‐off current ratio (>8 × 10^4^) and high hole mobility (>10 cm^2^ V^−1^ s^−1^), which are more than 10 times higher than those of two‐step metalized 2H‐MoTe_2_ FETs, while assuring lower device‐to‐device variation (*C*_V_ < 0.072). The atomically thin contact geometry, combined with the low density of states (DOS) of the semimetal, synergistically minimizes metal‐induced gap states (MIGS), ensuring efficient hole transport and significantly reducing contact resistivity (*ρ*_c_ ≈ 5.9 × 10^−7^ Ω cm^2^) compared with that of top‐contact FETs (≈5.33 × 10^−4^ Ω cm^2^), regardless of contact length scaling to less than 10 nm. Therefore, this approach promises to facilitate the manufacturing of miniaturized 2D nanosheet FETs.

## 2 Results and Discussion

### 2.1 Simultaneous Synthesis of Polymorphic MoTe_2_ Heterojunctions

**Figure 1:**
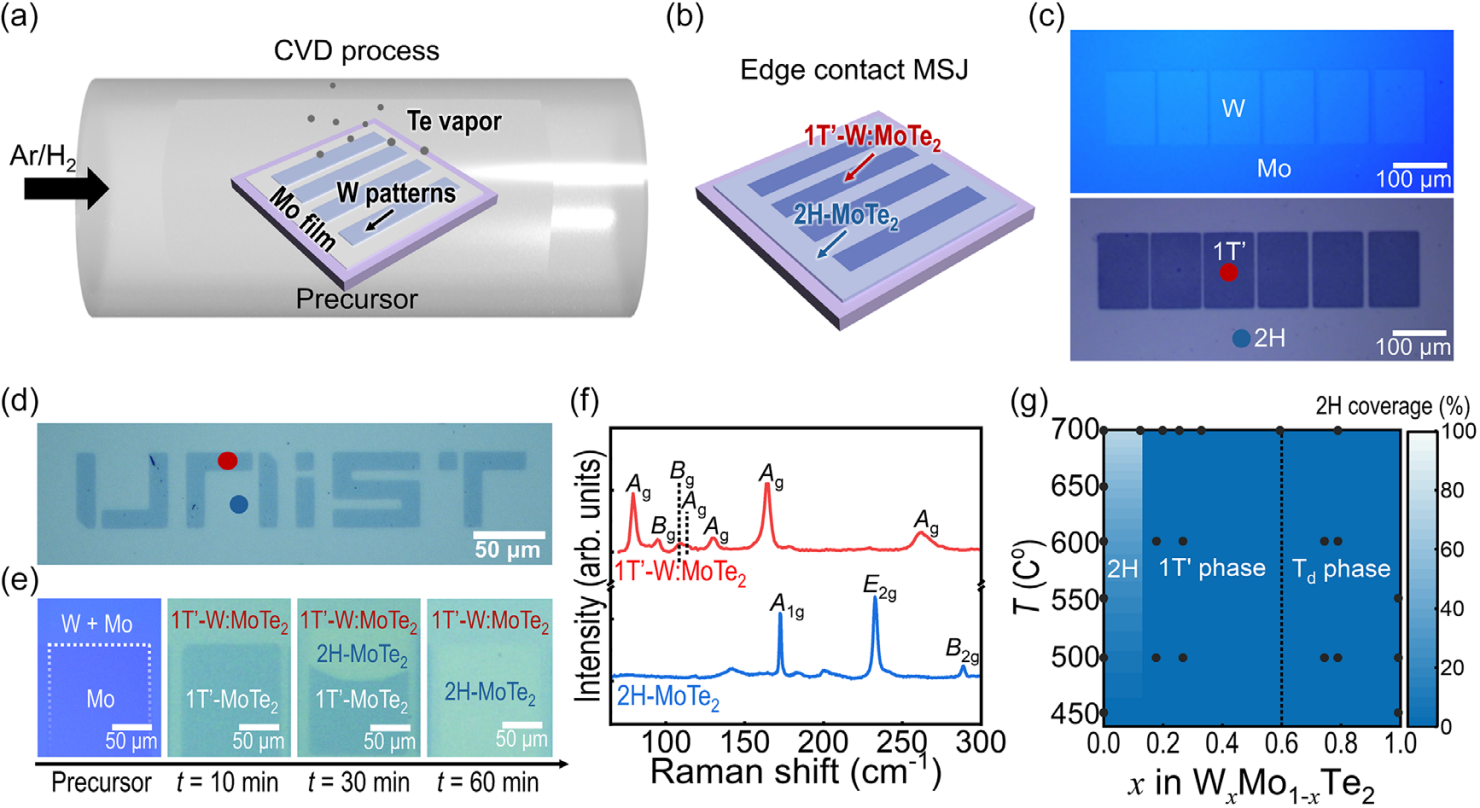
One‐pot synthesis of MoTe_2_ edge‐contact MSJs. a,b) Schematic illustrating position‐controlled synthesis for polymorphic phases achieved through prepatterned transition‐metal precursor films. c) Corresponding OM images for precursor (top) and synthesized W:MoTe_2_‐MoTe_2_ heterostructure (bottom). d) OM image showcasing the creation of the “UNIST” letters and logo constructed from 1T’‐W:MoTe_2_, with the other areas comprising 2H‐MoTe_2_. e) OM images displaying W/Mo transition metal precursor films, synthesized MoTe_2_, and W:MoTe_2_ at a growth temperature of 700 °C, as dependent on growth time (*t*). f) Representative Raman spectra of 2H‐MoTe_2_ (blue) and 1 T’‐W:MoTe_2_ (red) captured at the marks in (c) and (d). The representative Raman peaks of *E*_2g_ (233 cm^−1^) in 2H‐MoTe_2_ and *A*_g_ (163 and 260 cm^−1^) in 1T’‐W:MoTe_2_ demonstrate the achievement of selective phase control. g) Phase diagram of W_*x*_Mo_1−*x*_Te_2_ as a function of W compositions (*x*) and growth temperatures (*T*). The contour plot is generated through linear interpolation of data points (black dots).

To produce high‐quality edge contacts, we propose a growth strategy for lateral MSJs. Previous attempts to achieve edge‐contact MSJs have encountered several challenges owing to the two‐step metallization process,^[^ ^20^, ^21^, ^26^ ^]^ which involves complex lithography processes or laser treatments in the presence of air. This process leads to the formation of chalcogen vacancies and oxidation‐related defects in the 2D lattice. Furthermore, a CVD‐based process for lateral 1T’‐2H MSJ heterostructures often results in the formation of an undesired vertical overlap between the 1T’ and 2H structures or faces difficulties in spatially controlling the phase^[^ ^22^, ^24^, ^25^ ^]^ (Figure S1, Supporting Information). Alternatively, we suggest a simultaneous selective‐area growth strategy for both (semi‐) metallic (1T’‐W_*x*_Mo_1−*x*_Te_2_) and semiconducting (2H‐MoTe_2_) 2D electronic components while ensuring a pure edge connection (**Figure** **1**a–c).

The growth process commenced with the global deposition of a Mo precursor thin film onto a SiO_2_/Si substrate, followed by the deposition of arrays of W thin film patterns using conventional photolithography. These patterned W/Mo and Mo precursor films were then stacked face‐to‐face using Ni_*x*_Te_*y*_ eutectic alloy, which is a Te source for tellurization at a temperature of ≈700 °C (Figure 1a). The choice of Ni_*x*_Te_*y*_ as the Te vapor precursor ensured a Te‐rich environment in the gas‐confined structure for the growth of 2H‐phase MoTe_2_ with a highly stoichiometric nature.^[^ ^27^ ^]^ During tellurization, the Mo region converted to MoTe_2_. In contrast, because of the presence of W heteroatoms, the W/Mo layers transformed into W‐substituted MoTe_2_, whose thickness was comparable to that of pristine 2H‐MoTe_2_ (Figure 1b and S2, Supporting Information). The different phases in edge‐contact MSJ heterostructure are clearly distinguished by distinct contrasts of the optical microscopy (OM) image (Figure 1c). This area‐selective phase control can be applied regardless of the pattern shape. As an illustrative example, we have successfully prepared “UNIST” letters comprising both 1T’ and 2H phases (Figure 1d).

The achievement of 2H‐MoTe_2_ from the Mo precursor is attributed to the phase transition that occurs during the abnormal grain growth of the 2H single‐crystal domain from 1T’ structure.^[^ ^28^ ^]^ In contrast, no phase transition from 1T’ to 2H is observed in the W/Mo‐deposited region. The effectiveness of this phase control technique, depending on the growth time (*t*), is further corroborated by the findings presented in Figure 1e. As *t* increased from 10 to 30 min, circular 2H‐phase regions emerged and expanded in the Mo‐deposited areas, while the W/Mo‐deposited regions existed in the 1T’ phase, regardless of *t*. Over prolonged growth periods (e.g., *t* ≥ 60 min), the entire Mo‐precursor region eventually transitioned to the 2H‐phase as a result of abnormal grain growth,^[^ ^28^ ^]^ while the W/Mo‐deposited region retained the 1T’ phase. We also found that the initial 2H nucleation occurs only when the separation between W:MoTe_2_ regions is sufficiently large (>11 μm), enabling site‐selective growth of single‐crystalline 2H structures (Figure S3–S5 and Table S1, Supporting Information).

Each phase was discerned through confocal Raman spectroscopy (Figure 1f). The characteristic Raman peaks of the 1T’ phase (e.g., *A*_g_ of 163 and 260 cm^−1^) are observed in both red points in Figure 1c,d. Meanwhile, the regions where only Mo was deposited in each image (blue points) exhibit signals corresponding to 2H‐phase MoTe_2_ (e.g., *A*_1g_ of 173 cm^−1^, *E*_2g_ of 233 cm^−1^, and *B*_2g_ of 289 cm^−1^). Notably, the Raman spectrum of 1T’‐W:MoTe_2_ closely resembles those of 1T’‐MoTe_2_ and T_d_‐WTe_2_.^[^ ^29^ ^]^ The different phases in the lateral MSJ were also identified through Raman mapping, electron backscatter diffraction (EBSD) characterizations, and surface potential measurements obtained by Kelvin probe force microscopy (KPFM) (Figure S6 and S7, Supporting Information). These results show a sharp interface between two polymorphs, validating the phase‐controlled growth of MoTe_2_ in a spatial way.

Figure 1g presents a polymorphic phase diagram based on the growth results, illustrating the dependence of the coverage of the 2H phase (i.e., % volume ratio of 2H/1T’) on the growth temperature (*T*) and composition (*x*) of the ternary W_*x*_Mo_1−*x*_Te_2_ crystal. The composition (*x*) was adjusted by varying the sputtering power for W deposition while maintaining a consistent deposition time and Mo thickness (Figure S8, Supporting Information). An *x* higher than ≈0.13 allows for the stable achievement of the 1T’ or T_d_ phase over the 2H structure in W_*x*_Mo_1−*x*_Te_2_ (Figure S9, Supporting Information), similar to previous report (*x* > 0.1).^[^ ^29^ ^]^ The phase‐selective synthesis of W:MoTe_2_ is ascribed to the thermodynamic stability of its composition‐dependent phase, as suggested by previous density functional theory (DFT) calculation.^[^ ^30^ ^]^ The free energy of 1T’‐phase formation in MoTe_2_ decreases with increasing W composition (*x*) in the MoTe_2_ matrix, becoming even lower than that of the 2H phase when *x* > 0.13. The increase in *x* further induces a phase transition from 1T’ to T_d_, as discussed later.

### 2.2 Structural and Electrical Characterizations of W_*x*_Mo_1−*x*_Te_2_

**Figure 2:**
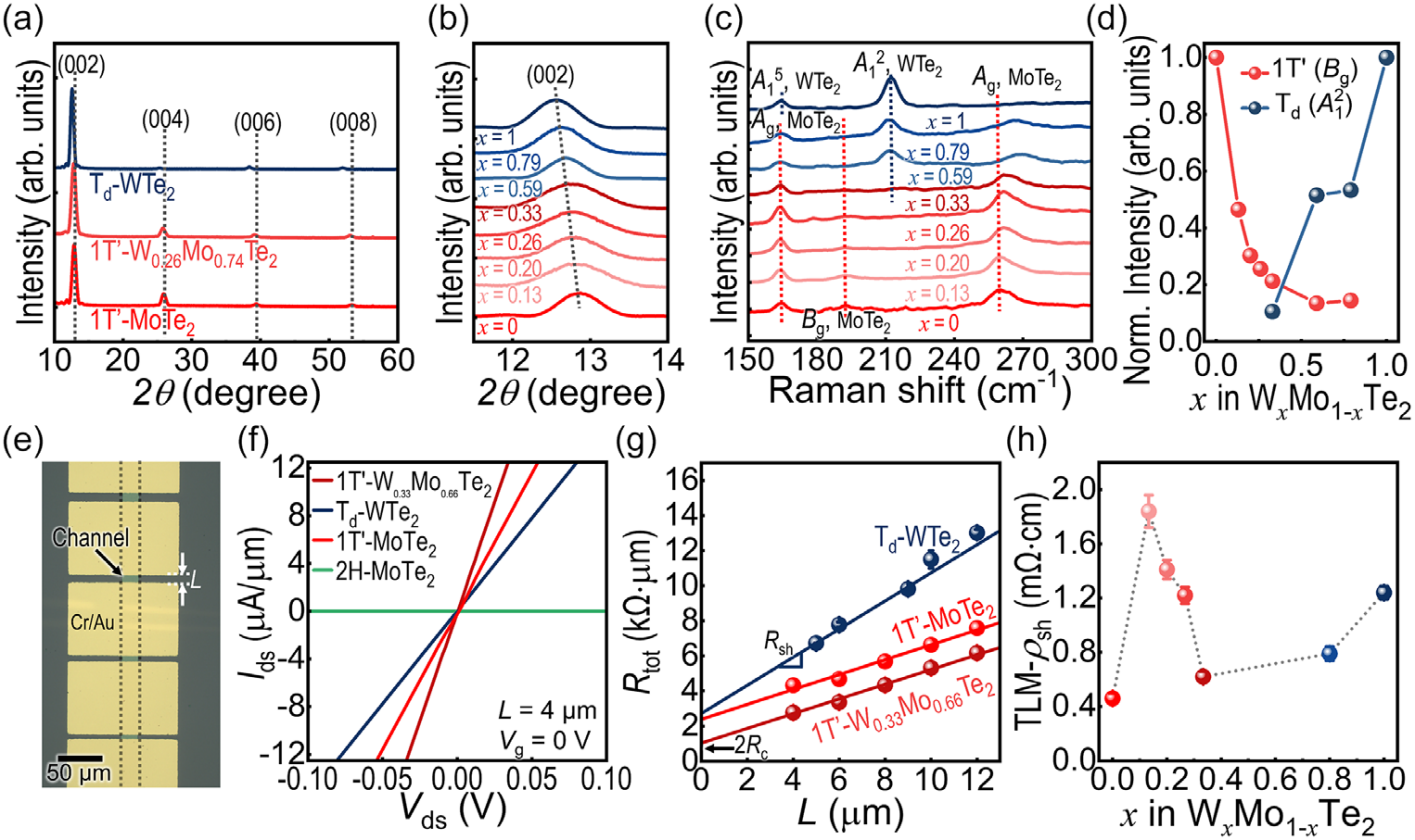
Structural and electrical characteristics of W_*x*_Mo_1−***x***_Te_2_ thin films. a) Representative XRD spectra of W_*x*_Mo_1−*x*_Te_2_ thin films. All the W_*x*_Mo_1−*x*_Te_2_ are synthesized under the same condition at *T* = 700 °C, *t* = 2 h, while MoTe_2_ and WTe_2_ are synthesized at *T* = 500 °C, *t* = 10, 30 min. b) XRD spectra of W_*x*_Mo_1−*x*_Te_2_ in a narrow range for observing (002) peak shift depending on W composition. W composition is extracted by approximating peak values to Vegard's law. c) Raman spectra of W_*x*_Mo_1−*x*_Te_2_ with different W concentrations of *x* from 0 to 1. d) Normalized intensities of characteristic peaks of MoTe_2_ (*B*_g_ mode at 192 cm^−2^) and WTe_2_ (*A*_1_^2^ mode at 212 cm^−2^) versus *x* plot, which are identified from (c). e–h) Electrical properties of W_*x*_Mo_1−*x*_Te_2_ thin films. e) Representative OM image of TLM patterned metallic W_*x*_Mo_1−*x*_Te_2_ devices. f) Representative output characteristics (drain voltage (*V*_ds_) vs. drain current (*I*_ds_)) of W_*x*_Mo_1−*x*_Te_2_ devices. No gate bias is applied. (g) TLM plots represented by linear plots of total resistance (*R*_tot_) versus channel length (*L*) of Cr/Au contacted W_*x*_Mo_1−*x*_Te_2_ FETs. h) Plot showing sheet resistivity (*ρ*_sh_) of W_*x*_Mo_1−*x*_Te_2_ thin films dependent on W composition of *x*.

The modulation of W incorporation in W:MoTe_2_ provides additional flexibility for tailoring the structural and electrical properties of the 2D (semi‐) metallic layers. To validate this, we grew and characterized large‐scale (≈1 × 1 cm^2^) thin films of 1T’‐ or T_d_‐phase W:MoTe_2_ (Figure S9, Supporting Information). X‐ray diffraction (XRD) measurements were conducted to identify the structural differences in the W:MoTe_2_ crystals over the range of *x* from 0 to 1. The XRD patterns revealed that all the crystals were aligned along the (002*l*) plane direction (**Figure** **2**a), indicating *c*‐plane textured thin films.^[^ ^31^ ^]^ No 2H phase‐ or oxide‐related peaks were observed in the XRD patterns. The (002*l*) peak positions shifted to smaller angles as the W content increased (Figure 2b and S10, Supporting Information), which was attributed to the larger lattice constant. For example, the lattice constant *c* in the unit cell of T_d_‐WTe_2_ is ≈14.07 Å, whereas it is smaller for 1T’‐MoTe_2_ (≈13.86 Å).^[^ ^32^ ^]^ Furthermore, based on the (002) peaks of the films, the W composition was determined using Vegard's law,^[^ ^33^ ^]^ as shown in Figure 2b. The effective change in the (002) peak positions without peak separation implies successful composition modulation and compositional homogeneity across the thin films. This W atomic substitution in 1T’‐MoTe_2_ is further evidenced by X‐ray photoelectron spectroscopy (XPS) and ultraviolet photoelectron spectroscopy (UPS) analyses (Figure S11 and S12, Supporting Information).

Raman spectroscopy also reflects the structural transition in W_*x*_Mo_1−*x*_Te_2_ dependent on *x*. Crystals with *x* values below 0.33 display Raman vibrational modes resembling those of 1T’‐MoTe_2_ (Figure 2c), except for the emergence of a peak (≈178 cm^−1^) activated by the loss of translation symmetry in the 1T’ lattice^[^ ^29^ ^]^ (Figures S13a,b, Supporting Information). In addition, a slight blue shift was observed in the *A*_g_ signal (260.0–262.3 cm^−1^; Figure S13c, Supporting Information), indicating a change in the out‐of‐plane vibration.^[^ ^34^ ^]^ We also found that when the W composition surpassed *x* of ≈0.5, the *A*_1_^2^ mode (≈212 cm^−1^) of T_d_‐WTe_2_ emerged, as the structural features of the T_d_ phase started to dominate (Figure 2d and S14, Supporting Information). In Figure 2d, we present the relative intensities of *B*_g_ (at ≈192 cm^−1^ for 1T’‐MoTe_2_, red) and *A*_1_^2^ (at ≈212 cm^−1^ for T_d_‐WTe_2_; blue) peaks based on *x* compositions. This plot suggests a gradual 1T’ to T_d_ phase transition with increasing W composition. The *x* values of 0.33–0.59 are identified as the critical compositions where the Raman characteristics expressed by each crystal structure become distinct.

The compositional and structural changes in W:MoTe_2_ significantly influence the electrical properties of the 2D thin films. Two‐terminal devices with W_*x*_Mo_1−*x*_Te_2_ channels and Cr/Au contact electrodes were fabricated and characterized (Figure 2e). Figure 2f displays representative output curves of W_*x*_Mo_1−*x*_Te_2_ (0 ≤ *x* ≤ 1) with different crystal structures: 2H, 1T’ and T_d_. The linear dependence of the drain‐source current (*I*_ds_) on the applied voltage (*V*_ds_) indicates the Ohmic‐like behavior of metallic 1T’‐ or T_d_‐W_*x*_Mo_1−*x*_Te_2_. Additionally, the *I*_ds_ values for 1T’‐ or T_d_‐W_*x*_Mo_1−*x*_Te_2_ are nearly four orders of magnitude higher than that of 2H‐MoTe_2_ at a *V*_ds_ of −0.1 V, attributed to its metallic conduction compared with the semiconducting 2H crystal. Nonetheless, relying solely on two‐terminal devices for electrical characterization cannot eliminate the effects of *R*_c_, leading to a potential over‐ or underestimation of the intrinsic electrical properties. Hence, to accurately examine the sheet resistance (*R*_sh_) while excluding the effect of *R*_c_, we used the transfer length method (TLM) for devices with W:MoTe_2_ channels. This method exploits the information of the total resistance (*R*_tot_) of two‐terminal devices that relates to the channel length (*L*), as follows

(1) $$R_{\text{tot}}=R_{\text{ch}}\left(L\right)+2R_{c}=R_{\text{sh}}L+2R_{c}$$

Consequently, the slopes and *y*‐intercepts in the TLM plots (Figure 2g and S15, Supporting Information) provide insight into the *R*_sh_ and 2*R*_c_ values of the W_*x*_Mo_1−*x*_Te_2_ devices, respectively. For instance, the slopes of 1T’‐MoTe_2_ and 1T’‐W_0.33_Mo_0.67_Te_2_ (*R*_sh_ ≈ 424 and 419 Ω sq^−1^, respectively) are nearly identical, indicating analogous electrical conductance regardless of W incorporation. Importantly, data obtained from each two‐terminal device aligned well with linear fits to Equation (1), demonstrating negligible variations in channel conductance from one device to another. This emphasizes the significant advantage of our growth approach for reproducibly obtaining metallic layered thin films. Notably, this advantage remains even when compared with the mechanical exfoliation method,^[^ ^35^ ^]^ which exhibited variations in the resistivity of WTe_2_ (for instance, TLM‐extracted values ranging randomly from ≈0.5 to 1.5 mΩ cm).

Figure 2h presents a summary of sheet resistivities (*ρ*_sh_; *R*_sh_ in the consideration of channel thickness) obtained via TLM for W_*x*_Mo_1−*x*_Te_2_, illustrating variations across different W compositions. The anomalous increase in *ρ*_sh_ at *x* = 0.13 compared with pristine MoTe_2_ may be explained by the disorder resulting from broken symmetry caused by the insertion of the W heteroatoms.^[^ ^29^, ^36^ ^]^ This electrical tendency is consistent with previous reports on ternary ditelluride alloys, indicating an increase in electrical resistivity at a critical point compared with the monotonic decrease associated with heteroatom inclusion.^[^ ^31^, ^37^ ^]^ Conversely, in the T_d_ phase regime (*x* > 0.5), the increase in *ρ*_sh_ values over *x* is ascribed to the dominating effect of the higher *ρ*_sh_ of T_d_‐WTe_2_ (1.24 ± 0.05 mΩ cm) compared with that of 1T’‐MoTe_2_ (0.46 ± 0.04 mΩ cm), potentially influenced by defect‐limited hopping transport in T_d_‐WTe_2_.^[^ ^35^ ^]^ Additionally, considering that the device fabrication process was conducted in an air atmosphere (see the Experimental Section), the formation of defects induced by the process may affect the electrical properties of WTe_2_.^[^ ^35^, ^38^ ^]^ Nevertheless, the electrical conductance of W_*x*_Mo_1−*x*_Te_2_ with *x* of ≈0.3–0.8 remains consistently within the range of ≈0.6–1.2 mΩ cm. Considering that W incorporation enables the selective achievement of metallic 1T’ structure, our approach holds promise as a reproducible production strategy for 2D metallic components in 2D circuits, especially given their work function tunability (from 4.44 to 4.51 eV depending on the composition; Figure S12, Supporting information).

### 2.3 Atomic Characterizations of Edge‐Contact Interface

**Figure 3:**
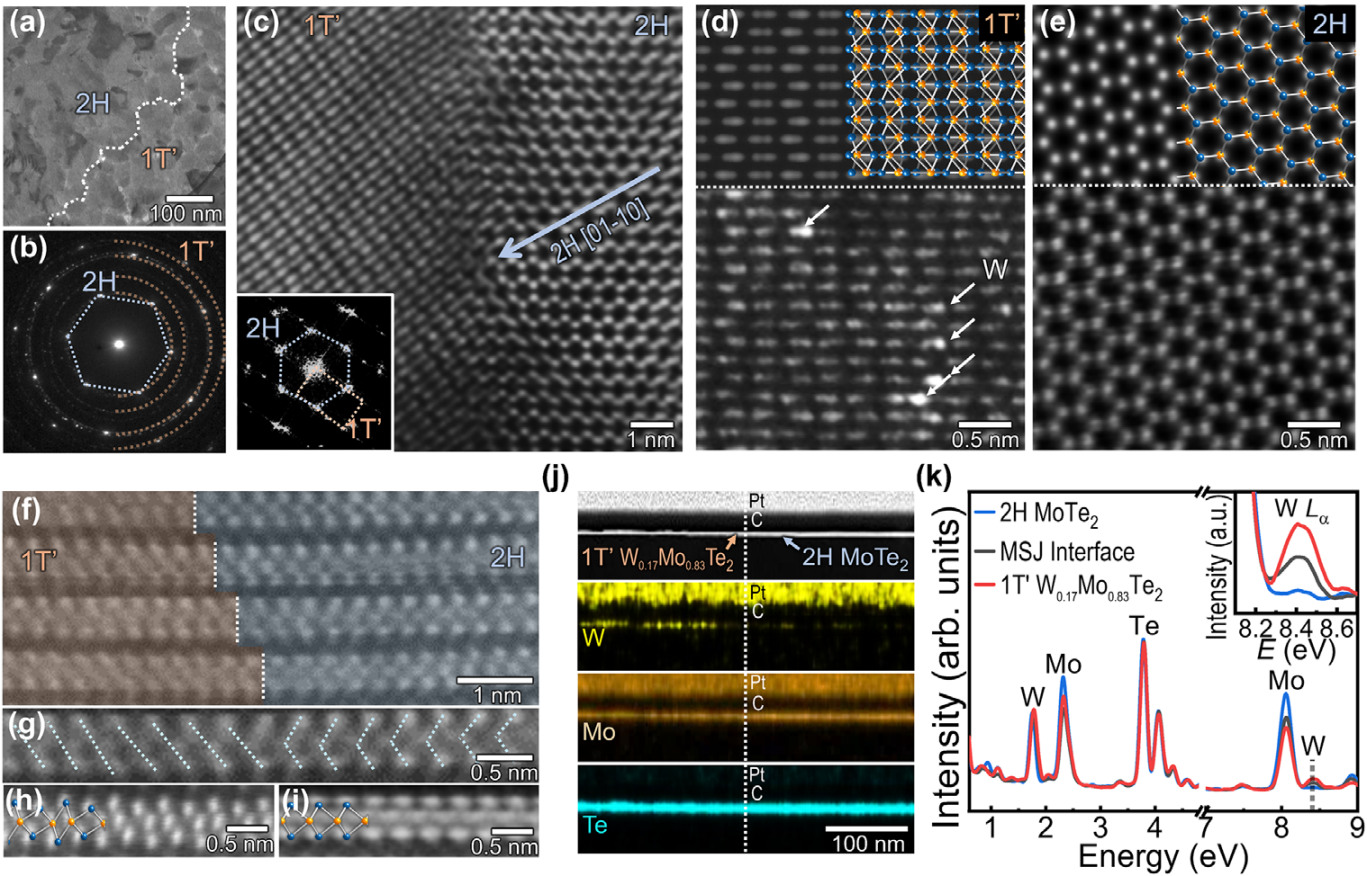
Seamlessly contacted MSJ interface achieved through conformal phase engineering. a) Bright‐field STEM image of the heterojunction of 2H‐MoTe_2_ and 1T’‐W_0.17_Mo_0.83_Te_2_ transferred on the TEM grid. The dashed white lines indicate the grain boundary between 2H and 1T’ structures. b) Selective area electron diffraction (SAED) pattern obtained at the interface of 2H‐MoTe_2_ (blue dotted hexagon) and 1T’‐W_0.17_Mo_0.83_Te_2_ (orange half‐circle). c) HAADF‐STEM image captured at the heterojunction interface. At the junction, an atomically well‐stitched interface is observed. The arrow indicates the [01‐10]_2H_ direction, connected with [200]_1T’_. The inset is the fast‐Fourier‐transform (FFT) pattern, showing the crystal symmetry formed by a diffraction spot of 2H‐MoTe_2_ and 1T’‐W_0.17_Mo_0.83_Te_2_ represented by a blue dotted hexagon and orange dotted rectangle, respectively. d,e) Atomic‐resolution STEM images of two different phases, 1T’ in (d) and 2H in (e). The upper images are simulated HAADF‐STEM images of each phase. The arrows in (d) indicate W heteroatoms, which are brighter than other atoms (Mo, Te). f–i) Cross‐sectional HAADF‐STEM images of the heterojunction observed focused on MoTe_2_ zig‐zag direction. (f,g) Atomic‐resolution STEM image and a zoomed‐in image at the interface. (h,i) High‐magnification images of 1T’ and 2H phases taken far from the junction of each phase in (f). j,k) Cross‐sectional EDS mappings at 1T’/2H interface, containing W, Mo, and Te elements (j), and EDS line profiles scanning from 2H‐MoTe_2_ (blue), through the MSJ interface (gray), to 1T’‐W_0.17_Mo_0.83_Te_2_ (red) (k). The inset shows the W *L*_α_ peak, the intensity of which depends on W composition. The intensities are normalized to Te *L* energy.

To investigate the structure and composition, scanning transmission electron microscopy (STEM) was conducted on the lateral MSJ heterostructure comprising 2H‐MoTe_2_ and 1T’‐W_0.17_Mo_0.83_Te_2_ (**Figure** **3**). Figure 3a,b depict the low‐magnification STEM image and the corresponding selected area electron diffraction (SAED) patterns of the junction, respectively. In SAED patterns, the 2H region is identified as one set of hexagonally arranged diffraction spots, whereas a 1T’ region shows diffraction rings (Figure 3b and S16a,b, Supporting information). Both a single set of sixfold symmetry and sharp Bragg peaks of 2H‐MoTe_2_ with no diffuse scattering imply a single‐crystalline nature,^[^ ^22^ ^]^ in contrast to the polycrystalline 1T’‐phase W‐alloyed MoTe_2_. The atomic‐resolution STEM image shows that the 2H and 1T’ phases of the binary and ternary ditelluride alloys were atomically well‐stitched at the interface without voids (Figure 3c). The corresponding fast‐Fourier‐transform (FFT) pattern exhibits different phases, with a set of monoclinic 1T’ spots and hexagonal 2H spots (inset in Figure 3c). A comprehensive structural analysis of the interface, revealing the atomically connected region between 1T’‐W_0.17_Mo_0.83_Te_2_ and 2H‐MoTe_2_, was conducted using the FFT patterns. Consistent with a previous report,^[^ ^22^ ^]^ the 1T’‐W_0.17_Mo_0.83_Te_2_ is atomically connected to 2H‐MoTe_2_, constructing quasiepitaxial relation of the [01‐10]_2H_//[200]_1T’_ interface. The tilted interface is ascribed to the lattice mismatch between the 1T’ and 2H phases (Figure S16c‐e, Supporting information). This configuration represents the structurally favorable interface between 1T’ and 2H structures.^[^ ^22^, ^28^, ^39^ ^]^

The atomic resolution STEM images of each phase demonstrate the high crystallinity of the monoclinic and hexagonal structures of 1T’ and 2H phases, comparable to those observed in the simulated images (Figure 3d,e). In the high‐angle annular dark field (HAADF)‐STEM image of 1T’ W‐alloyed MoTe_2_ (with a thickness of <3 nm), brighter atomic positions are randomly present (Figure 3d). This is because the HAADF image is derived from the difference in atomic contrast, influenced by the relationship between the intensity and atomic number (≈*Z* ^2^).^[^ ^34^ ^]^ The simulated HAADF intensity difference between Mo (*Z* = 42) and W (*Z* = 74) closely matches the experimental results, confirming the substitution of W into the 1T’‐MoTe_2_ structure (Figure S17, Supporting information).

In addition, we perform cross‐sectional TEM analysis of the MSJ interface to investigate the bonding structure at the edge (Figure 3f–k). The interface, viewed at atomic resolution along the zig‐zag direction of 2H‐MoTe_2_, revealed a clean, edge‐contact interface between the 1T’ and 2H phases (Figure 3f). Note that Figure 3f,g are focused well on the 2H phase but not on the 1T’ phase (compared with those captured separately in Figure 3h,i), because of the slightly tilted in‐plane connection between them (Figure 3c). Apart from the junction interface, the atomic‐resolution STEM images of the 1T’ and 2H phase show that each phase preserved its crystalline structure without any structural deformation (Figure 3h,i).

Elemental analysis is conducted via energy dispersive X‐ray spectroscopy (EDS) (Figure 3j, k, S18, Supporting information). Cross‐sectional TEM‐EDS mappings revealed a uniform distribution of Mo and Te across both the 1T’ and 2H structures, with the absence of W in the 2H‐MoTe_2_ region. Furthermore, the EDS spectra for each phase and their interface demonstrate the dependence of the W compositions on the scanned area (Figure 3k and its inset). The intensity of the W *L*_α_ signal in the region corresponding to the MSJ interface (black) is higher than that in 2H‐MoTe_2_ (blue) but lower than that in 1T’‐W:MoTe_2_ (red; the inset of Figure 3k). The plan‐view EDS mapping images also reveal that the W heteroatoms inserted into 1T’‐MoTe_2_ are uniformly distributed without any segregation (Figure S18, Supporting information). These findings indicate the effectiveness of W incorporation for achieving a seamlessly integrated edge‐contact MSJ interface.

### 2.4 Synthetic Metallization for Edge‐Contact FETs

**Figure 4:**
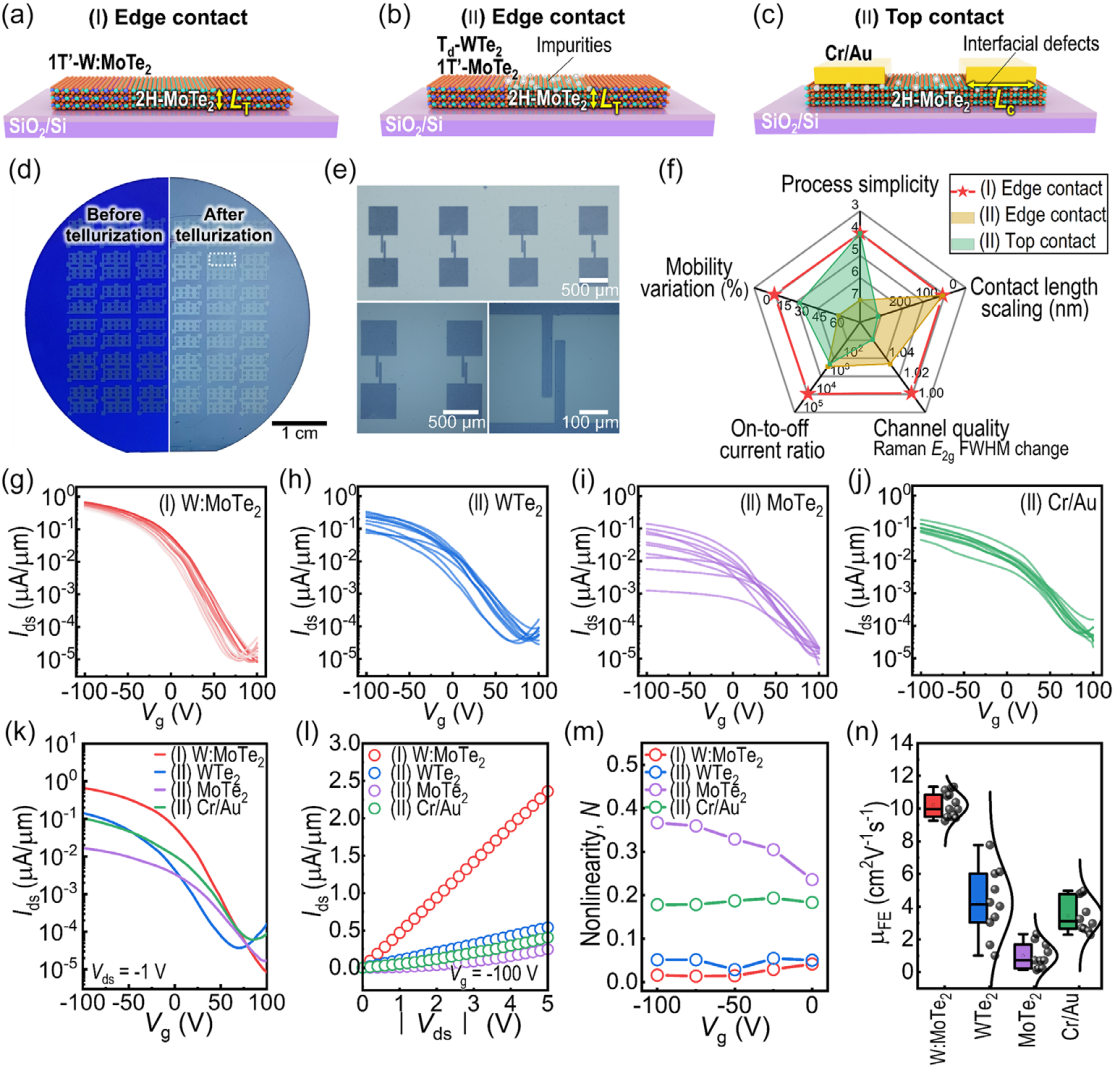
Synthetic metallization to construct high‐quality edge contact. a–c) Schematics illustrating edge‐ and top‐contact MoTe_2_ FETs using different metallization processes. (a) W:MoTe_2_ edge‐contact MSJ FET constructed through a one‐step synthetic method. (b) Edge‐contact MoTe_2_ MSJ FET with 2D electrodes (T_d_‐WTe_2_, 1T’‐MoTe_2_) created using a two‐step synthetic method. (c) Top‐contact MSJ FET with conventional 3D metal (Cr/Au). *L*_T_ and *L*_c_ indicate transfer length of charge carriers and physical contact length, respectively. d) Photographs of a 2‐inch wafer with predeposited patterned precursor films (left) and lateral W:MoTe_2_‐MoTe_2_ edge‐contact MSJ patterns after the tellurization process (right). e) Representative OM images of the heterojunctions at different scales. f) Radar plot illustrating the characteristics of different metallization processes used for fabricating MoTe_2_ FETs in this study (see Table S2 and Figure S19‐S21, Supporting Information for more details). g–j) Transfer curves (gate voltage (*V*_g_) vs. drain current (*I*_ds_)) of FET with (g) one‐step W:MoTe_2_ edge contact, and two‐step (h) WTe_2_, (i) MoTe_2_ edge contact, and (j) Cr/Au top contact (channel length, *L* = 10 or 11 μm and *V*_ds_ = −1 V). k–n) Electrical characteristics of fabricated MoTe_2_ MSJ FETs with different contacts (W:MoTe_2_, WTe_2_, MoTe_2_, and Cr/Au). (k) Representative transfer and (l) output (drain voltage (*V*_ds_) vs. drain current (*I*_ds_)) curves. (m) Nonlinearity (*N*) of the output curves. (n) Statistical plot showing field‐effect mobility (*μ*_FE_) values and their variations, presented as a box plot with gray dots representing experimental data points and a distribution curve (see the Experimental Section for details). Each dataset corresponds to W:MoTe_2_ (10.21 ± 0.73 cm^2^ V^−1^ s^−1^, *n* = 13), T_d_‐WTe_2_ (4.23 ± 1.98 cm^2^ V^−1^ s^−1^, *n* = 10), 1T’‐MoTe_2_ (0.99 ± 0.76 cm^2^ V^−1^ s^−1^, *n* = 10), and Cr/Au metal contacts (3.46 ± 0.99 cm^2^ V^−1^ s^−1^, *n* = 10).

Our one‐step synthetic metallization process (noted as “(I)”) offers significant advantages in producing high‐quality edge‐contact MSJ FETs compared with other metallization processes. The two‐step process (“(II)”) often results in defects such as phase discontinuity, oxide impurities, and vacancies, which our one‐step method minimizes. To demonstrate this, we fabricated MSJ device structures using different methods (**Figure** **4**a–c) and conducted a comparative analysis. Our selective‐area phase control (Figure 4a) eliminates oxidation and misalignment issues, enabling the mass production of high‐quality 2H‐MoTe_2_ FETs with W_*x*_Mo_1−*x*_Te_2_ edge contacts (“(I) W:MoTe_2_”) on 2‐inch wafers (Figure 4d,e). In contrast, the two‐step edge contacts produced using T_d_‐WTe_2_ or 1T’‐MoTe_2_ (“(II) WTe_2_” or “(II) MoTe_2_”) lack spatial phase control and purity, compromising process simplicity and their narrow growth window (Figure 4b and S19, S20, Supporting information). Additionally, the top contact (“(II) Cr/Au”) can severely damage the 2D channel surface beneath the contacts, potentially leading to poor electrical performance and making the scaling of *L*_c_ challenging (Figure 4c). Therefore, in terms of process simplicity, contact length scaling, channel quality, on/off ratio, and mobility variation, our simultaneous synthesis approach for edge‐contact MSJ offers notable advantages (Figure 4f; see Figure S19‐S21 and Table S2, Supporting information for more details).

The gate‐modulated electrical conductance of the MoTe_2_ MSJ FETs was characterized by using a global back gate of SiO_2_ (Figure 4g–n). All the fabricated FETs exhibited p‐type transport regardless of the contact metal, which is attributed to the Fermi level aligned close to the valence band maximum (VBM) of 2H‐MoTe_2,_^[^ ^17^ ^]^ similar to previous reports on CVD‐grown samples.^[^ ^23^, ^24^, ^25^, ^26^, ^28^ ^]^ In the transfer curves that overlapped for more than 10 devices, the 2H‐MoTe_2_ FETs with one‐step W:MoTe_2_ edge contacts demonstrated more reproducible and uniform current transport (Figure 4g) than those measured with two‐step contacts (i.e., (II) T_d_‐WTe_2_, (II) 1T’‐MoTe_2_, and (II) Cr/Au) (Figure 4h–j).

In addition, the one‐step edge contact enables superior electrical performance compared with the other contacts, as shown in the representative transfer and output characteristics (Figure 4k,l). The on‐state current density (*I*_on_) reaches ≈0.67 μA μm^−1^ at a *V*_ds_ of −1 V, with an on‐to‐off current ratio (*I*_on_/*I*_off_) of ≈8 × 10^4^ (on average for 13 devices; Figure 4g and S22, S23a,c, Supporting information). This *I*_on_ and the *I*_on_/*I*_off_ ratio are at least 42 and 80 times greater, respectively, than those of two‐step metalized devices (e.g., *I*_on_ of ≈0.016 μA μm^−1^ and *I*_on_/*I*_off_ ratio of ≈1 × 10^3^ for (II) MoTe_2_ edge contact). The more pronounced improvement in the *I*_on_/*I*_off_ ratio, compared with the increase in *I*_on_, is a result of substantially suppressed *I*_off_ (<10^−5^ μA μm^−1^). This low *I*_off_ is attributed to the high channel quality without unintended doping, which ensures the complete depletion of the 2D channel and low static power in the FET (Figure S23b, Supporting information). Furthermore, a steeper subthreshold swing is observed in the edge‐contact MSJ than in the top‐contact configuration, attributed to the lower interface trap density^[^ ^40^ ^]^—supported by the reduced trap density in the W:MoTe_2_ edge‐contact FET (≈2.02 × 10^13^ cm^−2^ eV^−1^) compared with the Cr/Au top‐contact FET (≈3.88 × 10^13^ cm^−2^ eV^−1^) (Figure S24, Supporting information).

In addition, the minimal nonlinearity (*N* = (d^2^ *I*_ds_/d*V* ^2^_ds_)/2(*I*_ds_/*V*_ds_)) in the output characteristics of the MSJ FET (Figure 4m) indicates its nearly Ohmic contact rather than the existence of a significant barrier height.^[^ ^5^ ^]^ The (I) W:MoTe_2_ edge‐contact FET exhibits nearly zero *N* (average value of 0.023) in the range of *V*_g_ of −100 to 0 V at room temperature (Figure 4m and S25, Supporting information). The *N* values in the (I) edge‐contact FETs were also lower (<0.05) than those in the (II) top‐contact devices (*N* < 0.13) at lower temperatures (180–300 K) (Figure S26, Supporting information). Due to the better contact properties, the field‐effect mobility (*μ*_FE_) of FETs with (I) W:MoTe_2_ edge contacts (10.21 ± 0.73 cm^2^ V^−1^ s^−1^) is higher than that of FETs with (II) T_d_‐WTe_2_ (4.23 ± 1.98 cm^2^ V^−1^ s^−1^), (II) 1T’‐MoTe_2_ (0.99 ± 0.76 cm^2^ V^−1^ s^−1^), and (II) Cr/Au metal contacts (3.46 ± 0.99 cm^2^ V^−1^ s^−1^) (Figure 4n). Moreover, the coefficient of variation (*C*_V_ = variation/average)^[^ ^41^ ^]^ of (I) W:MoTe_2_ edge‐contact FETs is the lowest (≈0.072) among the tested device sets (≈0.28–0.76), which suggests the reproducibility of the edge contact. Together with the 2‐inch wafer‐scale uniformity (Figure S27 and S28, Supporting information), our edge‐contact FETs ensure reproducible and reliable large‐scale integration.

### 2.5 Carrier Transport Depending on Contact Geometry

**Figure 5:**
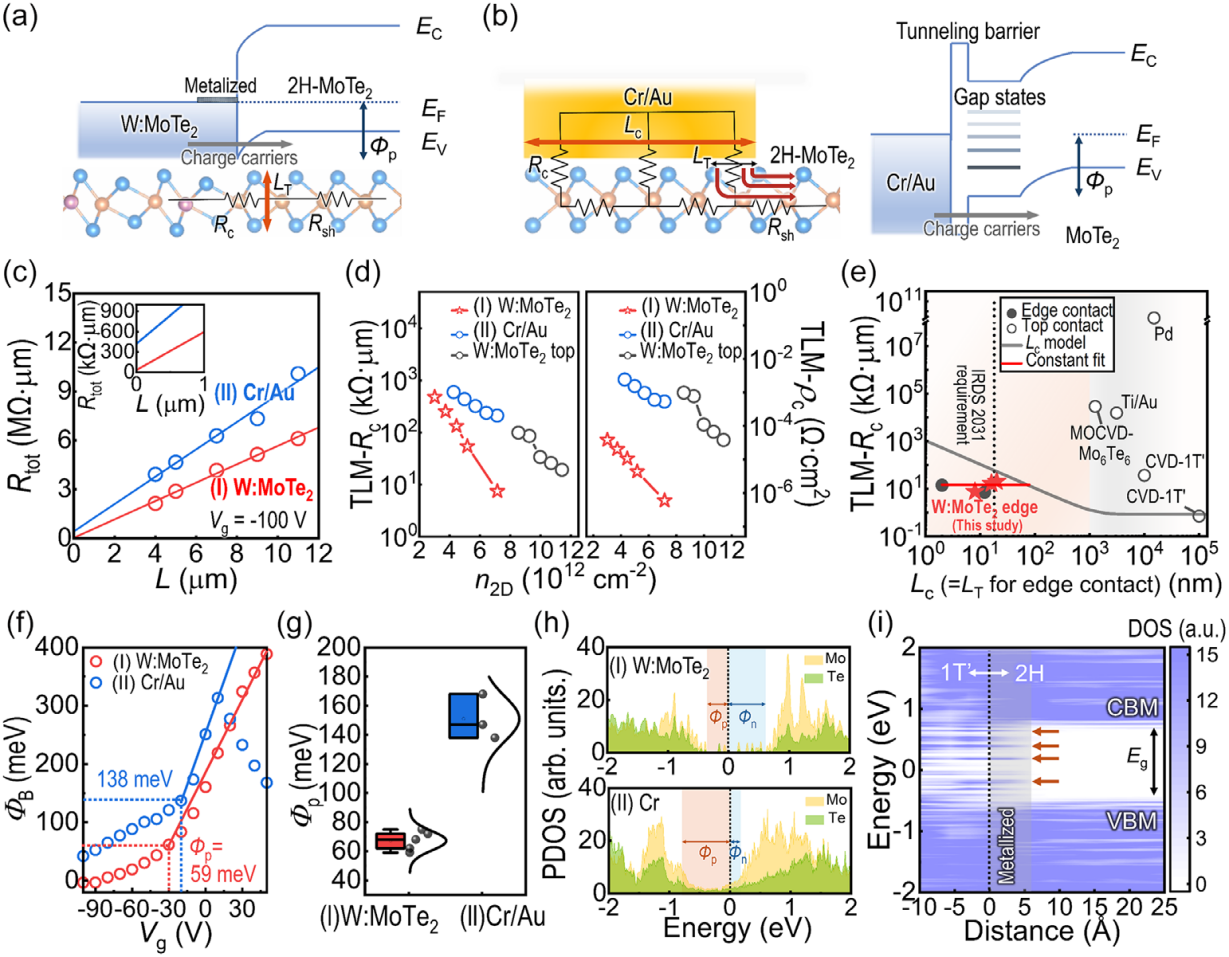
Electrical transport of edge‐ and top‐contact MoTe_2_ MSJ FETs. a,b) Illustration showing (a) W:MoTe_2_ atomically stitched with MoTe_2_ and (b) Au/Cr/MoTe_2_ top‐contact MSJ FET along with the resistance network and band diagram. c) TLM plots of W:MoTe_2_‐MoTe_2_ edge‐contact ((l) W:MoTe_2_) and Cr/Au top‐contact ((ll) Cr/Au) MSJ FETs at *V*_g_ = −100 V, demonstrating the linear dependence of the total resistance (*R*_tot_) on channel length (*L*). d) Contact resistance (*R*_c_) and contact resistivity (*ρ*_c_) of MoTe_2_ edge‐ and top‐contact MSJ FETs depending on the sheet carrier concentration (*n*_2D_) induced by applied *V*_g_. The red star, blue and gray circles represent (l) W:MoTe_2_ edge, (ll) Cr/Au top, and W:MoTe_2_ top contact, respectively. e) Comparisons of extracted *R*_c_ with various studies for MoTe_2_ FETs,^[^ ^22^, ^23^, ^24^, ^28^, ^44^, ^45^, ^46^ ^]^ as a function of contact length (*L*_c_) or transfer length (*L*_T_). For edge‐contact devices, *L*_c_ can be scaled to *L*_T_ because charge transfer is limited by *L*_T_;^[^ ^11^, ^43^, ^53^, ^54^ ^]^ therefore, we plot data points of *L*_T_‐dependent *R*_c_ for edge contact devices. The dot line indicates the target *L*_c_ set for 2031 according to International Roadmap for Devices and Systems (IRDS).^[^ ^8^ ^]^ f) Extracted thermionic barrier height (Φ_B_) of MoTe_2_ FET with edge and top contacts as a function of *V*_g_. The extracted Φ_B_ at flat band voltage is regarded as a hole injection barrier (Φ_p_). g) Statistical plot showing Φ_p_ variations of edge‐ and top‐contact MoTe_2_ FETs. Each dataset corresponds to W:MoTe_2_ edge (67 ± 5.97 meV, *n* = 5) and Cr/Au top (151 ± 12.56 meV, *n* = 3). The statistical plot is presented as a box plot with gray dots representing individual measured data points and an overlaid distribution curve (see the Experimental Section for details). h) Projected density of states (PDOS) of W:MoTe_2_‐MoTe_2_ lateral heterojunction and Cr/MoTe_2_ vertical heterojunction. The Φ_p_ and Φ_n_ represent the Schottky barrier height for holes and electrons, respectively. i) Color‐contour plot representing PDOS at each layer of the W:MoTe_2_‐MoTe_2_ heterostructure in real space. Far from the interface, the MoTe_2_ retains its intrinsic band structure with a band gap (*E*_g_), containing conduction band minimum (CBM) and VBM. The red arrows indicate the MIGSs at the 2H‐MoTe_2_ crystal within a distance of ≈5.98 Å from the 1T’ structure (labeled as “metallized”; see Figure S34, Supporting Information).

The edge contact benefits from the in‐plane covalent bonding between the metal contact and semiconductor channel, which narrows the tunneling width and lowers *R*_c_, further enhancing carrier injection through the junction^[^ ^9^, ^16^ ^]^ (**Figure** **5**a). In contrast, top‐contact FETs often face a significant *R*_c_ at their MSJs owing to the MIGS,^[^ ^42^ ^]^ contact interfacial defects,^[^ ^6^ ^]^ and the existence of a vdW gap between the semiconductor and metal (which acts as an additional tunneling barrier).^[^ ^9^ ^]^ In addition, the considerable *R*_c_ and significant difference in *R*_sh_ between the contact metal and channel may confine charge transfer to the edge of the top,^[^ ^43^ ^]^ leading to an exponential increase in *R*_c_ when *L*_c_ is shorter than the carrier transfer length (*L*_T_) at the MSJ. This phenomenon, known as the “current crowding effect” (red arrows in Figure 5b),^[^ ^11^ ^]^ can therefore limit the *L*_c_ scaling of top‐contact FETs.

To evaluate the impact of the contact geometry on *R*_c_ and *L*_c_ scaling, we compare W:MoTe_2_‐edge‐contact MSJ FETs with top‐contact Cr/Au FETs using the TLM (Figure 5c,d). The on‐state *R*_c_ values for representative edge‐contact and top‐contact FETs were calculated to be 7.5 and 211 kΩ μm, respectively, when the channel thickness (*T*_ch_) was comparable (8–10 nm) (Figure 5c and S29, Supporting information; see the *T*_ch_ dependence in Figure S30, Supporting information). Note that the edge‐contact FETs with different device dimensions and shapes also revealed the comparable low *R*_c_ of 8 kΩ·μm, validating its reproducibility (Figure S31, Supporting information). As different contact areas of edge and top contacts may result in an unfair comparison, we calculated the contact resistivities (*ρ*_c_) by considering the feature sizes of different contact geometries (Figure 5d; see the Experimental Section for more details on the calculation). The *ρ*_c_ of the edge‐contact MSJ (≈5.9 × 10^−7^ Ω cm^2^) is approximately three orders of magnitudes lower than that of top‐contact MSJ (≈5.3 × 10^−4^ Ω cm^2^), while the *R*_c_ of edge contact is ≈30 times lower than that of the Cr/Au top contact. Moreover, this trend is observable in top‐contact FETs fabricated by transferring 2D 1T’‐W:MoTe_2_ onto 2H‐MoTe_2_ (gray symbols in Figure 5d; see more details in Figure S32, Supporting information), as the top‐contact devices exhibit a *ρ*_c_ higher by a factor of 64 (3.8 × 10^−5^ Ω cm^2^) compared with the edge‐contacted ones. The smaller *ρ*_c_ of the edge contact indicates that it allows for better carrier injection efficiency, compared with top contact.

Compared with other (MO)CVD‐grown MoTe_2_ FETs,^[^ ^22^, ^23^, ^24^, ^28^, ^44^, ^45^, ^46^ ^]^ our one‐step edge‐contact FETs exhibit relatively low *R*_c_ (7.5 kΩ μm). As carriers are injected across the channel thickness in edge‐contact FETs, the effective contact length^[^ ^11^ ^]^ is considered equivalent to ultrashort *L*_T_ (=*T*_ch_) of 8 nm (Figure 5e and **Note in** Table S2, Supporting information). The solid line in Figure 5e represents an transmission line model fit to the record‐low *R*_c_ value (0.7 kΩ μm) observed in a top‐contact polymorphic 1T’/2H‐MoTe_2_ MSJ FET,^[^ ^28^ ^]^ with an *L*_c_ of 100 μm (see the Experimental Section). This *R*_c_‐*L*_c_ model predicts that higher *R*_c_ values exceeding 100 kΩ μm are expected for the vertical heterostructure FET when *L*_c_ is less than 10 nm. Hence, the MoTe_2_ edge‐contact FET in this study is advantageous for scaling *L*_c_, as it shows a smaller *R*_c_ of 7.5 kΩ μm at an effective *L*_c_ (=*L*_T_) of 8 nm. Note that our scalable fabrication scheme is distinctive, because the reported edge contacts for MoTe_2_ mostly rely on randomly grown 2D flakes, resulting in a scarcity of studies on electrical transport.^[^ ^24^, ^25^ ^]^

To further investigate the contact barrier properties of each FET set, we conducted the temperature (*T*)‐dependent electrical measurements and extract thermionic barrier height (Φ_B_) of the MSJs using the 2D thermionic emission model (Figure S33, Supporting information; see the Experimental Section for more details). From the slope of the Arrhenius plot (*ln*(*I*_ds_/*T* ^3/2^ *vs.* 1000/*T*; Figure S33b,d, Supporting information), we obtained Φ_B_ as a function of the applied *V*_g_ (Figure 5f). The Schottky barrier height (SBH) for hole transport (Φ_p_) is determined at the flat band condition (indicated as dashed lines in Figure 5f), which is ≈59 meV for the W:MoTe_2_ edge‐contact MSJ and ≈138 meV for the Cr/Au top‐contact MSJ. For various devices, the smaller average value of Φ_p_ for edge‐contact FET (≈67 meV) compared with top‐contact FET (≈151 meV) is consistently observed (Figure 5g).

The smaller Φ_p_ of the edge‐contact MSJ is also confirmed by DFT calculations (Figure 5h,i). The projected DOS (PDOS) plot of W:MoTe_2_ lateral heterostructure exhibits a smaller Φ_p_ (≈0.36 eV) compared with the Cr top‐contact MSJ (≈0.78 eV) at zero bias in the ground state (Figure 5h). In addition, the small DOS of the semimetal W:MoTe_2_ resulted in negligible MIGS in the edge‐contact MSJ (Figure S34, Supporting information), in contrast to the Cr top‐contact MSJ. In combination with the suppressed MIGSs, modulation of the work function of the 2D semimetal through gate electrostatics^[^ ^10^, ^47^ ^]^ may allow for gate‐tunability of Φ_p_, leading to effective hole transport when negative *V*_g_ is applied (Figure S35, Supporting information).

The suppressed MIGS was also evidenced by the contour plot of the PDOS in real space for the edge‐contact heterostructure (Figure 5i). The 2H‐MoTe_2_ channel is minimally impacted by MIGS, with metallization affecting only the atoms within ≈5.98 Å of the edge interface. In contrast, the rest of the 2D channel remained largely unperturbed, maintaining its intrinsic band structure. Considering the Mo—Te bond length of ≈2.79 Å,^[^ ^32^ ^]^ the interfacial dipoles caused by MIGS are confined to a few atomic distances and decay rapidly away from the interface, which results in partial depinning of the Fermi level.^[^ ^15^, ^39^ ^]^ Consequently, the efficient hole transport in the W:MoTe_2_ edge‐contact MSJ is primarily attributed to the contact geometry with an ultrashort metallized interface, which enables gate‐tunable Φ_p_.

## 3 Conclusions

In summary, we have reported a method to construct edge‐contact p‐type FETs by controlling the phase of 2D MoTe_2_. An alloying‐mediated selective‐area phase control enables simultaneous growth for both semi‐metallic (1T’‐W_*x*_Mo_1−*x*_Te_2_) and semiconducting (2H‐MoTe_2_) electronic components to be free from the oxidation at the edge of interface and misalignment issues, while maintaining a high‐quality 2D single‐crystalline channel. Our W_*x*_Mo_1−*x*_Te_2_ edge‐contact FETs significantly outperform devices fabricated with two‐step metallization, showing improved *I*_on_ and *I*_on_/*I*_off_. Additionally, at a carrier transfer length below 10 nm, atomically clean MSJ interface results in low contact resistivity of ≈5.9 × 10^−7^ Ω cm^2^, which is about three orders of magnitudes lower than that of 3D metal top‐contact FET. The efficient hole transport in our one‐step edge‐contact structure is attributed to not only the clean interface of MSJ but also the suppressed gap states derived from the low DOS of the semimetal W_*x*_Mo_1−*x*_Te_2_. Therefore, this study on the fabrication of p‐type edge contacts with 2D semimetal electrode paves way to help bridge the gap between n‐ and p‐type 2D FETs, suggesting their potential for the advancement of miniaturized 2D CMOS electronics.

## 4 Experimental Section

4.1

4.1.1

##### Fabrication of Edge‐Contact W:MoTe_*2*_ *‐MoTe*_*2*_ *MSJ FETs*

The patterned W/Mo precursor films were first fabricated using a sequential DC magnetron sputtering process. Initially, a Mo film was uniformly deposited on a 300 nm‐thick SiO_2_/Si substrate at a power of 700 W for less than 5 s, depending on the desired thickness of MoTe_2_. Subsequently, to define the patterned W regions, photolithography (Midas, MDA‐400S) was used to spatially define the desired areas, followed by W deposition via DC sputtering at a lower power of 300–400 W for 1 s. All DC sputtering processes were carried out under an Ar atmosphere at 10 mTorr with a flow rate of 100 sccm. Note that, for a successful lift‐off process, image‐reversal photoresist (AZ5214E) was used to create an undercut. For position‐controllability, the maskless laser writer (Durham, ML3 Pro) also enabled application of universal patterns for one‐step edge contact fabrication without the need for a hard mask.

The edge‐contact polymorphic heterojunctions were then grown by the tellurization of patterned W/Mo thin film precursors in a hot‐wall furnace system at 700 °C for 2 h with Ar/H_2_ (550/110 sccm) flow. The W/Mo/SiO_2_/Si sample was stacked face‐to‐face onto a Te source of a Ni_*x*_Te_*y*_ substrate and placed in a one‐side closed tube at the hot zone of the furnace. During growth, the Te vapors were confined in the gap between the films, facilitating the formation of high‐quality telluride films.^[^ ^27^, ^28^ ^]^ After growth, the MoTe_2_ channel region was defined using reactive ion etching (RIE) process with SF_6_ and O_2_ plasma to prevent any electrical transport through undesired areas. For the FETs, the underlying SiO_2_/Si was used as a bottom gate structure. During device fabrication, encapsulation using the photoresist was applied each time, and the air exposure time of the sample until the subsequent process was less than 15 min.

##### Fabrication of Two‐Step Metallized MoTe_*2*_ *MSJ FETs*

For all the two‐step metallized FETs, large‐area 2H‐MoTe_2_ thin films on SiO_2_/Si substrate were first grown by the tellurization of the Mo precursor (thickness of 1.5–3 nm) at 700 °C for 2 h.^[^ ^28^ ^]^ For edge‐contact FETs with semimetal electrodes (1T’‐MoTe_2_ or T_d_‐WTe_2_), the electrode regions were etched away using photolithography followed by the RIE technique. Next, deposition of Mo (W) precursors onto the exact etched region was conducted, followed by the tellurization at 500 °C (Figure S19, Supporting information). For the top‐contact FETs with Cr/Au (5/50 nm) contacts, metal electrodes were deposited using e‐beam evaporator (Woosung, WC‐4000). To minimize contact interface degradation during the high‐energy deposition process, the deposition rate was reduced to 0.1 Å s^−1^ during the deposition of initial 5 nm, and then increased to 0.5 Å s^−1^ in a high vacuum (10^−6^ Torr). After metallization, 2H‐MoTe_2_ channel width was defined using the RIE process.

##### Structural Characterizations

Raman measurements were conducted using a micro‐Raman spectrometer (Witec, alpha300R) with 532 nm laser excitation focused through a 50×, 0.8 NA objective lens and 1800 lines mm^−1^ grating. Raman spectra under the Raman shift of ≈150 cm^−1^ (Figure 1f and S13, Supporting information) were acquired using a Raman spectrometer (Horiba LabRAM HR) with a 100×, 0.9 NA objective and a 2400 lines mm^−1^ grating. A solid‐state 514.7 nm laser was used for excitation with a laser power of 100 μW. The XRD measurements were performed using Bruker, AXS D8 equipped with a Cu K source for structural characterization, and accurate XRD peak positions were extracted by fitting to Gaussian function. The AFM images were obtained in tapping mode using the Bruker Dimension Icon, while KPFM measurements were conducted with the same apparatus using Pt/Ir probes. High‐resolution STEM images, SAED patterns, and EDS were obtained using the aberration‐corrected FEI Titan^3^ G2 60‐300 at an acceleration voltage of 200 kV. STEM images were captured using a HAADF detector to collect semi‐angles from 50.5 to 200 mrad. A Wiener filter was applied to reduce noises in the high‐resolution STEM images. Commercially available software, TEMPAS (Total Resolution), was used for multislice STEM image simulation. For the cross‐sectional HAADF images, the samples were prepared using a focused ion beam (FEI Helios NanoLab 450). The EBSD characterizations were conducted by SEM (FEI Verios 460) equipped with EBSD detector (AMTEK, Inc., Hikari).

##### Electrical Measurements

Electrical characterization was performed in a cryogenic probe station (Lakeshore CRX‐4 K) equipped with a Keithley 4200‐SCS detector at different temperatures and a high vacuum of 10^−4^ torr. The field‐effect mobilities (*μ*_FE_) of MoTe_2_ FETs with different contacts were calculated using the equation $\mu_{FE}=\left(\frac{dI_{ds}}{dV_{g}}\right)\left(\frac{L}{WC_{i}V_{ds}}\right)$, where *W*, *L*, and *C*_i_ represent the channel width, length, and gate dielectric capacitance (*C*_i_ of ≈11.5 nF cm^−2^, 300 nm SiO_2_), respectively. The sheet carrier density (*n*_2D_) was calculated using a parallel capacitance model as *n*_2D_ = *C*_i_(*V*_g_‐*V*_th_)/*q*, where *V*_th_ and *q* are the threshold voltage and elementary charge, respectively. *V*_th_ was obtained via linear extrapolation from the transfer curve (*V*_g_‐*I*_ds_).

##### First‐Principles Calculations

First‐principles calculations were performed using QuantumATK^[^ ^48^ ^]^ to compare the edge‐contact W:MoTe_2_‐MoTe_2_ MSJ and top‐contact Cr/MoTe_2_ MSJ systems. For both systems, geometry optimization was performed using a linear combination of atomic orbitals basis sets, with the norm‐conserving PseudoDojo pseudopotential method^[^ ^49^ ^]^ and generalized gradient approximation (GGA) in the form of the Perdew–Burke–Ernzerhof functional.^[^ ^50^ ^]^ The convergence criteria for the total energy and force were set to 10^−4^ eV and 0.05 eV Å, respectively. A vacuum slab of at least 30 Å was applied along the z‐direction (and y‐direction for edge‐contacted structures) with Neumann–Dirichlet boundary conditions. The Grimme DFT‐D3 correction method^[^ ^51^ ^]^ was used to describe the van der Waals correction.

The W:MoTe_2_ structure was constructed using a 2 × 5  ×1 supercell of a monoclinic 1T’‐MoTe_2_ unit cell, and the Mo atoms were randomly replaced with W atoms to adjust the W_0.15_Mo_0.85_Te_2_ stoichiometry. A 5 × 16 × 1 replicate of the monolayer monoclinic unit cell MoTe_2_ and 1 × 2 × 1 replicate of the defined monolayer W_0.15_Mo_0.85_Te_2_ cell were considered to establish the W:MoTe_2_‐MoTe_2_ edge‐contact heterostructure, with an equal strain of 1.68%. The density mesh cutoff was set to 60 Hartree, where 2 × 1 × 1 and 3 × 1 × 1 k‐point meshes were generated using the Monkhorst‐Pack method^[^ ^52^ ^]^ for geometry optimization and electronic calculations, respectively. For the top‐contact Cr‐MoTe_2_ heterostructure, a 2$\sqrt{2}\times$ $\sqrt{2}$ × 1 replicate of monolayer hexagonal unit cell MoTe_2_ and $\sqrt{13}\times$ $\sqrt{5}$× 1 replicate of 5‐layer body‐centered cubic unit cell Cr were chosen with an equal strain of 1.32%. A 4 × 5 × 1 and 7 × 8 × 1 k‐point meshes were selected for geometry optimization and electronic calculations, respectively. The density mesh cut‐off was set to 105 Hartree.

##### Transmission Line Model

The transmission line model helps determine the transfer length (*L*_T_), which is the characteristic length over which the current is injected. The model is expressed as^[^ ^5^ ^]^

(2) $$R_{C}=\sqrt{\rho_{c}R_{sh}}\coth\left(\frac{L_{c}}{L_{T}}\right)\approx \sqrt{\rho_{c}R_{sh}}$$

Here, *ρ*_c_ and *R*_sh_ are obtained from the TLM plots. The *L*_T_ is defined as $L_{T}=\sqrt{\frac{\rho_{c}}{R_{sh}}}$. For a fair comparison of the contact resistivity across FETs with different contact schemes, we must consider the effective interfacial area of the contacts. For the top‐contact MSJs, the effective interfacial area can be expressed as *L*_T_ × *W*. For the edge‐contact MSJ, because the majority carriers are injected through the interfacial side area, the effective interfacial area can be approximated as the product of the channel thickness and width (*t* × *W*).^[^ ^53^ ^]^ Thus, the specific contact resistivity is calculated as; *ρ*_c_ = *R*_c_·*W*·*L*_T_ for top contact and *ρ*_c_ = *R*_c_·*W*·*t* for edge contact.

##### Schottky Barrier Height Extraction

To calculate the thermionic barrier height (Φ_B_) of MSJs, the 2D thermionic emission model is used^[^ ^5^ ^]^

(3) $$I_{ds}=A_{2D}^{*}T^{3/2}\text{exp}\left(-\frac{{Ø}_{B}}{k_{B}T}\right)\left[1-\text{exp}\left(-\frac{V_{ds}}{k_{B}T}\right)\right]$$

where $A_{2D}^{*}$ is the Richardson‐Boltzmann constant for the 2D system, and *k*_B_ is the Boltzmann constant. If *V*_*ds*_ ≫ *k*_*B*_T, this simplifies to

(4) $$I_{ds}\approx A_{2D}^{*}T^{3/2}\text{exp}\left(-\frac{{Ø}_{B}}{k_{B}T}\right)$$

The Schottky barrier heights were extracted at the *V*_g_ value equal to the flat band voltage (*V*_FB_), which is the endpoint of the linear dependence of the Φ_B_‐*V*_g_ plot.

##### Statistical Analysis

Statistical plots of field‐effect mobility (*μ*_FE_) for MoTe_2_ FETs with different contact configurations and thermionic barrier heights for hole injection (Φ_p_) in edge‐ and top‐contact MoTe_2_ FETs are presented as box plots (Figure 4n and 5g). Each box represents the interquartile range (IQR), with the central line indicating the median value, while the whiskers extend to the minimum and maximum data points within 1.5 × IQR. The black open circles inside each box represent the mean values, and the gray dots represent individual measurements. Kernel density curves are shown adjacent to each box, illustrating the probability density of the data distribution. For each dataset, the mean ± standard deviation (SD) and the number of samples (*n*) are provided in the corresponding figure captions. Data processing and visualization were performed using OriginPro 2020 (OriginLab, USA).

## Supporting Information

Supporting Information is available from the Wiley Online Library or from the author.

## Conflict of Interest

The authors declare no conflict of interest.

## Supporting information

Supplementary Material

## Data Availability

The data that support the findings of this study are available from the corresponding author upon reasonable request.
