# Supplementary material for: Wafer‐Scale Fabrication of Edge‐Contacted Nanosheet Transistors via Alloying‐Mediated Phase Engineering
Source: Small Sci. 2025 Oct 30;5(12):e202500320. doi: 10.1002/smsc.202500320 (PMC12697875; doi:10.1002/smsc.202500320)
Supplement: Supplementary file 1 — Supplementary Material [file SMSC-5-e202500320-s001.pdf]

Supporting information

Wafer-Scale Fabrication of Edge-Contacted Nanosheet Transistors via Alloying-Mediated Phase Engineering

Sora Jang[1], Seunguk Song[2,3*], Juwon Han[1], Aram Yoon[1,4], Jaewon Wang[1], Hyeonwoo Lee[1], Young Ho Jin[1], Yeoseon Sim[1], Zonghoon Lee[1,4], Changwook Jeong[1*], and Soon-Yong Kwon[1*]

[1]*Department of Materials Science and Engineering & Graduate School of Semiconductor Materials and Devices Engineering, Ulsan National Institute of Science and Technology (UNIST), Ulsan 44919, Republic of Korea*
[2]*Department of Energy Science, Sungkyunkwan University (SKKU), Suwon 16419, Republic of Korea*
[3]*Center for 2D Quantum Heterostructures (2DQH), Institute for Basic Science (IBS), Suwon 16419, Republic of Korea*
[4]*Center for Multidimensional Carbon Materials (CMCM), Institute for Basic Science (IBS), Ulsan 44919, Republic of Korea*

*E-mail: seunguk@skku.edu (S.S.), changwook.jeong@unist.ac.kr (C.J.) and sykwon@unist.ac.kr (S.-Y.K.)

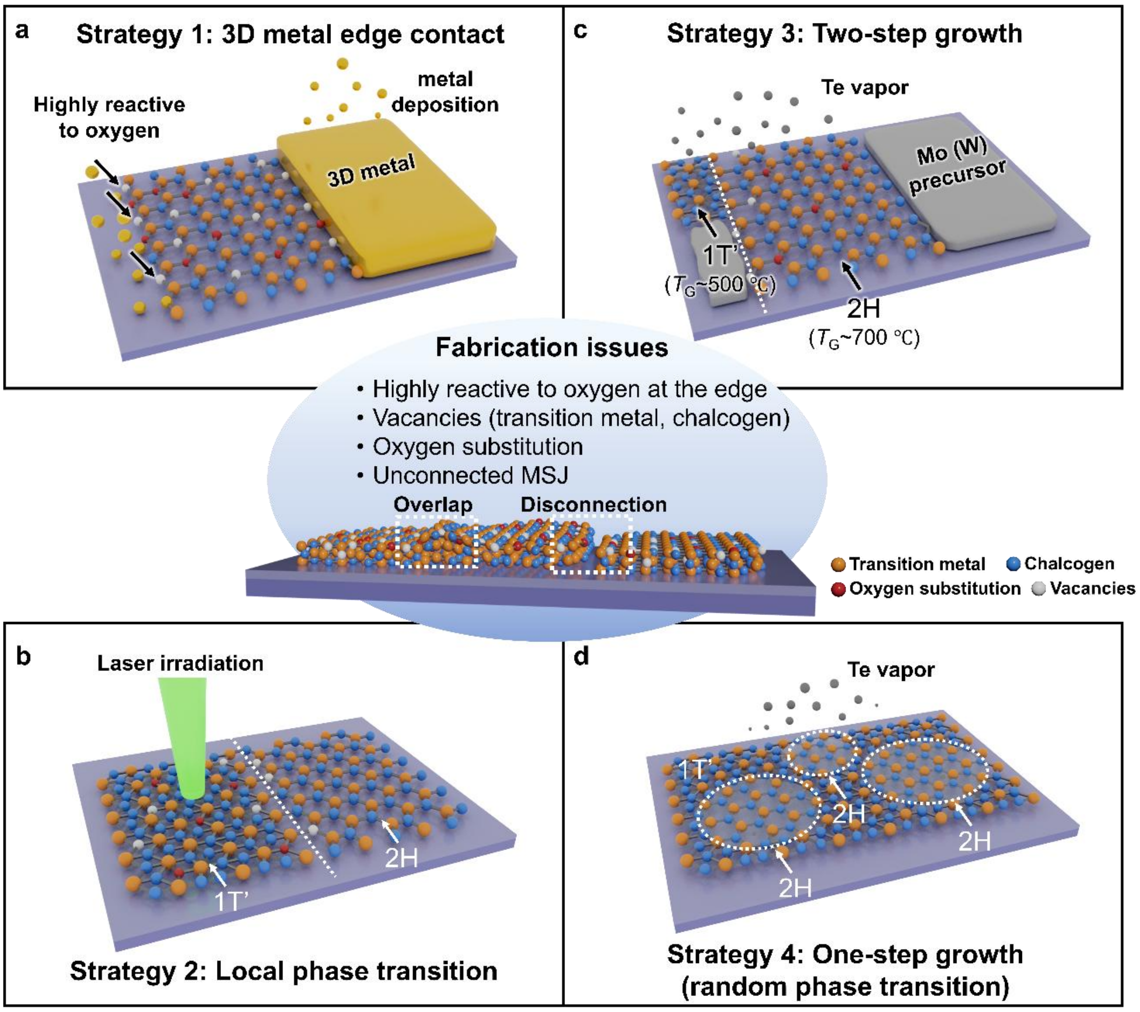

Figure S1. Challenges in constructing edge-contact 2D FETs. (**a,b**) Top-down approaches involve the synthesis of semiconducting material, followed by post-processing to establish contact electrodes. (**a**) Side-etching of the 2D channel material and 3D metal deposition for constructing edge contact.[1,2] (**b**) Laser irradiation for a local phase transition from 2H-$MoTe_2$ to 1T'-$MoTe_2$.[3,4] (**c,d**) Bottom-up approaches for the synthesis of semiconducting and metallic 2D structures. (**c**) Two-step growth method for constructing edge-contact FETs comprising of electronic components with different growth temperature (T_G). The process begins with the synthesis of 2H-$MoTe_2$, followed by selective area etching. Then, Mo (W) metal thin films are deposited for electrodes, and a second growth process is applied for metallic TMDs.[5,6] (**d**) One-step growth method in which the metallic and semiconducting TMDs are synthesized simultaneously. This approach achieves edge contact by controlling the growth parameters such as growth temperature[7-9] and deposited precursor metal thin films.[10] The method shown in (d) illustrates random phase transition during growth of $MoTe_2$ polymorphs.

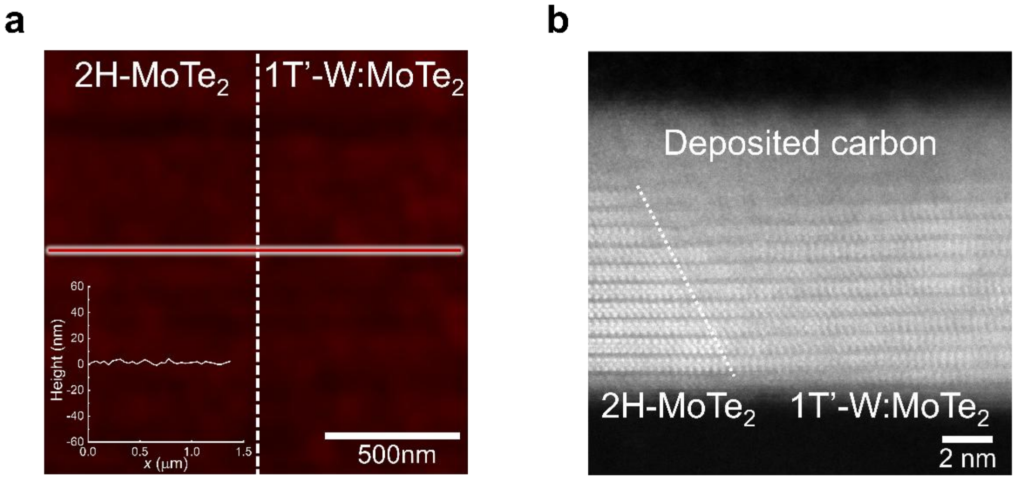

Figure S2. W:MoTe$_2$-MoTe$_2$ heterostructure with negligible thickness difference at the interface. (**a**) AFM image of the heterojunction, with the corresponding height profile displayed in the bottom-left inset. The dashed line indicates the heterointerface between two phases, and the red solid line outlines the region for the height profile. (**b**) Cross-sectional HAADF-STEM image.

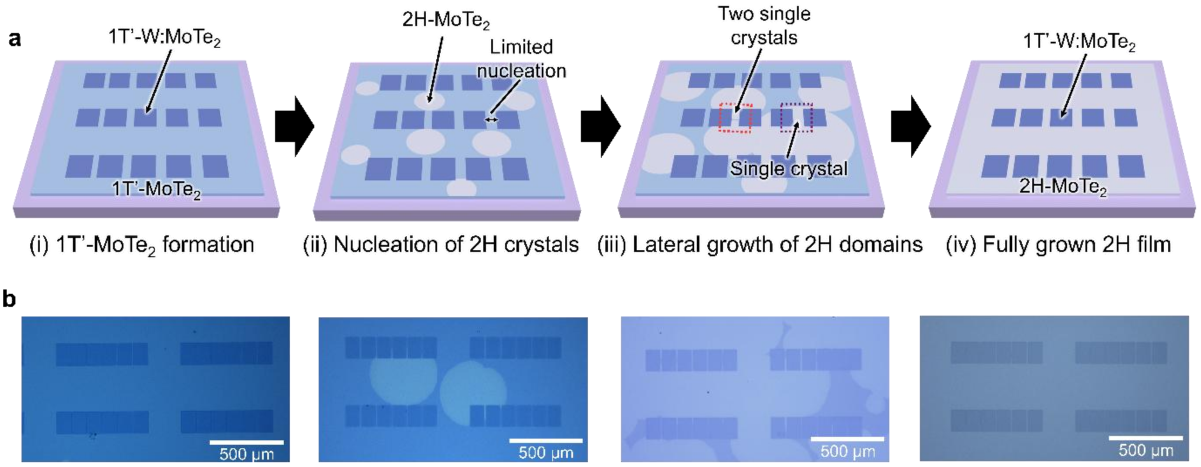

Figure S3. Morphology evolution and growth behavior of 2H-$MoTe_2$ single grain with increasing temperature and growth time. (**a**) Schematics illustrating the evolution of 2H single grains in W:$MoTe_2$-$MoTe_2$ heterostructure. Each stage represents; (i) formation of 1T'-$MoTe_2$ at a growth temperature (T_G) of 500 °C; (ii) emergence of 2H nucleation as T_G increases to 700 °C—nucleation occurs predominantly in the background region, while it is suppressed in the narrow separation between the electrodes due to the spatial confinement; (iii) lateral growth of 2H single grains at maintained T_G of 700 °C; and (iv) merging of all 2H single grains and formation of a continuous 2H-$MoTe_2$ thin film. (**b**) OM images corresponding to each stage.

The growth of 2H-$MoTe_2$ occurs via a phase transition from 1T'-$MoTe_2$ during the tellurization process.[11,12] The Mo-deposited region was converted into 2H-$MoTe_2$, whereas 2H nucleation was suppressed in the W/Mo deposited region (**Figure 1e**). During this work, we also found that the narrow separation between W:$MoTe_2$ electrodes (≤ 11 μm) suppresses the nucleation of the 2H phase (**Figure S3**); therefore, the 2H conversion only occurs outside this narrow gap. Because the single-grain size of the 2H phase exceeds 100 μm, one grain is sufficient to cover the channel region without grain boundaries. Note that there is a small possibility that grain boundaries forming parallel to the channel within the channel region (i.e., two single crystals at the channel); however, their impact on electrical transport is negligible compared with vertical grain boundaries.[13,14] See further investigation of the single-crystalline nature of 2H-$MoTe_2$ in **Figure S4**, **S5** and **Table S1**.

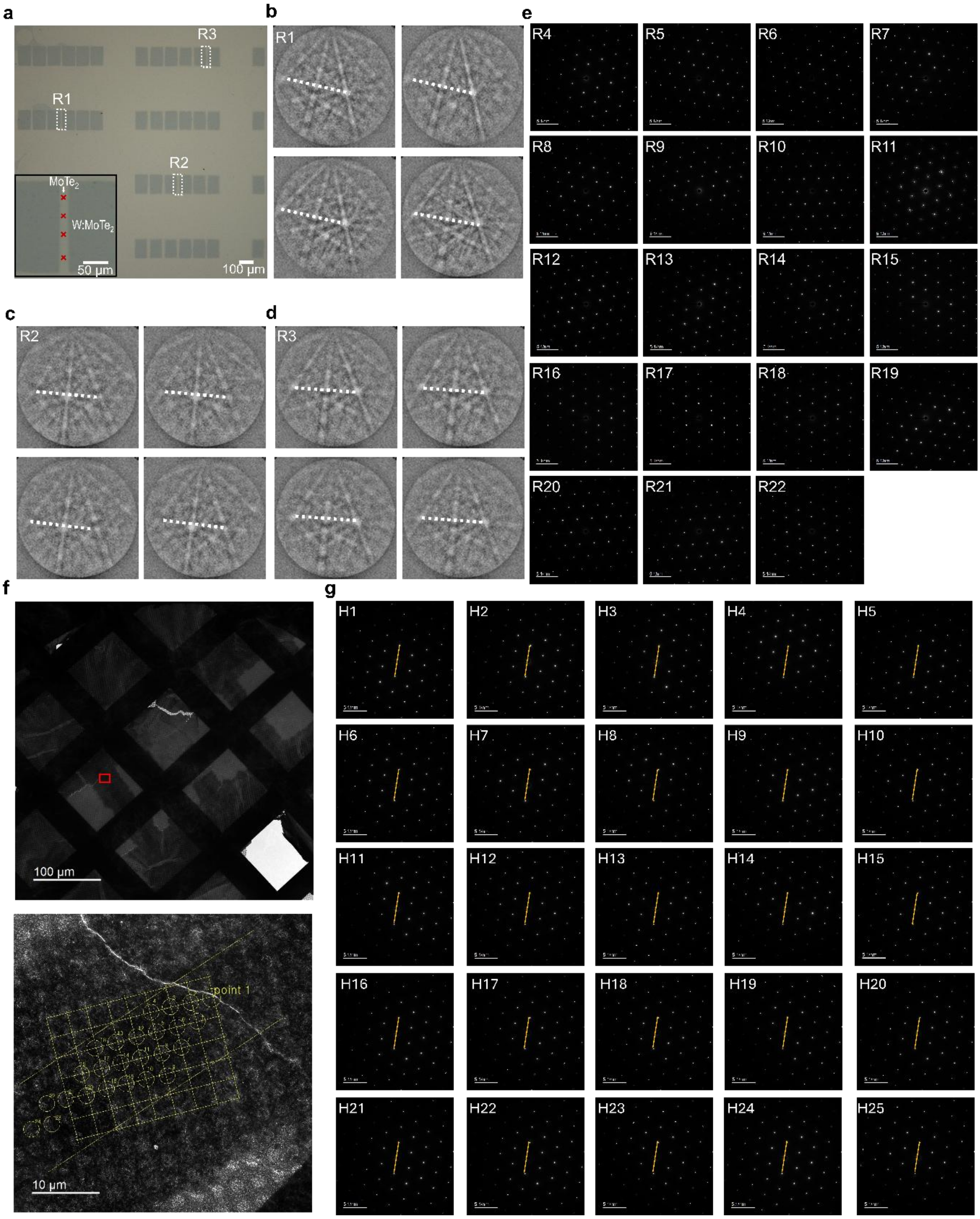

Figure S4. Characterizations of large-area single-crystalline $MoTe_2$ channel. (**a**) OM image of 2H $MoTe_2$-W:$MoTe_2$ heterostructure arrays. The inset displays a zoomed-in image of the dotted box. Red crossbars indicate the measurement points in each region (R). (**b-d**) Representative EBSPs of 2H-$MoTe_2$ from different channel regions. (**e**) Representative SAED patterns acquired from randomly selected positions in 19 different channel regions. (**f,g**) SAED patterns captured from a single channel region. (**f**) Low-magnification TEM image of the $MoTe_2$-W:$MoTe_2$ array on a TEM grid. (**g**) SAED patterns obtained at each hole of the TEM grid. The aperture size for SAED pattern is 1 μm.

The single-crystalline nature of the synthesized 2H-$MoTe_2$ was further confirmed by EBSD and TEM analysis (**Figure S4**). EBSPs were obtained from randomly selected channel regions within a 1 × 1 cm^2 area of the W:$MoTe_2$-2H $MoTe_2$ heterostructure film (**Figures S4a-d**). All EBSPs collected from each channel region exhibited the same Kikuchi patterns, confirming the single crystallinity of 2H-$MoTe_2$ in the channels. Furthermore, SAED patterns obtained through TEM analysis from 19 different locations using a 1 μm aperture consistently revealed the single crystalline of 2H-$MoTe_2$ (**Figure S4e**). In addition, the 25 SAED patterns collected from one representative channel region showed the same orientation (**Figures S4f,g**).

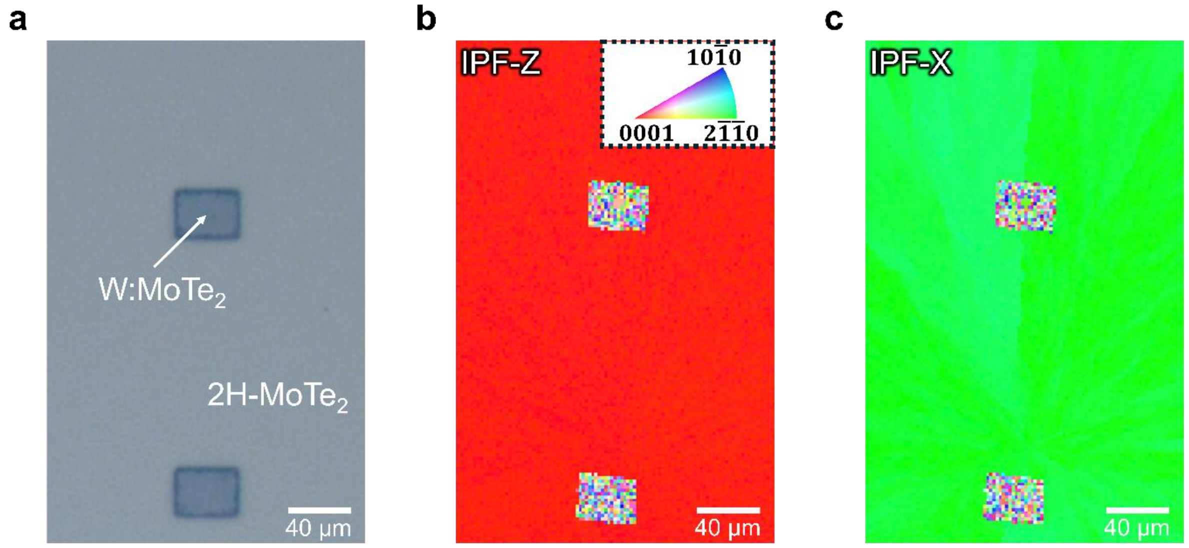

Figure S5. EBSD characterization of W:$MoTe_2$-$MoTe_2$ heterostructure. (**a**) Optical microscopy (OM) image of the W:$MoTe_2$-$MoTe_2$ heterostructure. The square-patterned region and background region correspond to 1T'-W:$MoTe_2$ and 2H-$MoTe_2$, respectively. (**b**) Inverse pole figure (IPF) map of the W:$MoTe_2$-$MoTe_2$ heterostructure in the out-of-plane direction (IPF-Z). (**c**) IPF map of the W:$MoTe_2$-$MoTe_2$ heterostructure in the in-plane direction (IPF-X). The IPF maps reveal the unidirectionally aligned orientation of single-crystalline 2H-$MoTe_2$ over hundreds of micrometres.

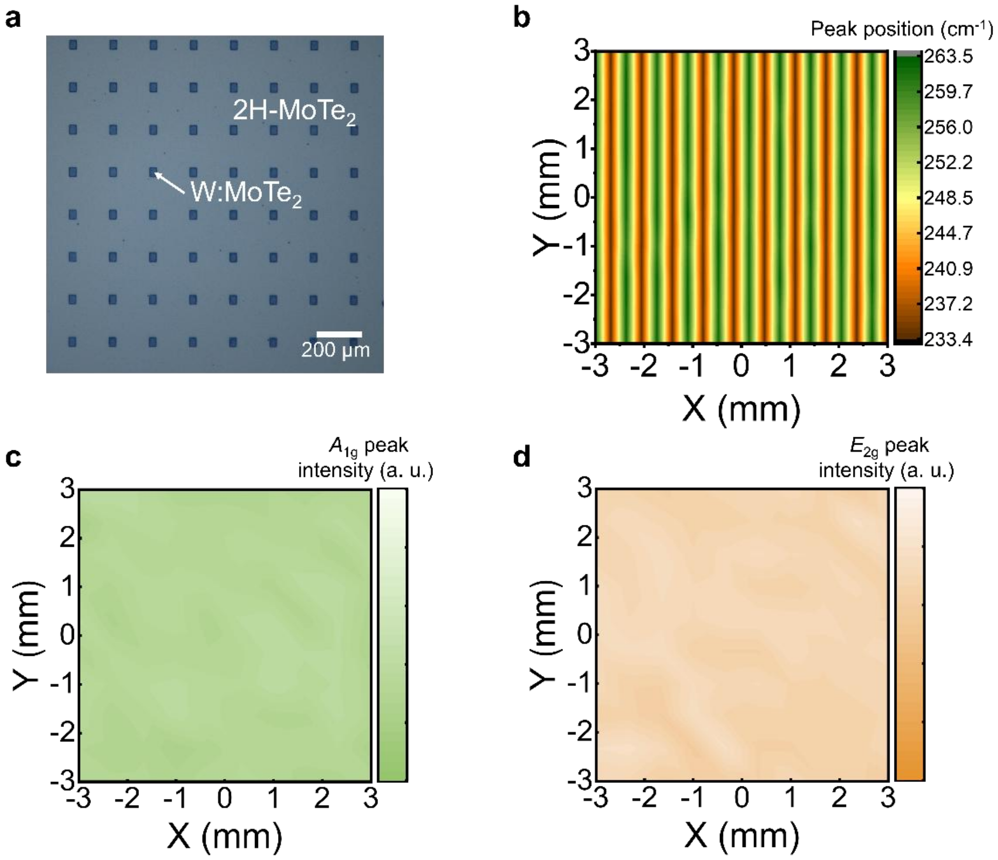

Figure S6. Raman mapping of W:$MoTe_2$-$MoTe_2$ heterostructure. (**a**) OM image of W:$MoTe_2$-$MoTe_2$ heterostructure. The patterned square regions and background consist of 1T'-W:$MoTe_2$ and 2H-$MoTe_2$, respectively. (**b**) Raman mapping image of W:$MoTe_2$-$MoTe_2$ heterostructure with green and orange color corresponding to the distinct vibration modes of A_{1g} at 260 cm^{-1} (for 1T'-W:$MoTe_2$) and E_{2g} at 233 cm^{-1} (for 2H-$MoTe_2$). (**c,d**) Mapping images representing the intensity of A_{1g} peak (**c**), and E_{2g} peak (**d**), demonstrating uniform growth of 1T'-W:$MoTe_2$ and 2H-$MoTe_2$, respectively.

We conducted Raman mapping analysis to confirm the spatial controlled W:$MoTe_2$-$MoTe_2$ heterostructure and its uniformity, as shown in **Figure S6**. We investigated different Raman vibrational modes depending on each phase (A_{1g} at ~260 cm^{-1} and E_{2g} at ~233 cm^{-1} for 1T'-W:$MoTe_2$ and 2H-$MoTe_2$, respectively), and corresponding Raman spectra were additionally mapped across a 6 × 6 mm^2 area, consisting of 1T'-W:$MoTe_2$ and 2H-$MoTe_2$ regions (**Figure S6a**). We collected Raman spectra from 10 points of each polymorph along a line repeatedly across 10 different lines (i.e., 100 Raman spectra acquired for each phase) (**Figures S6b-d**).

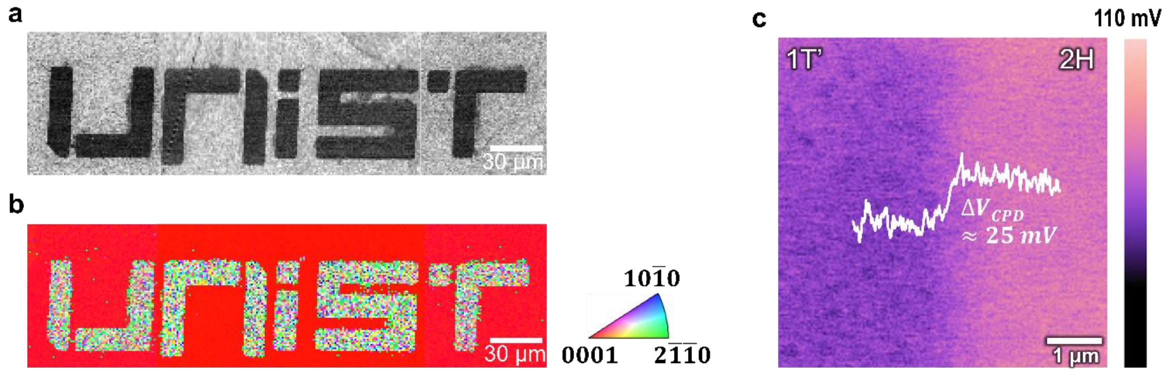

Figure S7. Spatially controlled synthesis of 1T'- and 2H- phase $MoTe_2$. (**a**,**b**) Electron backscattered diffraction (EBSD) characterization for as synthesized W:$MoTe_2$-$MoTe_2$ lateral heterostructure in **Figure 1d**. The "UNIST" letters and the background consist of 1T'-W:$MoTe_2$ and 2H-$MoTe_2$, respectively. (**a**) EBSD image quality (IQ) map. (**b**) Normal direction inverse pole figure (IPF) map. (**c**) Surface potential image and potential line profile of W:$MoTe_2$-$MoTe_2$ interface measured by Kelvin probe force microscopy (KPFM). The potential difference of ≈25 mV is indicated in the surface potential profile (white lines).

To distinguish between the different phases in the W:$MoTe_2$-$MoTe_2$ lateral heterostructure, EBSD and KPFM measurements were conducted. The IQ map image reveals a dark region in the "UNIST" letters, signifying a lack of contrast in the Kikuchi bands of 1T'-W:$MoTe_2$ (**Figure S7a**). This darker contrast can be attributed to the grain size of the 1T' structure (< 100 nm[12]) being smaller than the detection limit of ~120 nm,[15] along with a high dislocation density (> ~10^{10} cm/cm^3).[15,16] Additionally, the IPF map demonstrates variations in crystal structure between the phases (**Figure S7b**). The uniform red color ([0001] crystal orientation) reveals that the 2H-$MoTe_2$ layers are well-ordered along the normal direction, consistent with the expected behavior of a single crystalline nature.[12] In contrast, the IPF map does not show the clear features for 1T'-$MoTe_2$, as it falls below the detection limit.

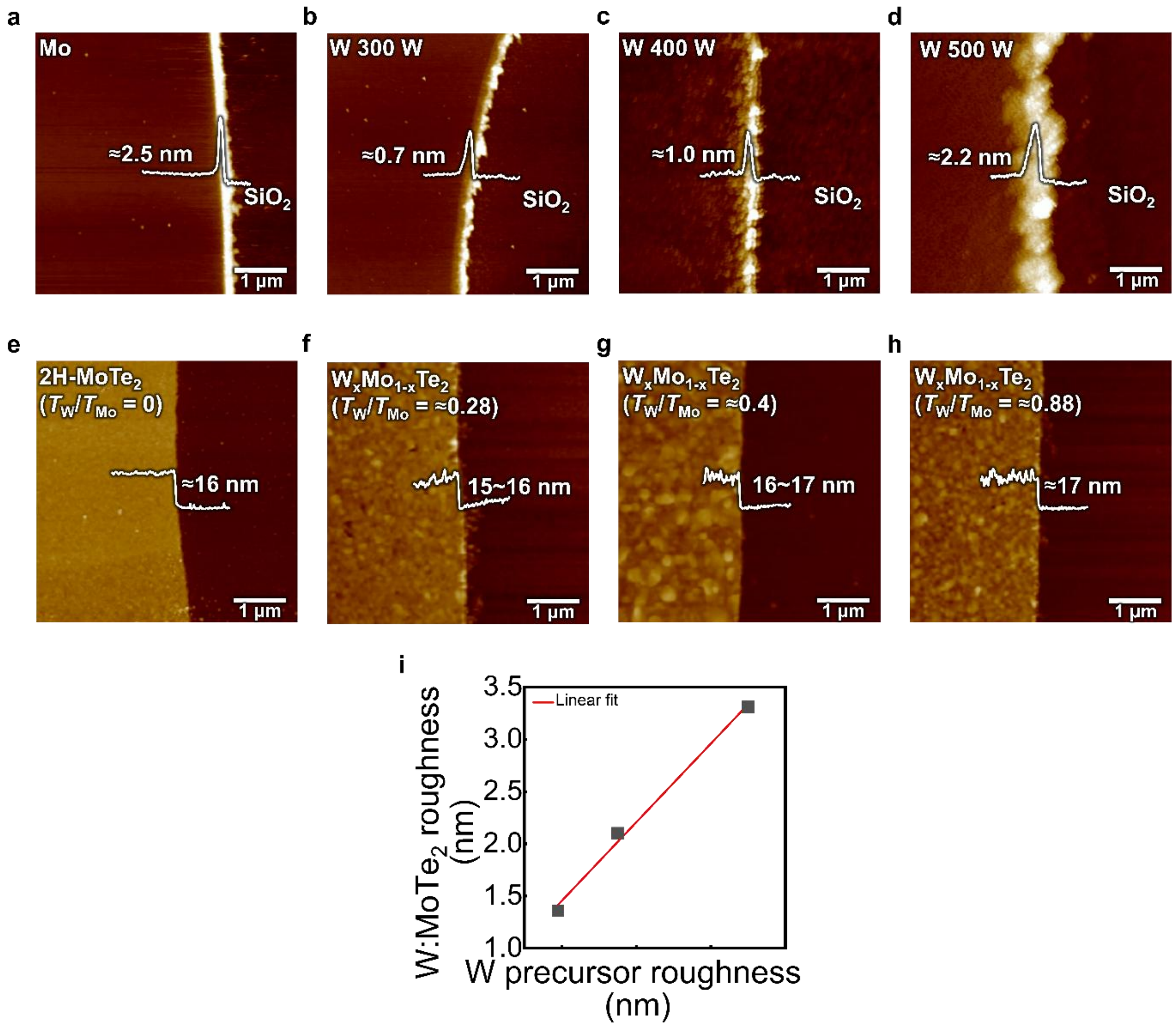

Figure S8. Modulating sputtering power for tungsten (W) to control the composition of $W_xMo_{1-x}Te_2$. (**a-d**) Atomic force microscopy (AFM) images showing the thickness of Mo and W precursor films deposited on a SiO_2 substrate using a DC sputtering system. (**a**) AFM image of Mo precursor film with thickness of ~2.5 nm. (**b-d**) AFM images of W precursor films with varying thicknesses, deposited at different sputtering powers: (**b**) 300 W, (**c**) 400 W, (**d**) 500 W, all with the same sputtering time (t = 1 sec). (**e-h**) AFM images of synthesized $MoTe_2$ and $W_xMo_{1-x}Te_2$ films, based on the Mo and W/Mo precursors with different sputtering power for W (300-500 W), while maintaining a constant Mo thickness. (**e**) AFM images of $MoTe_2$, showing the thickness corresponding to the Mo precursor after tellurization. (**f-h**) AFM images of $W_xMo_{1-x}Te_2$ with W precursors, corresponding to the different deposition powers shown in (b-d), with a fixed Mo thickness after tellurization. In this study, the composition of $W_xMo_{1-x}Te_2$ was controlled by adjusting the W/Mo thickness ratio (T_W/T_{Mo}). This was achieved by varying the W thicknesses while keeping the Mo thickness constant. (**i**) Plot showing the correlation between the roughness of the synthesized W:$MoTe_2$ and that of the pre-deposited W precursor. The variation originates from that of the pre-deposited W precursor, considering that the Mo precursor remains identical across all compositional variations of W:$MoTe_2$.

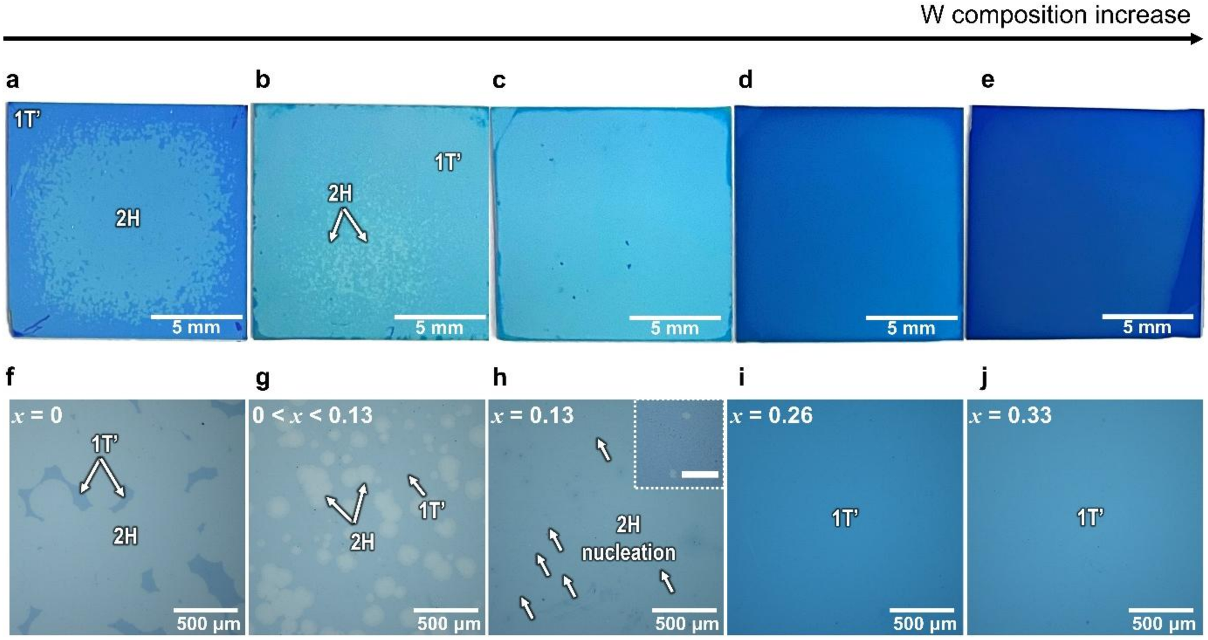

Figure S9. $W_xMo_{1-x}Te_2$ grown with different compositions (0 < *x* < 1). (**a-e**) Optical images of $W_xMo_{1-x}Te_2$ thin films grown under the same conditions (*T* = 700 °C, *t* = 120 min). (**f-j**) OM images of corresponding $W_xMo_{1-x}Te_2$ thin films in (a-e). (**a,f**) Film with *x* < 0.13, showing a predominance of the 2H structure, (**b,g**) film with *x* < 0.13, similar to (a), showing the 2H structures, (**c,h**) film with x ≈ 0.13, where 2H nucleation becomes nearly impossible, with 2H coverage falling below 0.2%, (**d,i**) film with *x* > 0.13, showing a uniform 1T' structure, and (**e,j**) film with *x* > 0.13, similar to (d), showing the 1T' structure.

For $W_xMo_{1-x}Te_2$ with a small *x* (< 0.13), the growth of the 2H structure is possible, as observed in both the optical and OM images (**Figures S9a,b,f,g**). However, when *x* increases to 0.13, 2H nucleation becomes nearly impossible in a ~1 x 1 cm^2 thin film, as the 2H coverage falls below 0.2 % (**Figures S9c,h**). Therefore, a uniform 1T' structure forms when *x* > 0.13 (**Figures S9d,e,i,j**).

a

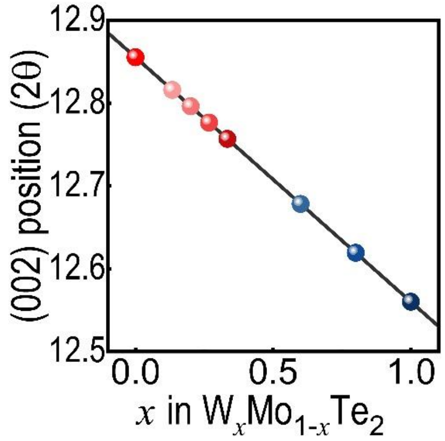

b

W composition (x)	(002) Peak position (2 theta)
0	12.8553
0.13	12.8159
0.20	12.7962
0.26	12.7765
0.33	12.7568
0.59	12.6783
0.79	12.6192
1	12.5601

Figure S10. Composition analysis of $W_xMo_{1-x}Te_2$ using Vegard's law. (**a**) Plot illustrating the correlation between the (002) peak positions in XRD patterns and various compositions (x) in $W_xMo_{1-x}Te_2$. The linear relationship demonstrates a strong fit with Vegard's law. (**b**) Table displaying the (002) peak positions depending on different x values.

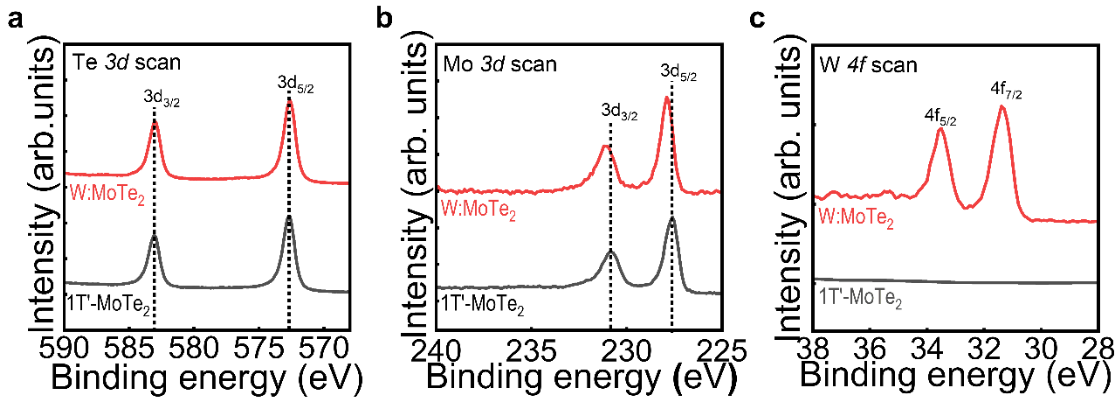

Figure S11. X-ray photoelectron spectroscopy (XPS) spectra of W:$MoTe_2$ and 1T'-$MoTe_2$. (**a**) Te *3d*, (**b**) Mo *3d*, and (**c**) W *4f* core-level spectra.

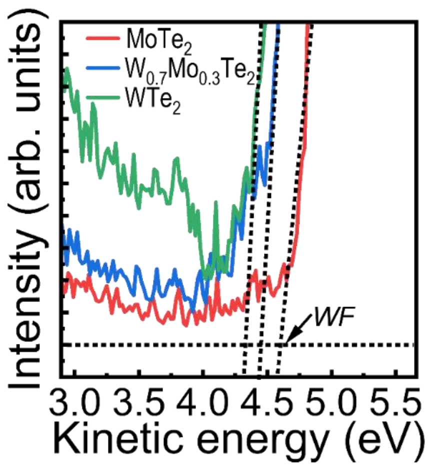

Figure S12. Ultraviolet photoelectron spectroscopy (UPS) spectra of metallic $MoTe_2$, $W_{0.7}Mo_{0.3}Te_2$, and WTe_2.

XPS analysis revealed W-Te bonding (31.39 eV and 33.54 eV for W $4f_{7/2}$ and W $4f_{5/2}$, respectively) (**Figure S11**), supporting the substitution of W atoms at Mo sites. In addition, the Mo 3*d* peaks of W:$MoTe_2$ shifted by 0.25 eV compared to those of 1T'-$MoTe_2$, reflecting an upward shift of the Fermi level.

Additionally, we carried out UPS analysis of 1T'-$MoTe_2$, T_d-$W_{0.7}Mo_{0.3}Te_2$, and T_d-WTe_2 to verify the range of work function modulation achievable by varying the W composition (**Figure S12).** The work function of WTe_2 is ≈4.44 eV, 0.11 eV lower than that of $MoTe_2$ (≈4.51 eV), and the $W_{0.7}Mo_{0.3}Te_2$ exhibits a similarly low value of ≈4.45 eV.

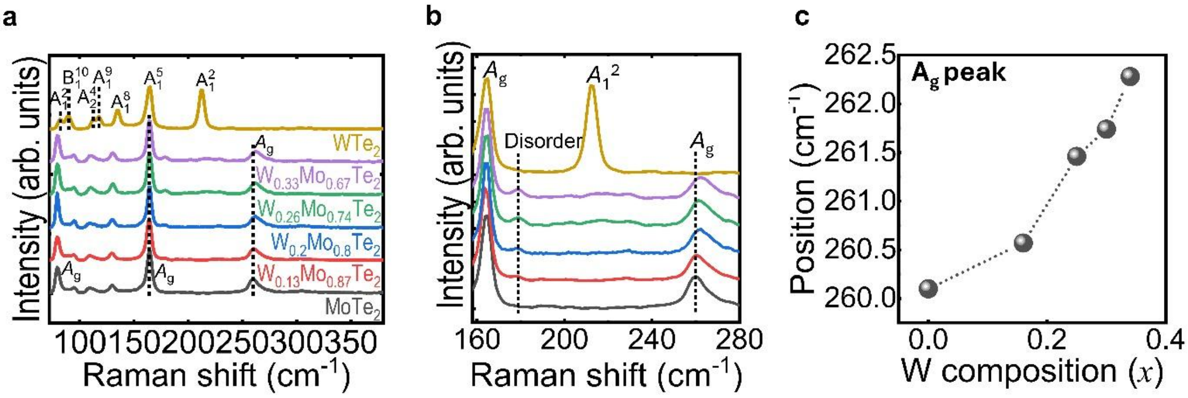

Figure S13. Composition-dependent Raman frequencies of 1T'-$W_xMo_{1-x}Te_2$. (**a**) Raman spectra of $W_xMo_{1-x}Te_2$, with intensity normalized to the A_g peak (163 cm^{-1}), ranging from 70 to 380 cm^{-1}. (**b**) Magnified Raman spectra from (a) in the range of 160 to 280 cm^{-1}. A disorder-related peak (~178 cm^{-1}), caused by the loss of translation symmetry in the 1T' phase,[17] is observed in the compositional range from x = 0.13 to 0.33. The peak intensity is maximum at x = 0.33 and disappears at x = 1.[17] (**c**) Plot showing the A_g peak (260 cm^{-1}) versus W composition (x). A blue-shift tendency is observed as the W content increases, consistent with previous reports on W/Mo ternary TMDs.[18,19] The blue shift with increasing W composition is attributed to the stronger bonding strength of W-Te compared with Mo-Te, which suppresses atomic vibration.[20,21]

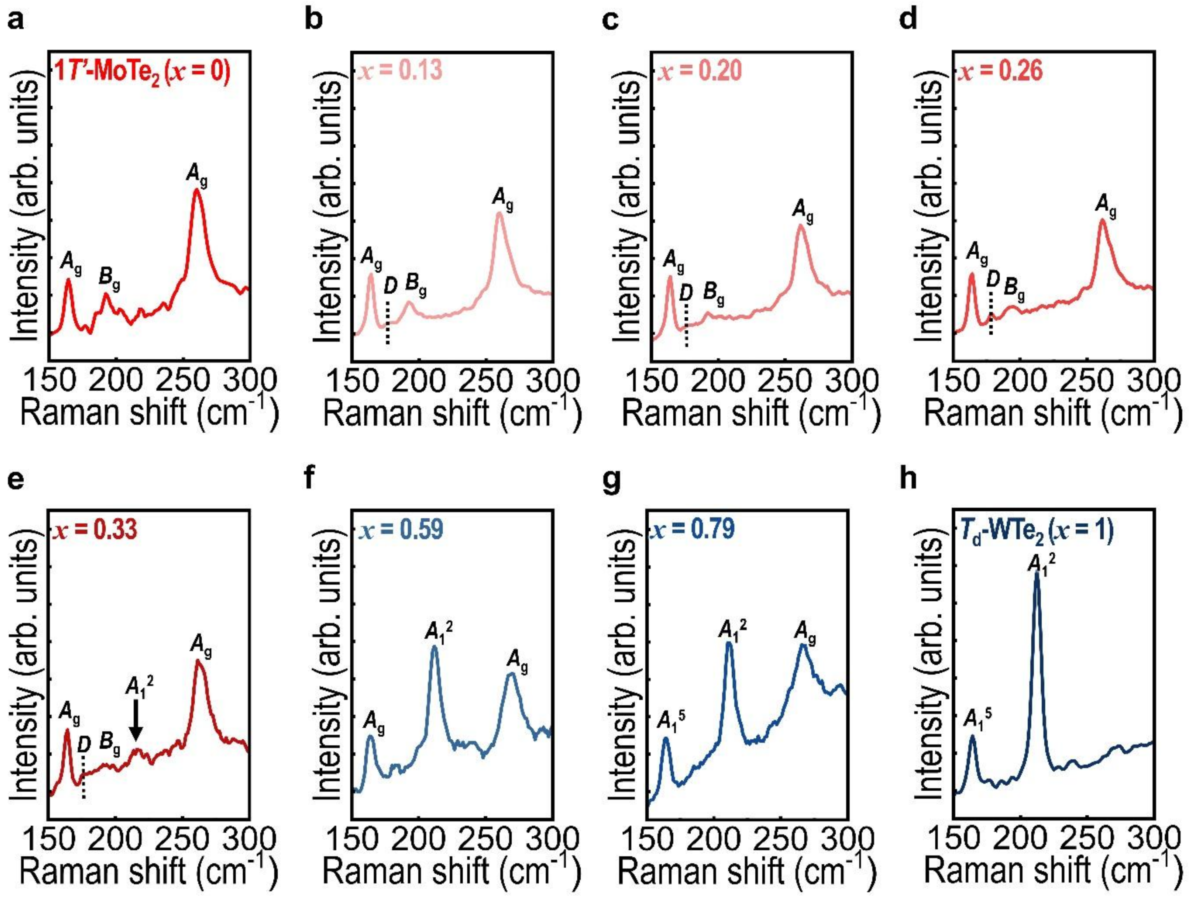

Figure S14. Phase transition from 1T' to T_d in $W_xMo_{1-x}Te_2$, characterized by Raman spectroscopy. (**a-h**) Raman spectra of $W_xMo_{1-x}Te_2$ with different W compositions; x = (**a**) 0, (**b**) 0.13, (**c**) 0.20, (**d**) 0.26, (**e**) 0.33, (**f**) 0.59, (**g**) 0.79, and (**h**) 1. The peak intensity is normalized to the intensity of the A_g peak (163 cm^{-1}). A peak labeled as "*D*" is a disorder-related peak that arises from the loss of translational symmetry in the 1T' phase.[17]

In the range of $0 \leq x \leq 0.33$, $W_xMo_{1-x}Te_2$ exhibits a 1T' structure, characterized by a dominant B_g signal from 1T'-$MoTe_2$ at 192 cm^{-1} (**Figures S14a-d**). However, as the W content increases ($x > 0.33$), the intensity of the B_g peak decreases, and the A_1^2 vibrational mode (212 cm^{-1}) from T_d-WTe_2 emerges (**Figure S14e**). In the range of $0.59 \leq x \leq 1$, the spectra display the dominant A_1^2 peak of T_d-WTe_2 (**Figures S14f-h**).

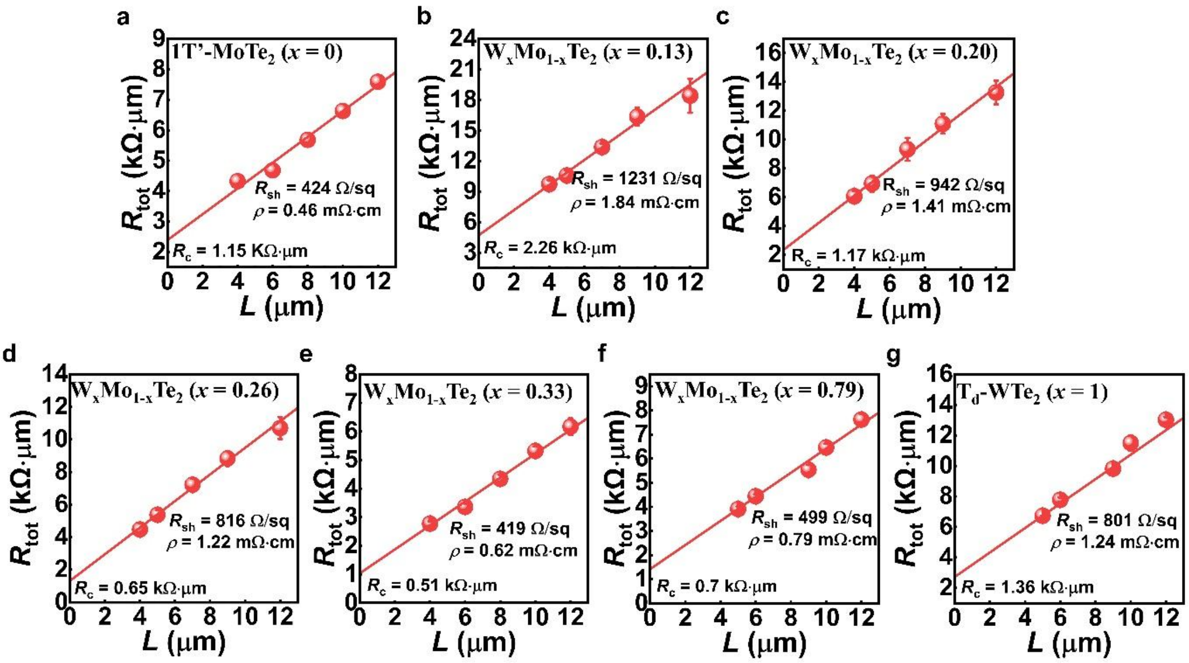

Figure S15. Extraction of sheet resistance of $W_xMo_{1-x}Te_2$ using the transfer length method (TLM). (**a-g**) TLM plots of $W_xMo_{1-x}Te_2$ with different compositions: (**a**) x = 0, (**b**) x = 0.13, (**c**) x = 0.20, (**d**) x = 0.26, (**e**) x = 0.33, (**f**) x = 0.79, and (**g**) x = 1. The slope of each plot indicates the sheet resistance (R_{sh}) of $W_xMo_{1-x}Te_2$.

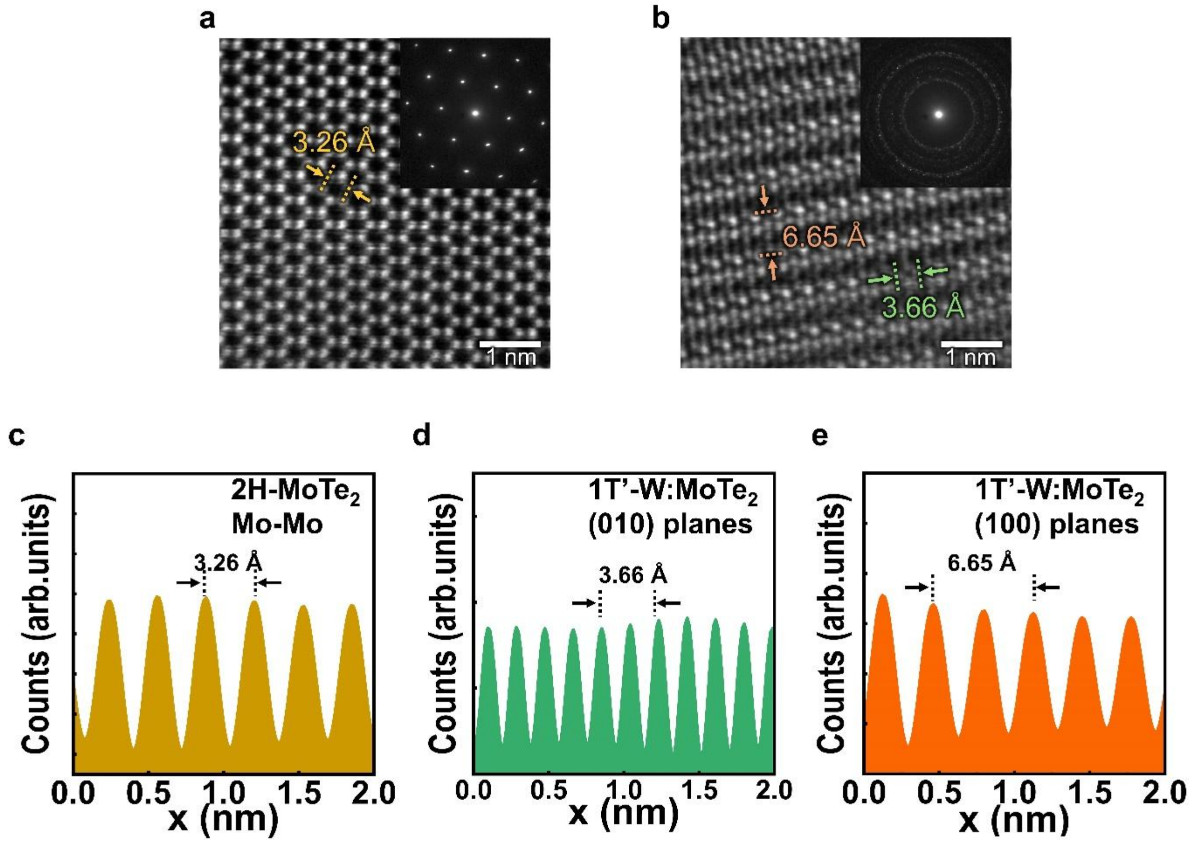

Figure S16. Transmission electron microscopy (TEM) structural analysis of 2H-$MoTe_2$ and 1T'-$W_xMo_{1-x}Te_2$. (**a**,**b**) Atomic-resolution STEM images for the regions of (**a**) 2H-$MoTe_2$ and (**b**) 1T'-$W_xMo_{1-x}Te_2$. The insets display the selected area electron diffraction (SAED) patterns for each structure. (**c-e**) *z*-intensity profiles measuring the Mo-Mo lattice distance of the (**c**) 2H phase, and interplanar spacings of (**d**) (010) planes and (**e**) (100) planes. The lattice spacings of synthesized 2H-$MoTe_2$ and 1T'-W:$MoTe_2$ are similar to those in previous studies on 2H- and 1T'-$MoTe_2$.[7,12,18] The slightly larger lattice spacings of W:$MoTe_2$ may originate from the insertion of W atoms into the 1T'-$MoTe_2$ matrix, which is consistent with the XRD *d*-spacing changes shown in Figure S10.

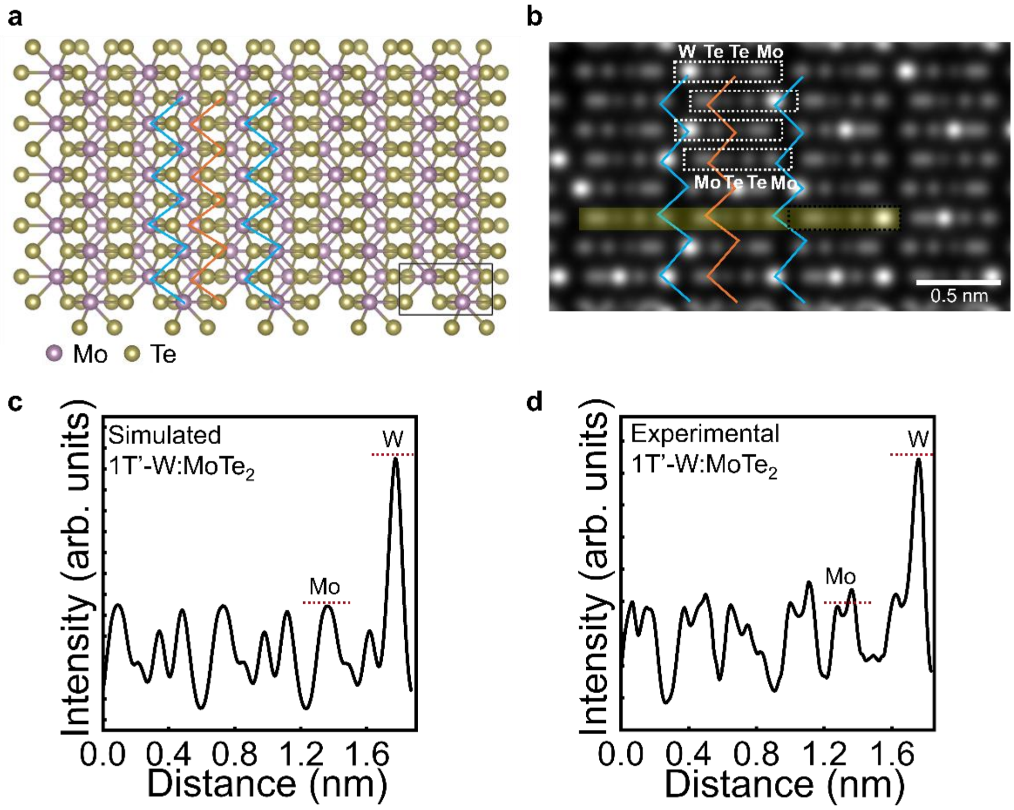

Figure S17. STEM analysis of W-alloyed $MoTe_2$. (**a**) Schematic of atomic structure of 1T'-phase $MoTe_2$. (**b**) Simulated HAADF-STEM image of bilayer W $MoTe_2$, where W atoms are randomly substituted at Mo sites. (**c**) Intensity profile of simulated bilayer W:$MoTe_2$ at the highlighted region in (**b**). (**d**) Intensity profile of the HAADF-STEM image of experimentally obtained thin 1T'-W:$MoTe_2$ film (< 3 nm thickness) shown in **Figure 3d**. The red dashed lines indicate similar intensity difference between the simulated and experimental results.

To identify W substitution, we confirmed the presence of W by comparing the HAADF intensity profiles (**Figure S17**). Because atomically identifying the substitutions in thick films is challenging (**Figure S16b**), we intentionally synthesized a thinner structure (< 3 nm) to verify W substitution (**Figure 3d**). The HAADF intensity of a substituted W atom in the simulated image resembles that observed in experimental results. The substitution of W elements is also confirmed by XRD measurements (**Figures 2b** and **S10**).

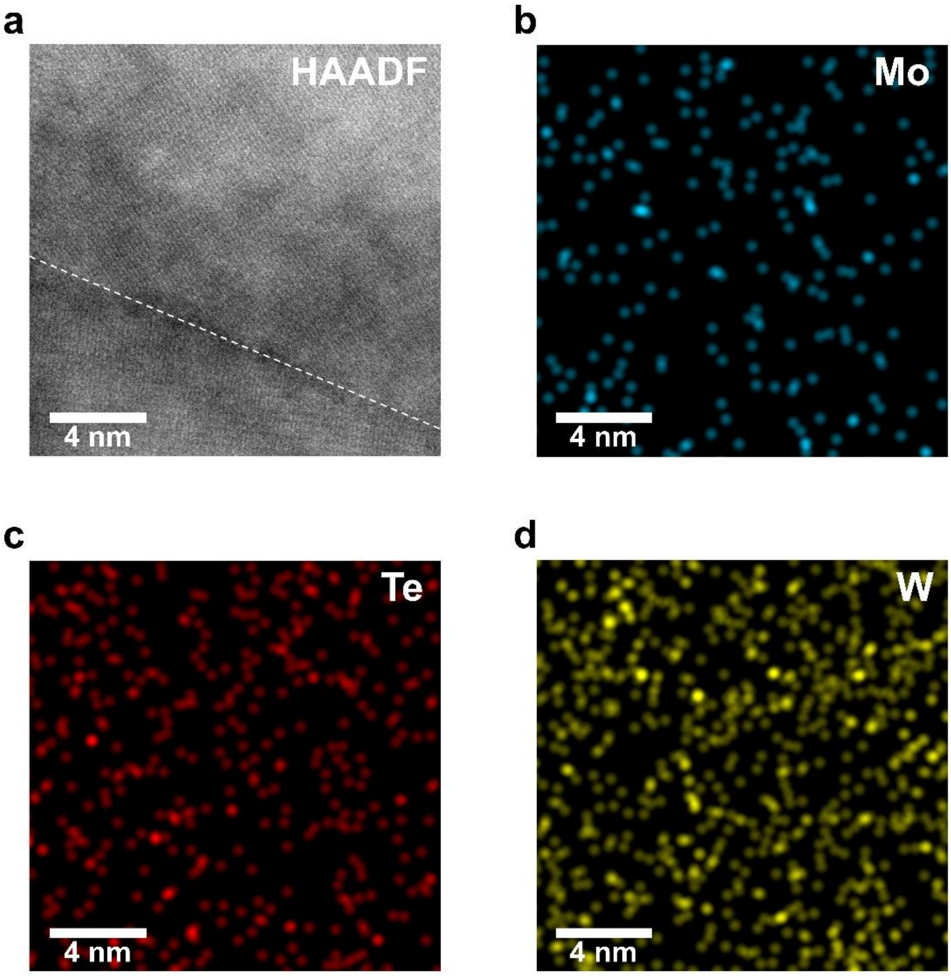

Figure S18. TEM-EDS elemental analysis of $W_xMo_{1-x}Te_2$. (**a**) Low magnification HAADF-STEM image of the $W_xMo_{1-x}Te_2$ thin film. The dashed line indicates the grain boundary of differently oriented $W_xMo_{1-x}Te_2$ grains. (**b-d**) Corresponding EDS mapping images showing the distribution of elements in the sample: (**b**) Mo atoms, (**c**) Te atoms, and (**d**) W atoms. These images indicate a uniform distribution of the elements across the $W_xMo_{1-x}Te_2$ thin film.

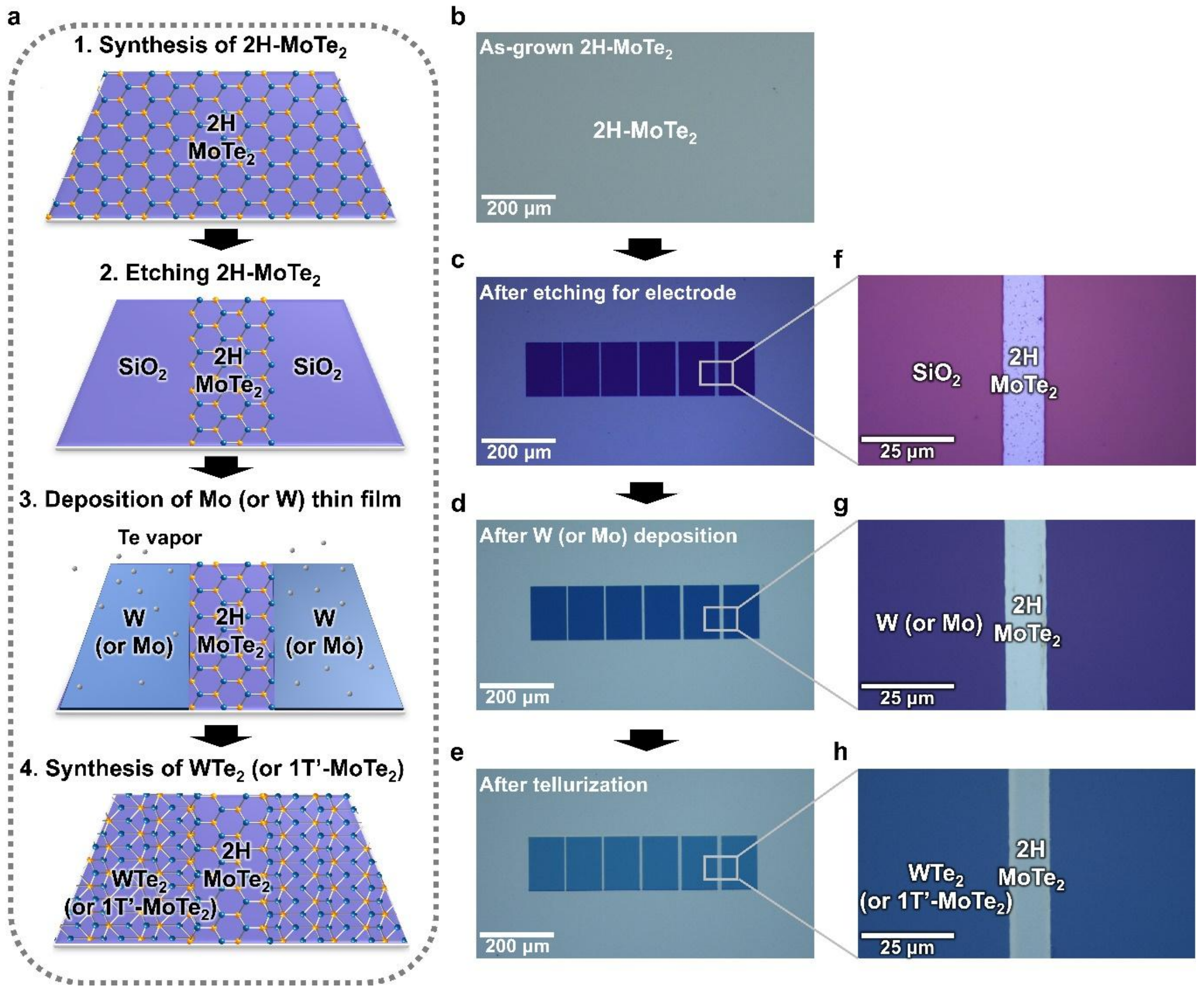

Figure S19. Two-step fabrication processes for T_d-WTe_2 or 1T'-$MoTe_2$ edge contacts. (**a**) Schematic illustrations of the fabrication processes. (**b-e**) OM images corresponding to each step shown in (a); (**b**) as-grown 2H-$MoTe_2$ for the channel, (**c**) after etching of 2H-$MoTe_2$, (**d**) after deposition of W (or Mo) precursor patterns, and (**e**) after additional tellurization of W (or Mo) precursor to convert precursors to WTe_2 (or 1T'-$MoTe_2$) at T = 500 °C. (**f-h**) Zoomed-in OM images captured at the square box in (c-e), respectively.

In **Figure S19**, edge-contact MSJ FETs with 2D T_d-WTe_2 or 1T'-$MoTe_2$ are produced using a two-step synthetic method, similar to previous reports.[5,6,22] The fabrication process begins with standard photolithography to define the patterns. The 2H-$MoTe_2$ channel region is then isolated using reactive ion etching (RIE) (**Figures S19c,f**). Next, W (or Mo) precursor films are deposited (**Figures S19d,g**). Finally, the precursors are converted to T_d-WTe_2 (or 1T'-$MoTe_2$) through a tellurization process at a growth temperature of 500 °C (**Figures S19e,h**). It should be noted that a one-step synthetic metallization for WTe_2 or $MoTe_2$ cannot be applied due to material degradation and phase impurity issues (**Figures S20a,d** and **S9**).

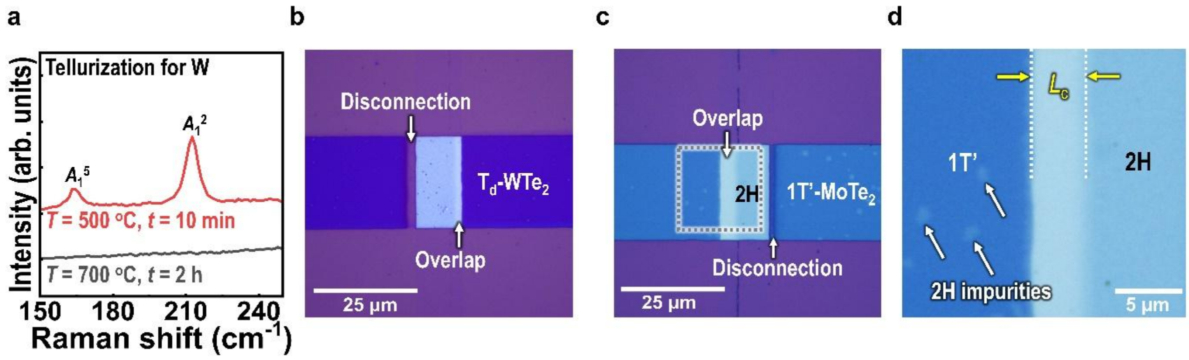

Figure S20. Disadvantages of the two-step metallization process for edge-contact 2D MSJ FETs. (**a,b**) Challenges in realizing the edge-contact FET with T_d-WTe_2. (**a**) Raman spectra of W thin film after tellurization under different tellurization conditions. The black spectrum corresponds to the result from the tellurization under the same condition as for the full growth of 2H-$MoTe_2$ (T = 700 °C, t = 2 h). The red spectrum is obtained after tellurization at a relatively low temperature for synthesizing T_d-WTe_2 (T = 500 °C, t = 10 min).[23] (**b**) OM image of $MoTe_2$-WTe_2 edge-contact FET, showing misaligned region. (**c,d**) Challenges in producing 1T'-$MoTe_2$ edge contacts. (**c**) OM image of 2H-1T' $MoTe_2$ edge-contact FET. (**d**) Enlarged image of the area within the dashed box in (c). Due to the overlapping region between 1T' and 2H-$MoTe_2$, the contact length (L_c) becomes longer than pure edge contact (i.e., thickness of the junction).

Figure S20 depicts the challenges of two-step metallization process for creating edge contacts using T_d-WTe_2 and 1T'-$MoTe_2$. **Figure S20a** shows that tungsten (W) thin films are decomposed under the synthetic conditions for fully growing 2H-$MoTe_2$ (T = 700 °C, t = 2 h). Due to a narrow growth window, WTe_2 edge-contact FETs must be constructed using a two-step synthetic method. This process involves more fabrication steps to first etch the 2H-$MoTe_2$ region and then deposit W in that region. For pure edge contact without vertically overlapping junctions, the edge plane of the deposited W thin film must come in a contact with the etched region of 2H-$MoTe_2$. These intricate photolithography processes can cause misalignment issues, such as disconnection and overlap, within arrays on a chip (**Figure S20b**).

As for the 1T'-$MoTe_2$ electrode, tellurization of Mo thin film at a high temperature of 700 °C leads to random 2H nucleation, making selective-area phase control difficult (**Figures S9a,f**). Thus, 1T'-$MoTe_2$ edge contact must also be created using two-step synthetic method. Similar to WTe_2 edge-contact FETs, this elaborates that photolithography process can lead to misalignment issue, such as disconnection and overlap (**Figure S20c**). The enlarged image in the box in (c) shows the overlapped region of 1T'/2H-$MoTe_2$ (**Figure S20d**). The presence of this overlapped region indicates that it is not a pure edge contact with a longer L_c. Unlike T_d-

WTe_2, the small formation energy difference (≈35 meV) between 1T' and 2H results in 2H impurities in the electrode area[24] (**Figure S20d**).

Additionally, the two-step synthetic method introduces oxidation-related defects. During the W (or Mo) thin film deposition process, the etched region of 2H-$MoTe_2$ is inevitably exposed to air. Due to the vulnerability of the edge area to oxidation,[25] the etched region is likely to be damaged during the metallization process. Oxidation at the edge region is a major drawback in manufacturing edge-contact FET using lithographic processes.[26]

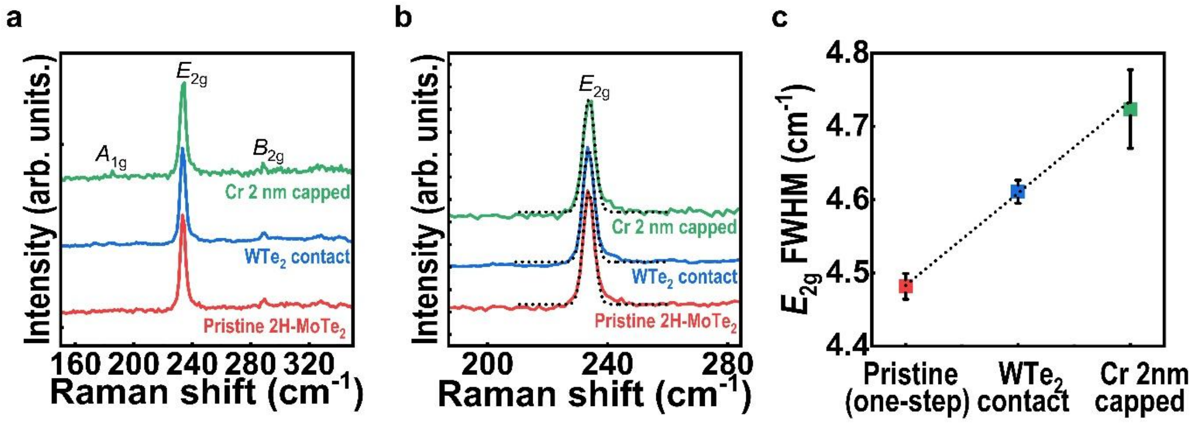

Figure S21. Raman spectroscopy analysis to investigate channel quality depending on metallization processes. (**a**) Raman spectra of pristine (red), WTe_2-contacted (blue), and Cr-deposited (green) 2H-$MoTe_2$, shown in the range from 150 to 350 cm^{-1}. (**b**) Raman spectra in a narrow range to compare the E_{2g} peak broadening at a glance. The full width half maximum (FWHM) of E_{2g} peaks was extracted by Gaussian fitting (dotted lines). Plot of the FWHM of E_{2g} Raman peaks of 2H-$MoTe_2$ before/after metallization process. The Raman spectrum of "pristine 2H-$MoTe_2$" was measured for the sample prepared in MSJ with W:$MoTe_2$ edge contacts.

In this study, we compare the FWHM values of E_{2g} Raman peaks to investigate the channel qualities based on different metallization processes (**Figure S21**). The FWHM can provide evidence to evaluate channel quality, with less peak broadening indicating higher quality and lower defect density in the 2D channel.[27] The measured samples are; (i) 2H-$MoTe_2$ prepared by one-step metallization for W:$MoTe_2$ edge contact (i.e., pristine $MoTe_2$), (ii) 2H-$MoTe_2$ in two-step edge-contact $MoTe_2$-WTe_2 MSJ, and (iii) 2H-$MoTe_2$ with 2 nm of Cr deposited via e-beam evaporation.

We find that the offset of FWHM values between pristine $MoTe_2$ and Cr-capped $MoTe_2$ is ~0.24 cm^{-1}. However, the $MoTe_2$ with WTe_2 edge contact shows less broadening (< 0.12 cm^{-1}) than that the Cr-capped crystal. On the other hand, the W:$MoTe_2$ edge contact was fabricated by one-step synthetic method, where the channel and contact were synthesized simultaneously. This results in the FWHM of pristine $MoTe_2$ being equivalent to that of the $MoTe_2$ processed with the one-step process. Thus, our one-step synthetic method has the advantage of preserving channel quality without introducing defects.

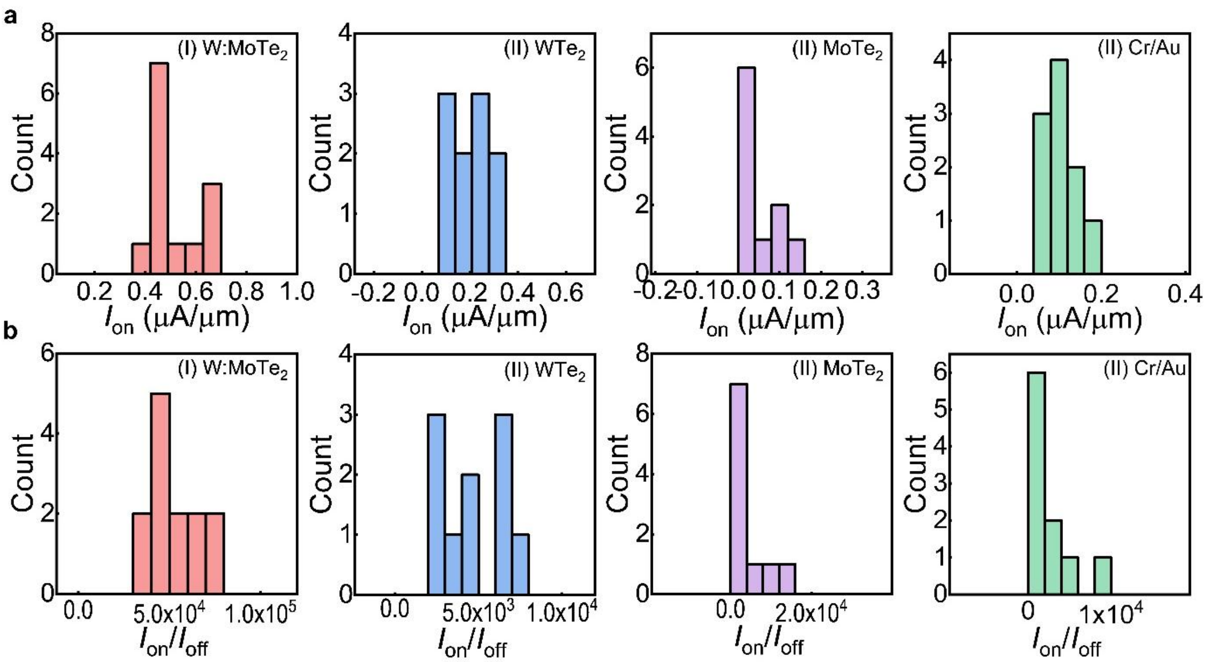

Figure S22. Statistical analysis of electrical characteristics of various $MoTe_2$ FETs with different contacts. (**a,b**) Histograms showing the distribution of (**a**) the on-state current density (I_{on}), and (**b**) on-to-off current ratio (I_{on}/I_{off}) of representative devices with W:$MoTe_2$, WTe_2, $MoTe_2$ and Cr/Au contacts. Numerical devices including 13 devices with W:$MoTe_2$ contacts and each 10 devices with other contacts were measured at V_{ds} = -1 V. All transistors have channel length (L) of 11 μm. Each transfer curve is labeled on the right-top side with different contacts: "(I) W:$MoTe_2$", "(II) WTe_2", "(II) $MoTe_2$" and "(II) Cr/Au".

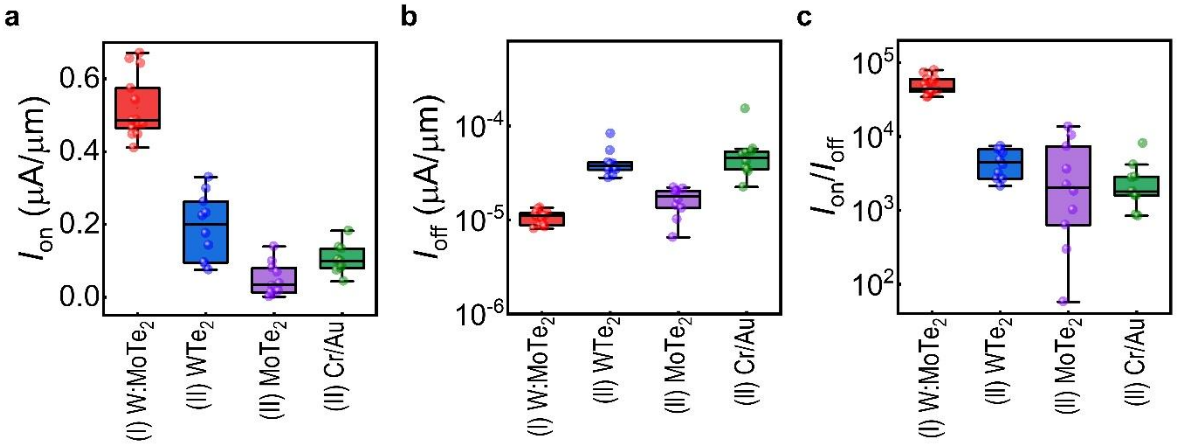

Figure S23. Comparison of on-state current (I_{on}), off-state current (I_{off}) and on-to-off current ratio (I_{on}/I_{off}) in $MoTe_2$ FETs with different contacts. (**a**) Statistical plots of I_{on}, (**b**) I_{off} and (**c**) I_{on}/I_{off} of $MoTe_2$ FETs with different contacts. The values of the electrical properties were extracted from the transfer curves at V_{ds} = -1 V.

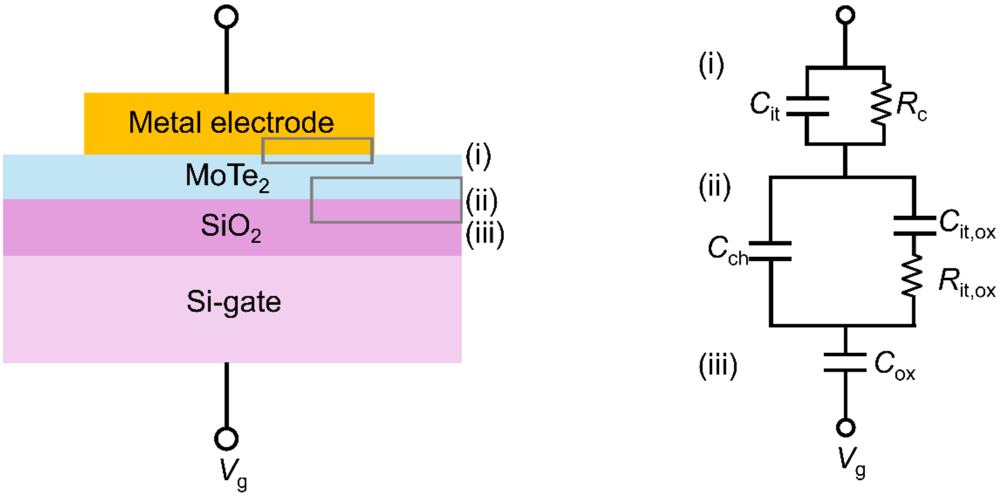

Figure S24. Schematic cross-sectional view of a transistor with MSIM structure and corresponding equivalent circuit model. Capacitance and resistance occur from (i) contact metal-semiconductor interface, (ii) semiconductor-gate dielectric interface, and (iii) gate dielectric.

Regarding the defect density at the W:$MoTe_2$-$MoTe_2$ edge-contact FET, the atomic resolution TEM images reveal a clean MSJ interface (**Figures 3c,f**), which contrasts with previous studies[12,28,29] that report the physical damage caused by direct metal deposition. This lower defect density can be estimated by calculating the interfacial trap density (D_{it}), as interfacial defect states significantly affect the switching behavior of the metal-semiconductor-insulator-metal (MSIM) structure (**Figure S24**), particularly the subthreshold swing (SS) values, as follows:[30-32]

$$SS = \frac{dV_g}{d(logI_{ds})} = ln10\frac{k_B T}{q}\left(\frac{C_{ox}+C_{ch}+C_{it,ox}+C_{it}}{C_{ox}}\right)$$

$$\approx 60\ mV/dec\left(1+\frac{C_{it,ox}+C_{it}}{C_{ox}}\right)$$

Here, the values of C_{ox}, C_{ch} and $C_{it,ox}$ are identical in our MSIM structures, therefore, the difference in SS values can be attributed solely to the contact properties. We found that the edge-contact FET exhibited a lower SS value (≈16.95 V/dec) compared with the top-contact FET (≈32.54 V/dec). Based on this, the total interface trap density ($D_{it,ox}$ + D_{it}) was calculated to be ≈2.02 × 10^{13} $cm^{-2}eV^{-1}$ for the W:$MoTe_2$ edge-contact FET, and ≈3.88 × 10^{13} $cm^{-2}eV^{-1}$ for the Cr/Au top-contact FET, consistent with our claim for lower defect density after the one-step method.

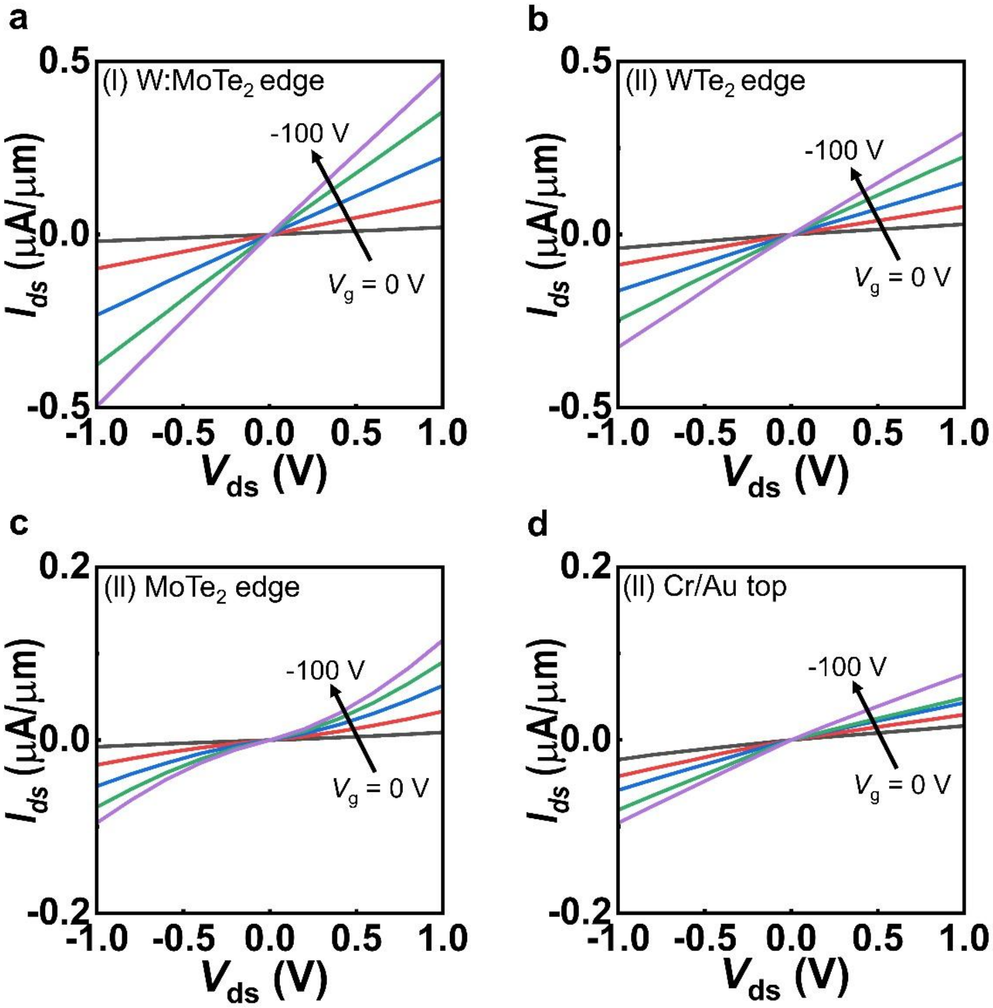

Figure S25. Extraction of nonlinearity (*N*) from the output characteristics. (**a-d**) Representative output characteristics showing the degree of linearity of FETs with different contacts; (**a**) (II) W:$MoTe_2$ edge contact, (**b**) (II) WTe_2 edge contact, (**c**) (II) $MoTe_2$ edge contact, and (**d**) (II) Cr/Au top contact. The linear behavior of the W:$MoTe_2$ edge-contact FET indicates nearly ohmic contact.

The nonlinearity (N), an indicator of Schottky or ohmic contact, is extracted from the output characteristics at certain drain voltage (V_{ds}) as below:[33]

$$N = \frac{d^2 I_{ds}/dV_{ds}^2}{2(dI_{ds}/dV_{ds})}$$

Compared with other contacts, the W:$MoTe_2$ edge contact shows linear *I-V* relationships, which can be attributed to the enhanced carrier tunneling probability derived by strong hybridization at the edge MSJ interface[34] (**Figures 3c,f** and **5a**).

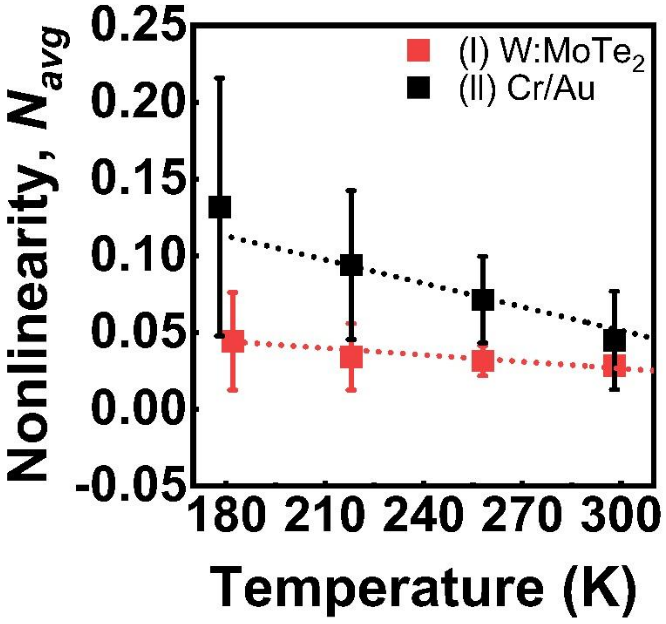

Figure S26. Nonlinearities of W:$MoTe_2$ edge and Cr/Au top contacts as a function of temperature. The elevated nonlinearity value observed at low temperatures for the Cr/Au top contact indicates the presence of a significant Schottky barrier.

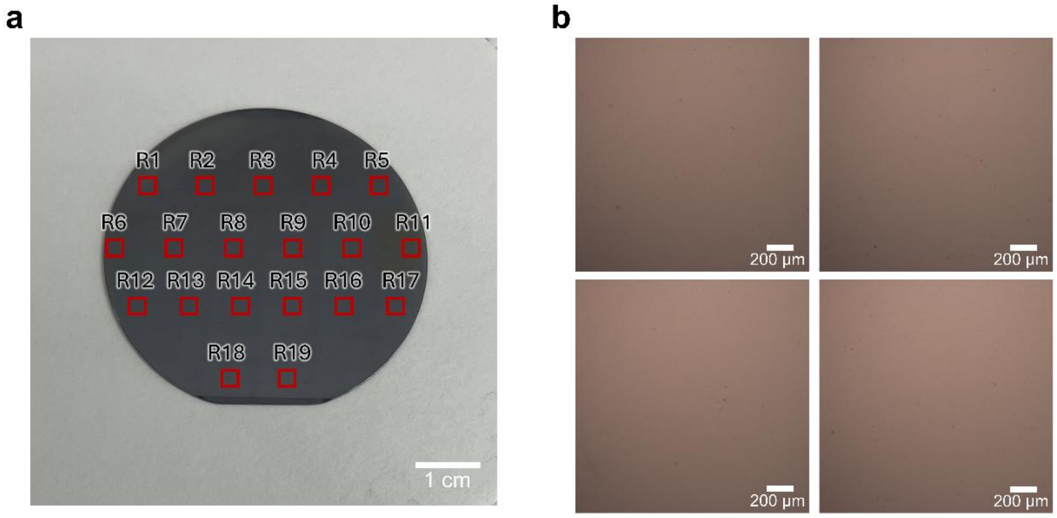

c

#	Square resistance (Ω/sq)	#	Square resistance (Ω/sq)
1	325	11	327
2	423	12	304
3	399	13	304
4	442	14	305
5	380	15	303
6	327	16	308
7	337	17	320
8	331	18	278
9	335	19	288
10	343		

Figure S27. Analysis of the sheet resistance of W:$MoTe_2$ thin film across a 2-inch wafer scale. (**a**) Photograph of a synthesized W:$MoTe_2$ thin film on a 2-inch sapphire substrate. (**b**) OM images randomly captured in (a). (**c**) Sheet resistance analysis conducted at 19 different locations across the area in (a).

To demonstrate uniformity of synthesized W:$MoTe_2$ thin film across the 2-inch wafer scale, we measured the room-temperature sheet resistance of a metallic 1T'-W:$MoTe_2$ thin film at 19 locations across a 2-inch wafer scale using a commercial 4-point probe system (CMT-SR2000N) (**Figure S27**). The measured resistance of the film was 335 ± 43 Ω/sq.

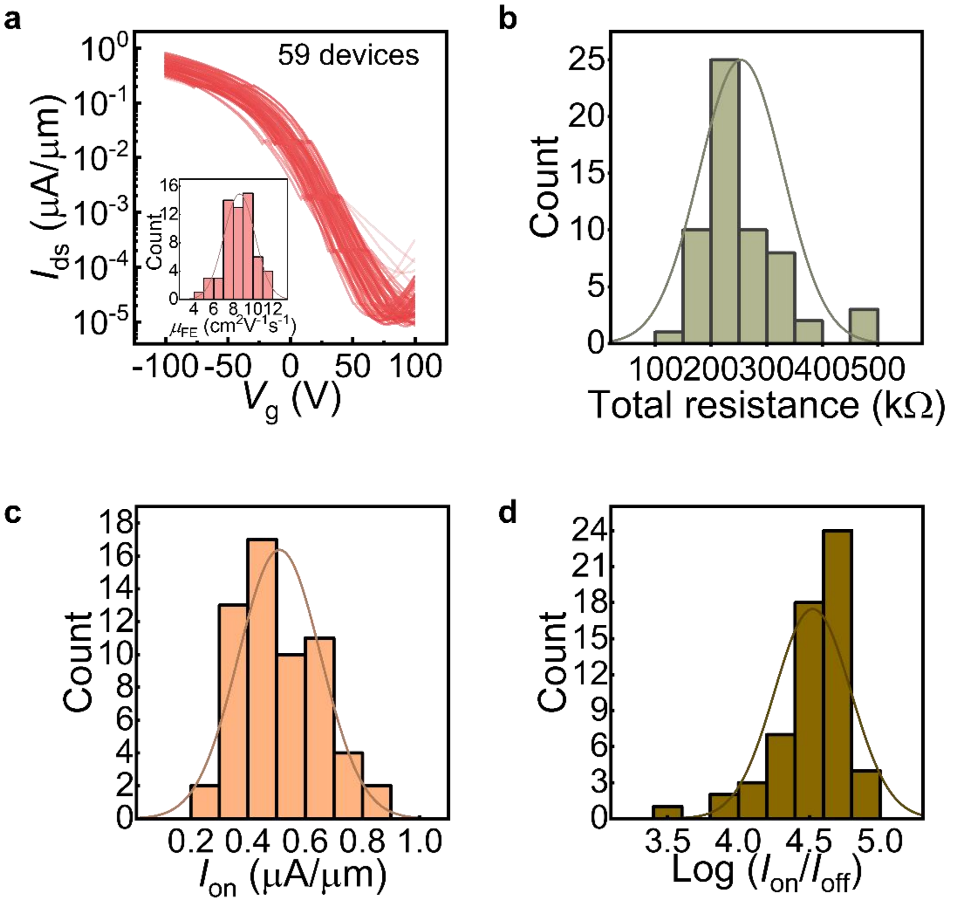

Figure S28. Uniformity of W:MoTe$_2$ edge-contact FETs arrays. (**a**) Transfer curves of 59 different FETs measured at V_{ds} = -1 V. The inset shows the field-effect mobility (μ_{FE}) distribution of 59 devices. (**b-d**) Corresponding histogram representing (**b**) the total resistance, (**c**) on-state current (I_{on}), and (**d**) on-to-off current ratio (I_{on}/I_{off}) values derived from (a).

We evaluated the uniformity of W:MoTe$_2$-MoTe$_2$ FETs distributed across the wafer (**Figure S28**). **Figure S28** presents the overlapped transfer curves of all 59 devices, together with the distributions of their electrical properties, including μ_{FE}, on-state total resistance, I_{on}, and I_{on}/I_{off}. Here, we calculated the total resistance according to an equation $R_{tot} = (V_{ds}/I_{ds})(W/L)$, where W and L are the channel width and length of the transistor, respectively. The on-state total resistance and I_{on} values were 254 ± 74 kΩ and 0.51 ± 0.14 μA/μm at V_g = -100 V, respectively. The extracted μ_{FE} and log(I_{on}/I_{off}) were 8.62 ± 1.56 cm^{2}V^{-1}s^{-1} and 4.52 ± 0.26, respectively.

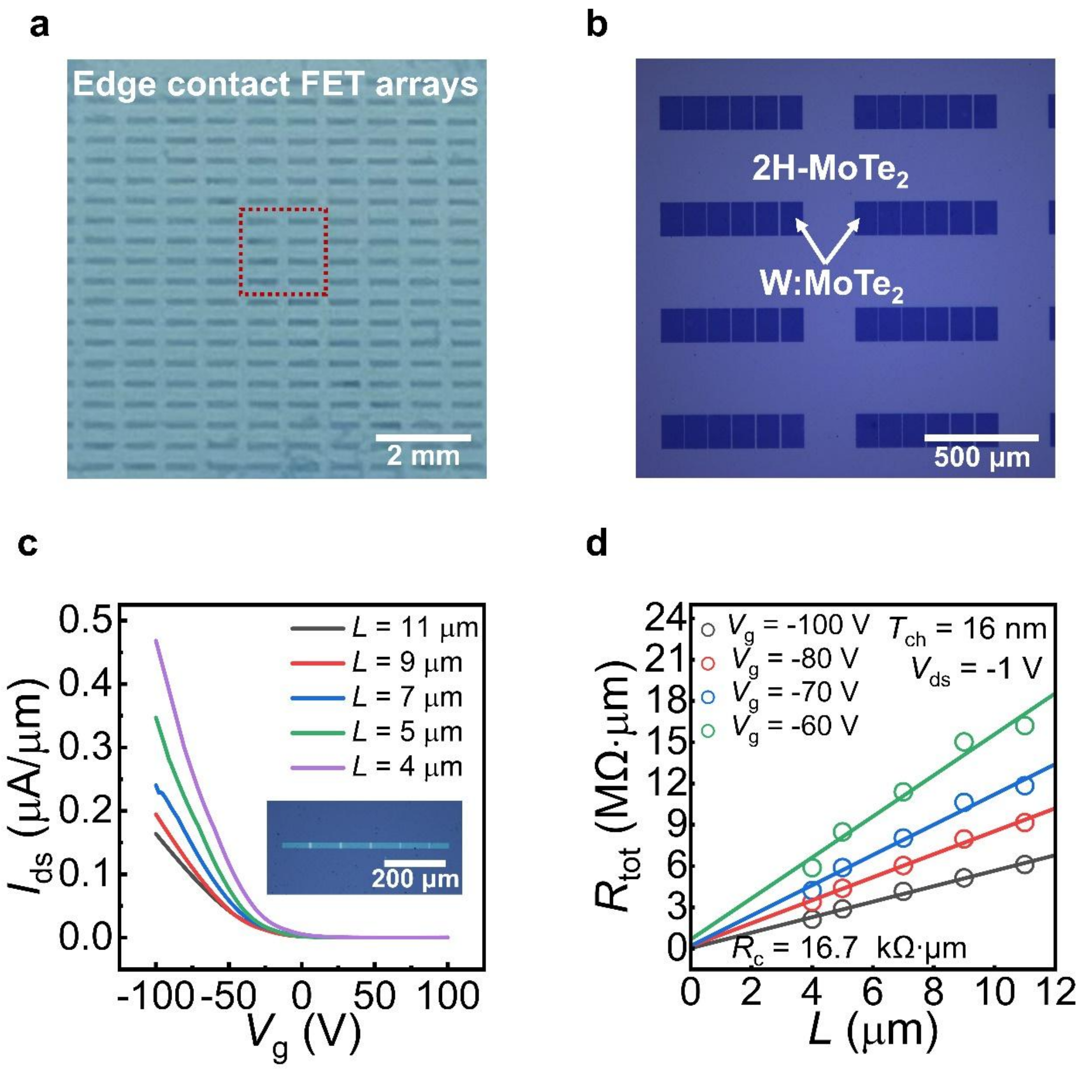

Figure S29. Contact resistance extraction of W:MoTe$_2$ edge-contact FET. (**a**) Optical image of the edge-contact FET arrays produced by one-step synthetic method. (**b**) Zoomed-in OM image of FET arrays captured within the red square box in (a). (**c**) Transfer curves of the edge-contact FETs with different channel length (L = 4-11 μm) at V_{ds} = -1 V. The inset shows the OM image of the TLM structure. (**d**) TLM plots of W:MoTe$_2$ edge-contact FET with different V_g.

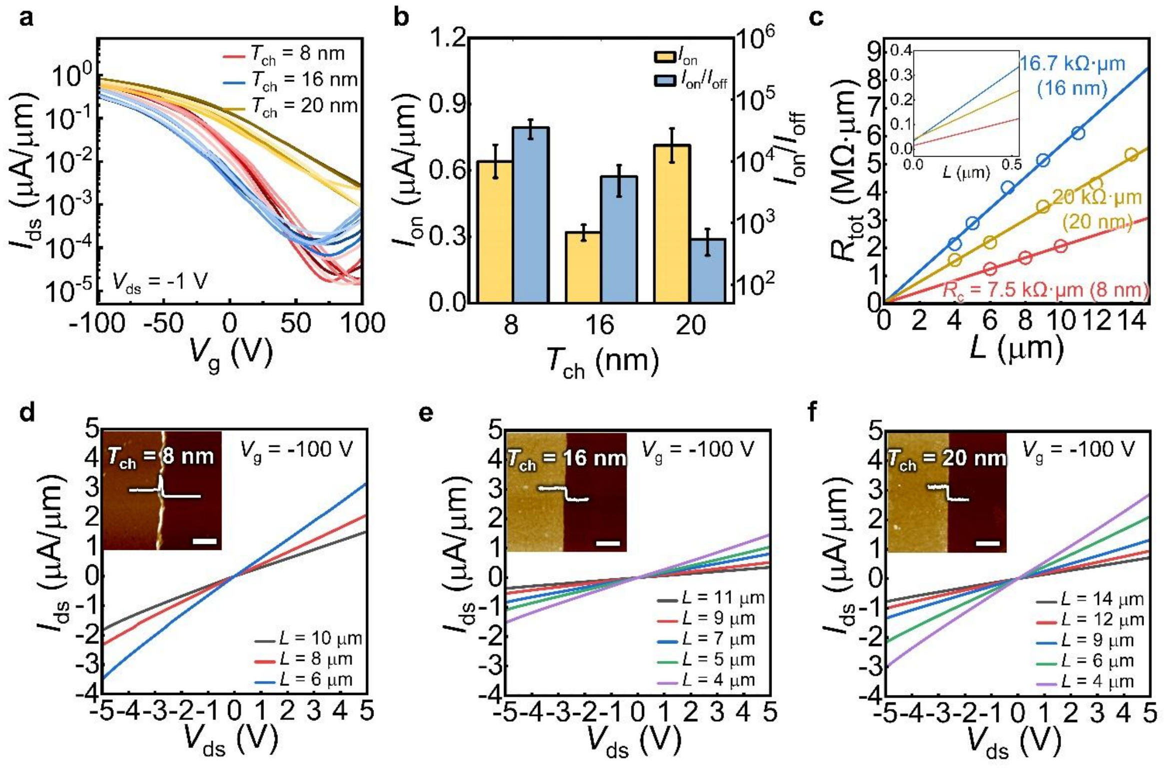

Figure S30. Electrical properties of edge-contact FETs with different channel thicknesses. (**a**) Transfer curves (I_{ds}-V_g) of W:$MoTe_2$ edge-contact FETs with different channel thickness (T_{ch} = 8, 16, and 20 nm). (**b**) Statistical plots showing the average values of I_{on} (yellow) and I_{on}/I_{off} (blue) obtained from the transfer curves in (a). (**c**) TLM plots of edge-contact FETs. We obtained R_c of 7.5, 16.7 and 20 kΩ·μm for T_{ch} of 8, 16, and 20 nm, respectively. (**d-f**) Output curves (I_{ds}-V_{ds}) of FETs with T_{ch} of (**d**) 8, (**e**) 16 nm, and (**f**) 20 nm, measured at -100 V. Insets are the representative AFM images indicating each T_{ch}.

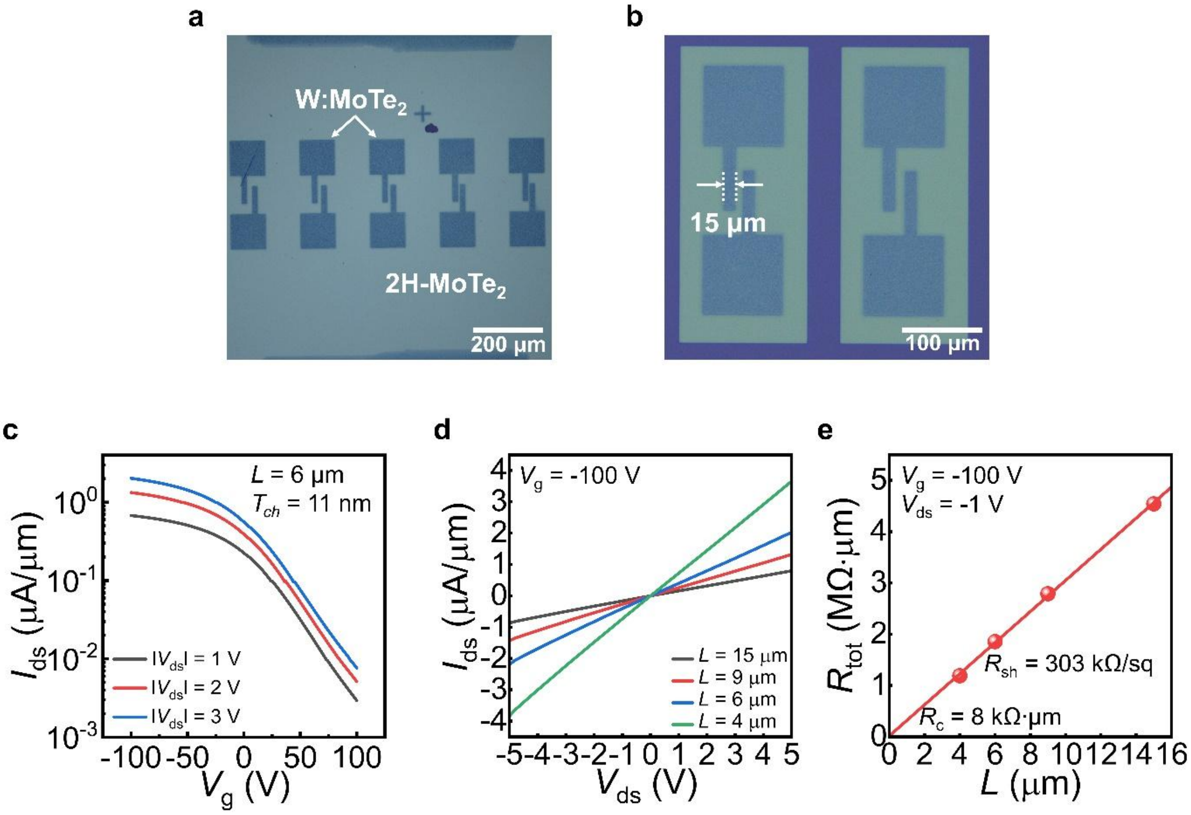

Figure S31. Electrical properties of edge-contact FETs with different contact dimensions and shapes compared to those in Fig. 5. (**a**,**b**) OM images of W:$MoTe_2$ edge-contact FETs with reduced size of contact pads, captured at (**a**) low magnification and (**b**) high magnification. (**c**) Representative transfer curves of the edge-contact FETs with $|V_{ds}|$ ranging from 1 to 3 V. The FET with L = 6 μm, T_{ch} = 11 nm was measured. (**d**) I_{ds}-V_{ds} plots with different L of 4-15 μm. (**e**) TLM plots showing the linear dependence of R_{tot} on L. The R_c was estimated to be 8 kΩ·μm at V_g = -100 V. This R_c value (8 kΩ·μm) is similar with that of 8-nm-thick-channel FET (7.5 kΩ·μm), indicating the electrical characteristic are independent of the contact pad sizes.

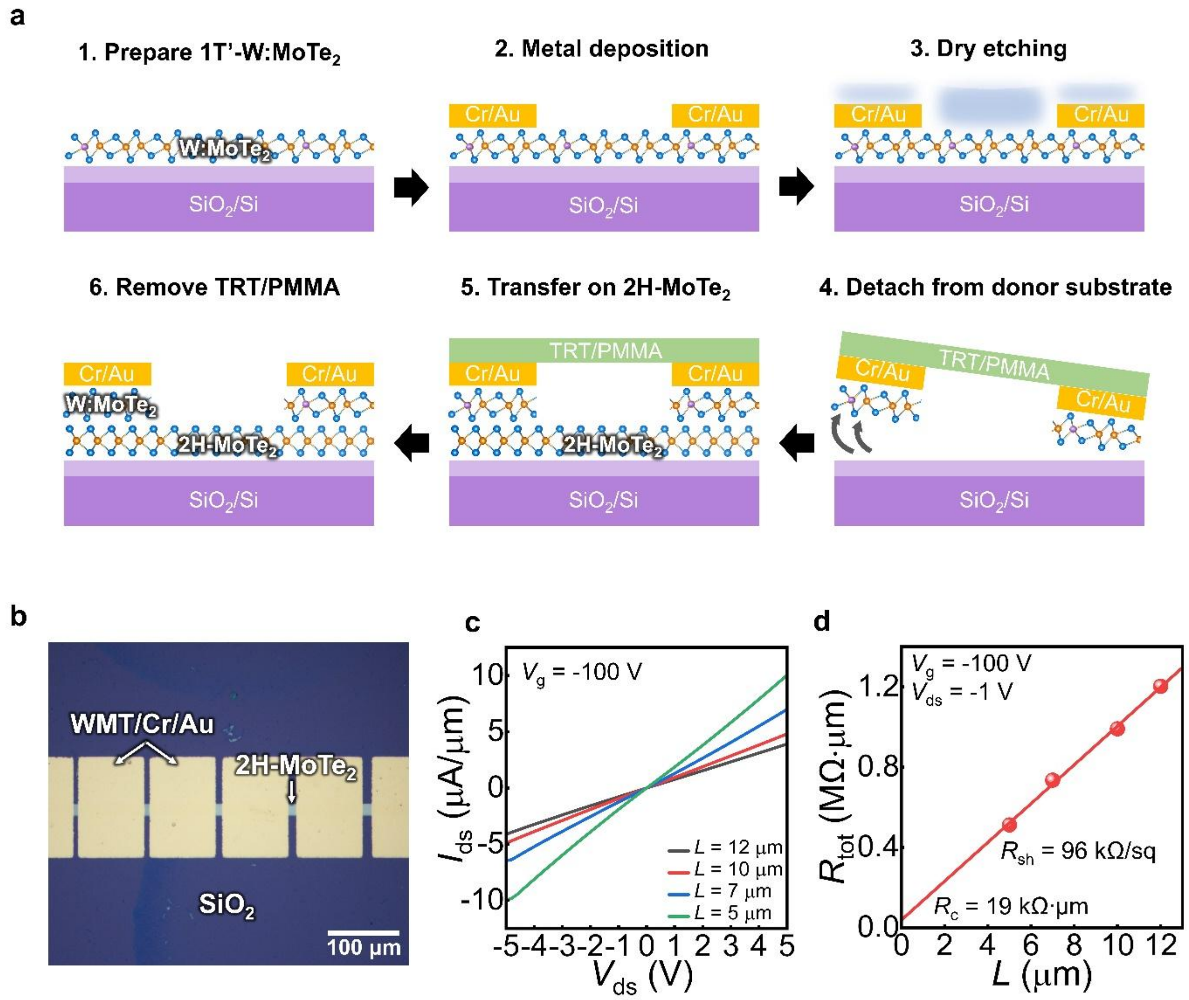

Figure S32. Electrical characterizations of W:MoTe$_2$ top-contact FETs. (**a**) Schematics demonstrating the processes for preparing the W:MoTe$_2$/MoTe$_2$ vertical heterostructure FET. (**b**) OM image of the assembled W:MoTe$_2$ top-contact FET with Cr/Au contact pad. (**c**) I_{ds}-V_{ds} output characteristics for different L at V_g = -100 V. (**d**) Corresponding TLM plots of the top-contact devices. The R_c was extracted as 19 kΩ·μm at V_g = -100 V and V_{ds} = -1V.

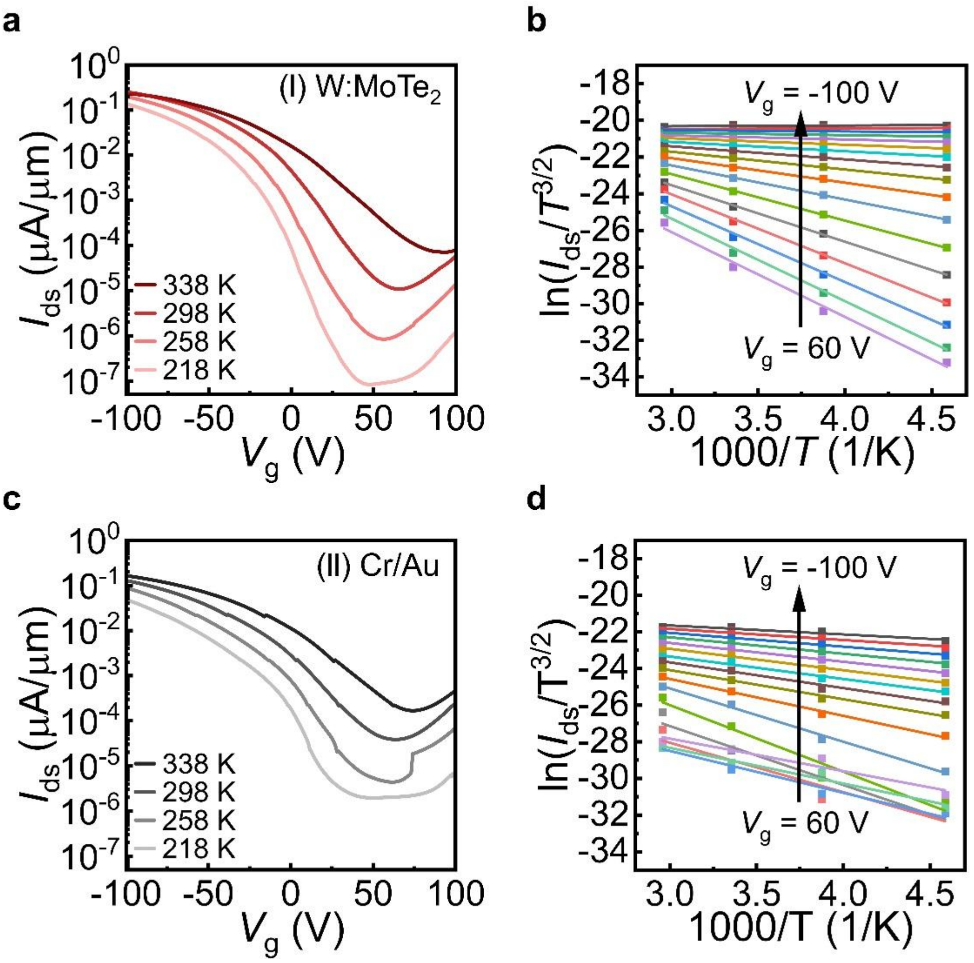

Figure S33. Schottky barrier heights of FETs with W:$MoTe_2$ edge contact and Cr/Au top contact. (**a**) Transfer curves of W:$MoTe_2$ edge-contact FET at V_{ds} = -1 V, measured at different T (218-338 K). (**b**) Arrhenius plot (ln($I_{ds}/T^{3/2}$) vs. 1000/T) of the corresponding edge-contact FET at various V_g from 60 to -100 V. (**c**) T-dependent transfer curves of Cr/Au top-contact FET at V_{ds} = -1 V. (**d**) Arrhenius plot of top-contact FET.

Note that, distinct from the Cr/Au top contact, a nearly-zero slope is observed in the Arrhenius plot for the W:$MoTe_2$ edge-contact FET, when the high negative voltage is applied ($V_g < V_{FB} \approx$ -30 V) (**Figures S33b,d** and **5f**). This indicates that the thermionic barrier height for the edge-contact FET is negligible, suggesting that charge injection primarily relies on tunneling transport through the MSJ in the sub-threshold regime.

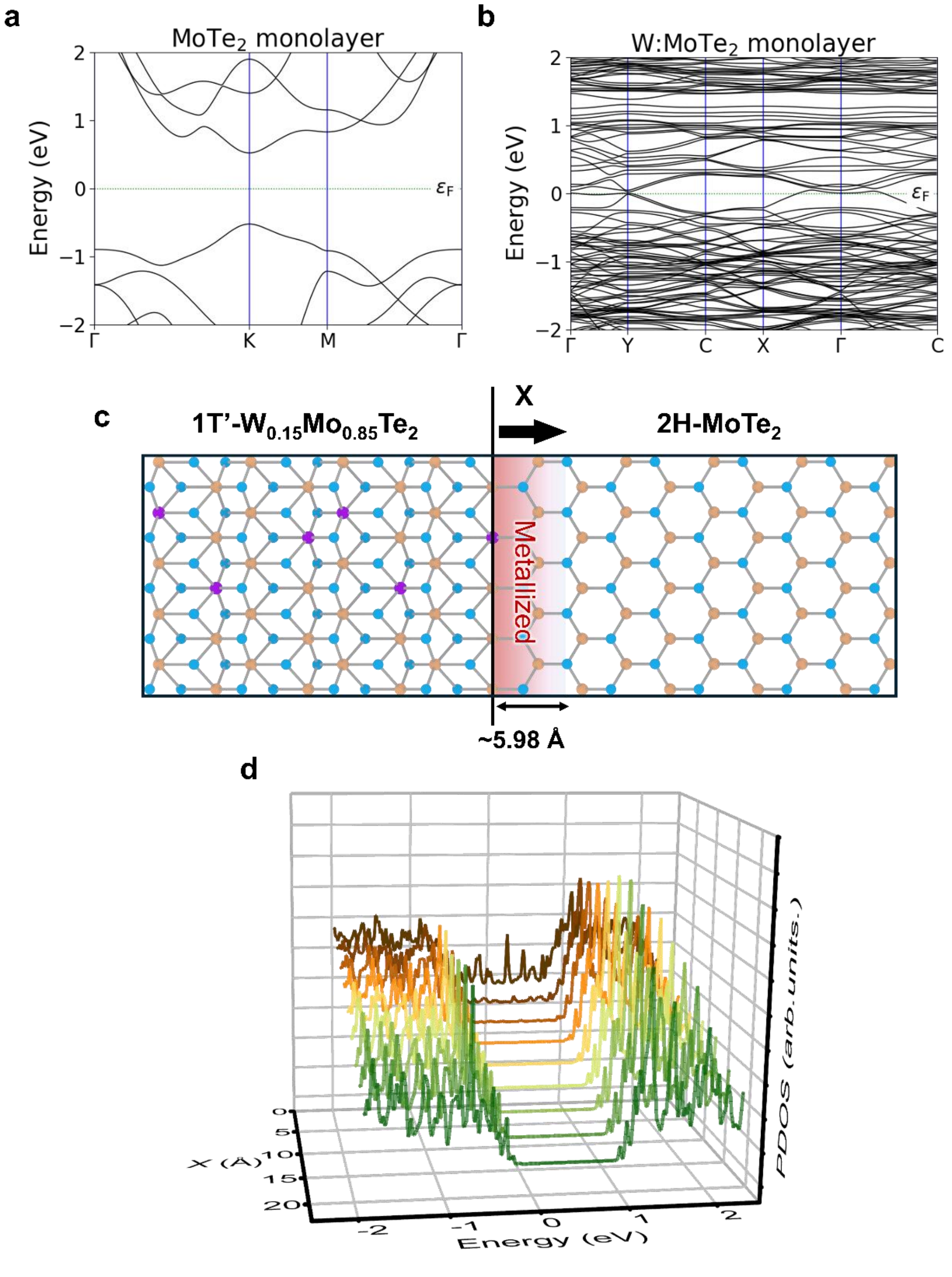

Figure S34. DFT calculations of in-plane $MoTe_2$-W:$MoTe_2$ heterostructure. (**a**,**b**) Band structures of (**a**) monolayer 2H-$MoTe_2$ and (**b**) $W_{0.15}Mo_{0.85}Te_2$. (**c**) Schematic of the $W_{0.15}Mo_{0.85}Te_2$-$MoTe_2$ lateral heterostructure. The region with red color gradient illustrates the decay of the MIGS from the contact interface. (**d**) Projected DOS as a function of energy at positions starting from the W:$MoTe_2$-$MoTe_2$ interface. The adjacent 2H-$MoTe_2$ region near the interface (x~5.98 Å) is metallized through strong hybridization, as illustrated in (c).

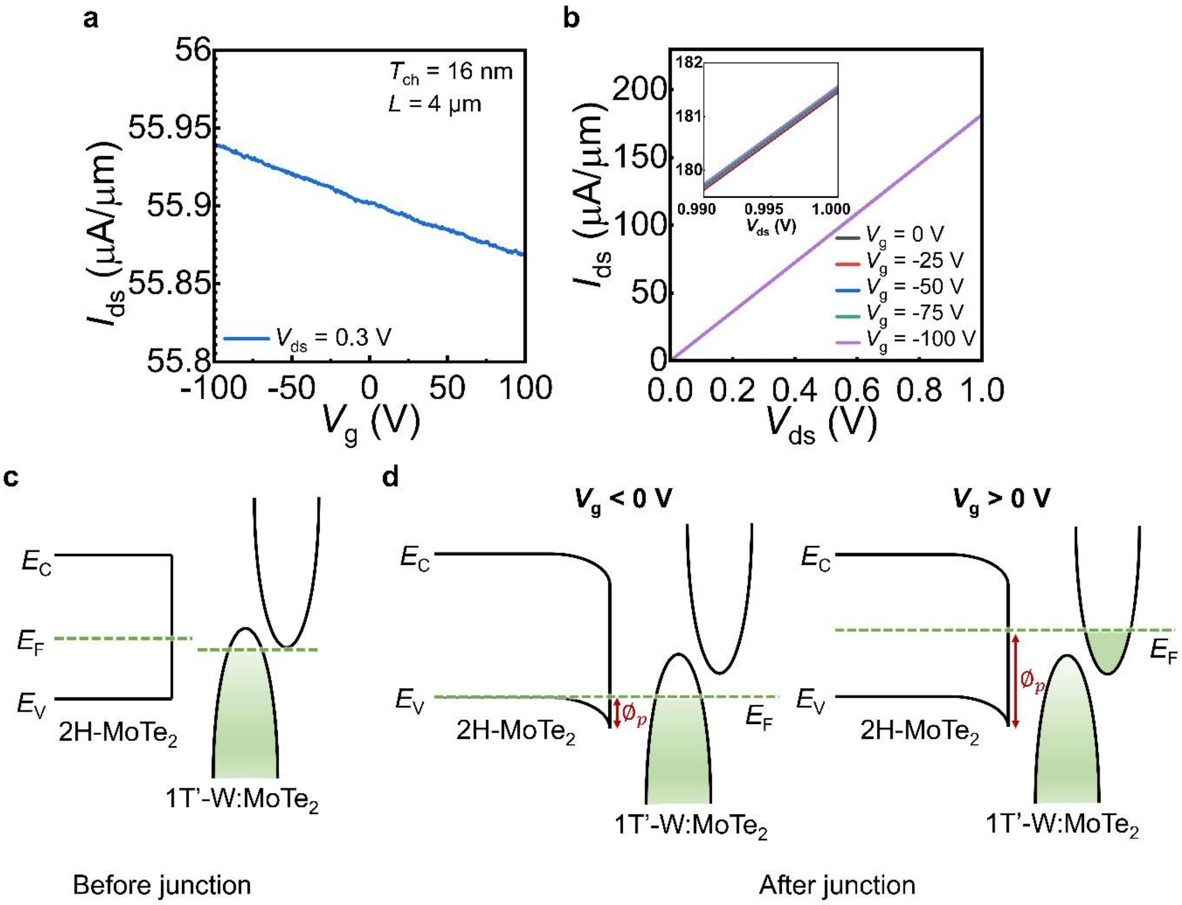

Figure S35. Conductivity modulation of semimetal 1T'-W:MoTe$_2$ as applying V_g. (**a,b**) Transfer and output curves of semimetal 1T'-$W_{0.17}Mo_{0.83}Te_2$ with T_{ch} = 16 nm and L = 4 μm. (**a**) Transfer curve at V_{ds} = 0.3 V. (**b**) Output curves with different V_g steps from 0 to -100 V. The plots depict the major carrier in W:MoTe$_2$ is the hole as the increasing current at negative V_g. The inset shows the I_{ds}-V_{ds} curves over a narrow range, illustrating a slight increase in I_{ds} as V_g is varied from 0 to -100 V. (**c,d**) Band diagrams depicting the formation of thermionic barrier height (Φ_p) at the edge-contact MoTe$_2$-W:MoTe$_2$ heterostructure. (**c**) The band diagram of MoTe$_2$ and W:MoTe$_2$ before the junction is created. The dashed lines indicate the Fermi level of each material. According to literature,[12] the work function (WF) for the 2H- and 1T'-phase MoTe$_2$ are ≈4.44 and ≈4.51 eV, respectively. (**d**) The band diagram of MoTe$_2$ junction with edge-contact W:MoTe$_2$ under the different case of applying V_g. Owing to the small DOS in the semimetal 1T'-W:MoTe$_2$, the work function of the W:MoTe$_2$ can be tuned by the applied V_g to accommodate the accumulated charge. Consequently, the Schottky barrier height (Φ_p) can be adjusted through gate electrostatics.[35,36]

Table S1. Summary of characterization results demonstrating the single crystallinity of 2H-$MoTe_2$ in the channel region.

Characterization method	Observation	Evidence of single crystallinity	Identifiable single-crystal size	Figures
BF-STEM image	Absence of grain boundaries (bright lines)	Absence of grain boundaries	~1 μm^2	Fig. 3a
SAED pattern	Single set of diffraction spots	Direct evidence of the single-crystalline nature	~1 μm^2	Fig. 3b and Fig. S16a
SAED patterns collected at multiple locations	Single set of diffraction spots, negligible misalignment	Direct evidence of single-crystalline over a large area	~100 μm^2	Figs. S4e-g
EBSD	Negligible misalignment of Kikuchi patterns in each single-channel region	Direct evidence of single-crystalline over a large area	~1500 μm^2	Figs. S4a-d
EBSD	Uniform inverse pole figure (IPF) map without misorientation	Absence of grain boundaries, confirming a single crystal region	~1 mm^2	Fig. S5

Table S2. Benchmark table of different metallization processes for 2H-$MoTe_2$ FET.

	**Process simplicity;** total number of steps	**Contact length scaling (nm)**	**Channel quality;** Raman E_{2g} FWHM change	**On/off ratio**	**Mobility variation, C_V (%)**
**(I) Edge contact** (W:$MoTe_2$)	4	8	1	≈7.96 × 10^4	7.2
**(II) Edge contact** (T_d-WTe_2 or 1T'-$MoTe_2$	7	8	1.03	≈2.58 × 10^3	61.6
**(II) Top contact** (Cr/Au)	4	252	1.05	≈1.81 × 10^3	28.6

Process simplicity. In **Figure 4F and Table S2**, "process simplicity" indicates the number of process steps required to produce the FETs. Compared with the one-step process, two-step edge contact fabrication (T_d-WTe_2, 1T'-$MoTe_2$) necessitates an additional area-selective growth, accompanied by a second photolithography, RIE, and DC sputtering for W (or Mo) precursor (**Figure S19**). For Cr/Au top contact, the total number of fabrication steps is the same as for W:$MoTe_2$ edge contact.

Contact length scaling. According to Ref.,[1] physical contact length (L_c) of edge contacts has little influence on charge transfer compared with transfer length (L_T), which makes edge contacts immunity to contact-length scaling. Specifically, in top-contact FETs, L_T is defined as $L_T \approx (\rho_c/R_{sh})^{1/2}$ (Ref.[1,37,38]) and contact resistance (R_c) depends on L_c. According to the transmission line model, R_c increases significantly when $L_c \leq L_T$, as described by the transmission line model, $R_c = (\rho_c R_{sh})^{1/2}\coth(L_c/L_T)$.[39] In contrast, in edge-contact FETs, R_c depends solely on L_T and is not influenced by the physical contact length, making it feasible to reduce L_c even below L_T[1,37-39] (**Figures 5a,b** and **Experimental section**). Therefore, the contact length scaling is limited by its L_T, which is ~252 nm for 2H-$MoTe_2$ FET. In contrast, the contact length for the edge-contact FET can be scaled below the L_T, which corresponds to thickness of the channel. The L_T value for top-contact FET falls within the range of previously reported values of $MoTe_2$ (115 nm and 374 nm for Ag and Pd/Au top-contact $MoTe_2$ FETs).[29,40]

Channel quality. The "channel quality" depending on metallization processes was investigated via Raman characterizations for E_{2g} vibration modes of 2H-$MoTe_2$ (**Figure S21)**.

On/off ratio. Described on/off ratios of $MoTe_2$ FETs with various contact types were extracted from **Figure 4k**. For the (II) edge-contact devices, the presented value represents the average values of 1T'-WTe_2 ($\approx 4.16 \times 10^3$) and 1T'-$MoTe_2$ ($\approx 1.01 \times 10^3$) are presented.

Mobility variation. The "mobility variation" was represented by the coefficient variation (C_V) (**Figures 4f,n**).

Table S3. Benchmarking table of *p*-type edge-contact FETs with various TMD channel materials. This table summarizes *p*-type FETs with pure edge contacts, in terms of channel growth method, contact metal, process simplicity, scalability, R_c extracted using TLM, mobility variation expressed as the coefficient of variation (C_V = standard deviation/average), μ_{FE}, I_{on}/I_{off}, and on-state total sheet conductance ($G_{on,tot} = (I_{on}/V_{ds})(L/W)$) to allow fair comparison among devices with different dimensions. For process simplicity, we define "one-step" as the simultaneous formation of the metal and semiconductor, and "two-step" as metallization conducted after channel formation. Scalability refers to the size of transistor integration reported in each study. "No" in scalability indicates that only one transistor was fabricated, which is typically the case for device based on mechanical exfoliation (ME) flakes. To ensure a fair comparison of uniformity, mobility variation is presented as C_V values calculated from multiple devices. "N/A" in the table indicates that the corresponding data were not available in the papers.

Channel material	Channel growth method	Contact metal	Process simplicity	Scalability (chip size)	TLM -R_c (kΩ·µm)	Mobility variation; C_V	μ_{FE} ($cm^2V^{-1}s^{-1}$)	I_{on}/I_{off}	$G_{on,tot}$ (µS)	Ref
**$MoTe_2$**	**CVD**	**W:$MoTe_2$**	**One-step**	**2-inch wafer (>5 cm^2)**	**7.5**	**0.072 (13 devices), 0.18 (59 devices)**	**11.3**	**8×10^4**	**6.7**	**This work**
$MoTe_2$	CVD	1T'-$MoTe_2$	One-step	No	14	N/A	16.2	10^4	N/A	[7]
$MoTe_2$	CVD	1T'-$MoTe_2$	One-step	>1 mm^2	7.1	0.19	5.6	10^2	5.2	[41]
$MoTe_2$	CVD	1T'-$MoTe_2$	One-step	No	N/A	N/A	7-8	3.5×10^3	6.8	[8]
WSe_2	ME	Cr, Pd, In	Two-step	No	N/A	N/A	N/A	10^5	0.006-0.018	[42]
WS_2	ME	Pt	Two-step	No	N/A	N/A	N/A	10^7	4.5	[43]
$PtSe_2$	MBE	$PtTe_2$	Two-step	No	N/A	N/A	0.216	2.5×10^2	0.14	[44]
$PtSe_2$	CVD	$PtTe_2$	Two-step	>1 cm^2	13.8	N/A	50	2.5×10^2	2.86	[45]

Table S4. Electrical characteristics of WSe_2 FETs with various metal contacts, compared with the W:$MoTe_2$-$MoTe_2$ edge-contact FET presented in this work. "ME" denotes mechanical exfoliation. $G_{on,tot}$ represents the on-state total sheet conductance, defined as $G_{on,tot} = (I_{on}/V_{ds})(L/W)$. "N/A" in the table indicates that the corresponding data were not available in the referenced papers.

Channel growth	Thickness	Contact metal	$G_{on,tot}$ (μS)	Gate	$\|V_{ds}\|$ (V)	Polarity type	Ref
**$MoTe_2$ (CVD)**	**8 nm**	**W:$MoTe_2$**	**6.7**	**Global back gate (SiO_2)**	**1**	**p-type unipolar**	**This work**
WSe_2 (MOCVD)	bilayer	Pd	0.44	Global back gate (Al_2O_3)	1	p-type unipolar	[46]
WSe_2 (MOCVD)	monolayer	Pd/Au	0.1	Global back gate (Al_2O_3)	1	p-type unipolar	[47]
WSe_2 (CVD)	monolayer	Au	7	Global back gate (SiO_2)	1	p-type unipolar	[48]
WSe_2 (CVD)	monolayer	N/A	0.2	Global back gate (SiO_2)	1	p-type unipolar	[49]
WSe_2 (CVD)	monolayer	Sb/Pt	2.5	Global back gate (SiN_x)	1	p-type dominant ambipolar	[50]
WSe_2 (ME)	3 nm	$PdTe_2$	2.6	Global back gate (SiO_2)	1	p-type dominant ambipolar	[51]
WSe_2 (ME)	8-12 nm	Cl-$SnSe_2$	6	Local bottom gate (hBN)	1	p-type unipolar	[52]
WSe_2 (ME)	12 nm	Pt (Se buffer layer)	10	Global back gate (SiO_2/hBN)	1	p-type unipolar	[53]
WSe_2 (ME)	7 nm	Au (transfer)	3.06	Global back gate (SiO_2)	1	p-type unipolar	[54]
WSe_2 (ME)	5 nm	Pd (transfer)	10	Global back gate (SiO_2)	1	p-type unipolar	[55]

References

[1] Cheng Z, Yu Y, Singh S, et al. Immunity to contact scaling in MoS_2 transistors using in situ edge contacts. *Nano Lett*. 2019, 19, 5077.

[2] Conde-Rubio A, Liu X, Boero G, Brugger J. Edge-Contact MoS_2 Transistors Fabricated Using Thermal Scanning Probe Lithography. *ACS Appl. Mater. Interfaces* 2022, 14, 42328.

[3] Cho S, Kim S, Kim JH, et al. Phase patterning for ohmic homojunction contact in $MoTe_2$. *Science* 2015, 349, 625.

[4] Ryu H, Lee Y, Jeong JH, et al. Laser-Induced Phase Transition and Patterning of hBN-Encapsulated $MoTe_2$. *Small* 2023, 19, 2205224.

[5] Xu X, Liu S, Han B, et al. Scaling-up atomically thin coplanar semiconductor–metal circuitry via phase engineered chemical assembly. *Nano Lett*. 2019, 19, 6845.

[6] Xu X, Pan Y, Liu S, et al. Seeded 2D epitaxy of large-area single-crystal films of the van der Waals semiconductor 2H $MoTe_2$. *Science* 2021, 372, 195.

[7] Sung JH, Heo H, Si S, et al. Coplanar semiconductor–metal circuitry defined on few-layer $MoTe_2$ via polymorphic heteroepitaxy. *Nat. Nanotechnol*. 2017, 12, 1064.

[8] Ma R, Zhang H, Yoo Y, et al. $MoTe_2$ lateral homojunction field-effect transistors fabricated using flux-controlled phase engineering. *ACS Nano* 2019, 13, 8035.

[9] Zhang X, Jin Z, Wang L, et al. Low contact barrier in 2H/1T′ $MoTe_2$ in-plane heterostructure synthesized by chemical vapor deposition. *ACS Appl. Mater. Interfaces* 2019, 11, 12777.

[10] Zhang Q, Wang X-F, Shen S-H, et al. Simultaneous synthesis and integration of two-dimensional electronic components. *Nat. Electron*. 2019, 2, 164.

[11] Xu X, Chen S, Liu S, et al. Millimeter-scale single-crystalline semiconducting $MoTe_2$ via solid-to-solid phase transformation. *J. Am. Chem. Soc*. 2019, 141, 2128.

[12] Song S, Yoon A, Jang S, et al. Fabrication of p-type 2D single-crystalline transistor arrays with Fermi-level-tuned van der Waals semimetal electrodes. *Nat. Commun*. 2023, 14, 4747.

[13] Xia Y, Chen X, Wei J, et al. 12-inch growth of uniform MoS_2 monolayer for integrated circuit manufacture. *Nat. Mater*. 2023, 22, 1324.

[14] Sangwan VK, Jariwala D, Kim IS, et al. Gate-tunable memristive phenomena mediated by grain boundaries in single-layer MoS_2. *Nat. Nanotechnol*. 2015, 10, 403.

[15] Keller RR, Geiss RH. Transmission EBSD from 10 nm domains in a scanning electron microscope. *J. Microsc*. 2012, 245, 245.

[16] Andrews CE, Strantza M, Calta NP, Matthews MJ, Taheri ML. A Denoising Autoencoder for Improved Kikuchi Pattern Quality and Indexing in Electron Backscatter Diffraction. *Ultramicroscopy* 2023, 253, 113810.

[17] Oliver SM, Beams R, Krylyuk S, et al. The structural phases and vibrational properties of $Mo_{1-x}W_xTe_2$ alloys. *2D Mater*. 2017, 4, 045008.

[18] Kwak J, Jo Y, Song S, et al. Single-crystalline nanobelts composed of transition metal ditellurides. *Adv. Mater*. 2018, 30, 1707260.

[19] Zhang M, Wu J, Zhu Y, et al. Two-dimensional molybdenum tungsten diselenide alloys: photoluminescence, Raman scattering, and electrical transport. *ACS Nano* 2014, 8, 7130.

[20] Aslan B, Datye IM, Mleczko MJ, et al. Probing the optical properties and strain-tuning of ultrathin $Mo_{1-x}W_xTe_2$. *Nano Lett*. 2018, 18, 2485.

[21] Huang L-F, Zeng Z. Roles of mass, structure, and bond strength in the phonon properties and lattice anharmonicity of single-layer Mo and W dichalcogenides. *J. Phys. Chem. C* 2015, 119, 18779.

[22] Cheng Z, Jia X, Cheng X, et al. Large-Scale N-Type FET and Homogeneous CMOS Inverter Array Based on Few-Layer $MoTe_2$. *Adv. Electron. Mater*. 2023, 9, 2300268.

[23] Song S, Sim Y, Kim S-Y, et al. Wafer-scale production of patterned transition metal ditelluride layers for two-dimensional metal–semiconductor contacts at the Schottky–Mott limit. *Nat. Electron*. 2020, 3, 207.

[24] Keum DH, Cho S, Kim JH, et al. Bandgap opening in few-layered monoclinic $MoTe_2$. *Nat. Phys*. 2015, 11, 482.

[25] Longo RC, Addou R, Santosh K, et al. Intrinsic air stability mechanisms of two-dimensional transition metal dichalcogenide surfaces: basal versus edge oxidation. *2D Mater*. 2017, 4, 025050.

[26] Jain A, Szabó Á, Parzefall M, et al. One-dimensional edge contacts to a monolayer semiconductor. *Nano Lett*. 2019, 19, 6914.

[27] Lin C-P, Chen P-C, Huang J-H, et al. Local modulation of electrical transport in 2D layered materials induced by electron beam irradiation. *ACS Appl. Electron. Mater*. 2019, 1, 684.

[28] Liu Y, Guo J, Zhu E, et al. Approaching the Schottky–Mott limit in van der Waals metal–semiconductor junctions. *Nature* 2018, 557, 696.

[29] Mleczko MJ, Yu AC, Smyth CM, et al. Contact engineering high-performance n-type $MoTe_2$ transistors. *Nano Lett*. 2019, 19, 6352.

[30] Liu F, Wang L, Wang J, et al. 2D Ruddlesden–Popper perovskite single crystal field-effect transistors. *Adv. Funct. Mater*. 2021, 31, 2005662.

[31] Wang Y, Chhowalla M. Making clean electrical contacts on 2D transition metal dichalcogenides. *Nat. Rev. Phys*. 2022, 4, 101.

[32] Song S, Yoon A, Ha J-K, et al. Atomic transistors based on seamless lateral metal-semiconductor junctions with a sub-1-nm transfer length. *Nat. Commun*. 2022, 13, 4916.

[33] Shen P-C, Su C, Lin Y, et al. Ultralow contact resistance between semimetal and monolayer semiconductors. *Nature* 2021, 593, 211.

[34] Wu W-C, Hung TY, Sathaiya DM, et al. Comprehensive Study of Contact Length Scaling Down to 12 nm With Monolayer MoS_2 Channel Transistors. *IEEE Trans. Electron Devices* 2023, 70, 6680.

[35] Parto K, Pal A, Chavan T, et al. One-Dimensional Edge Contacts to Two-Dimensional Transition-Metal Dichalcogenides: Uncovering the Role of Schottky-Barrier Anisotropy in Charge Transport across MoS_2/Metal Interfaces. *Phys. Rev. Appl.* 2021, 15, 064068.

[36] Li X, Wei Y, Wang Z, et al. One-dimensional semimetal contacts to two-dimensional semiconductors. *Nat. Commun*. 2023, 14, 111.

[37] Lee S, Choi H, Moon I, et al. Contact resistivity in edge-contacted graphene field effect transistors. *Adv. Electron. Mater*. 2022, 8, 2101169.

[38] Pal A, Mishra V, Weber J, et al. Characterization and closed-form modeling of edge/top/hybrid metal-2D semiconductor contacts. IEEE, 2022, 28.5. 1.

[39] Allain A, Kang J, Banerjee K, Kis A. Electrical contacts to two-dimensional semiconductors. *Nat. Mater*. 2015, 14, 1195.

[40] Cong X, Shah MNU, Zheng Y, He W. Largely Reducing the Contact Resistance of Molybdenum Ditelluride by In Situ Potassium Modification. *Adv. Electron. Mater*. 2023, 9, 2300062.

[41] Zhang S, Wu Y, Gao F, et al. Field effect transistor sensors based on in-plane 1T′/2H/1T′ $MoTe_2$ heterophases with superior sensitivity and output signals. *Adv. Funct. Mater*. 2022, 32, 2205299.

[42] Ngo TD, Choi MS, Lee M, Ali F, Yoo WJ. Anomalously persistent p-type behavior of WSe_2 field-effect transistors by oxidized edge-induced Fermi-level pinning. *J. Mater. Chem. C* 2022, 10, 846.

[43] Ko S, Lee D, Kim J, Kim C-K, Kim J. Self-Aligned Edge Contact Process for Fabricating High-Performance Transition-Metal Dichalcogenide Field-Effect Transistors. *ACS Nano* 2024, 18, 25009.

[44] Kim H-S, Jeong J, Kwon G-H, Kwon H, Baik M, Cho M-H. Improvement of electrical performance using $PtSe_2$/$PtTe_2$ edge contact synthesized by molecular beam epitaxy. *Appl. Surf. Sci*. 2022, 585, 152507.

[45] Han SS, Sattar S, Kireev D, et al. Reversible transition of semiconducting $PtSe_2$ and metallic $PtTe_2$ for scalable all-2D edge-contacted FETs. *Nano Lett*. 2023, 24, 1891.

[46] Oberoi A, Han Y, Stepanoff SP, et al. Toward high-performance p-type two-dimensional field effect transistors: contact engineering, scaling, and doping. *ACS Nano* 2023, 17, 19709.

[47] Zhu H, Nayir N, Choudhury TH, et al. Step engineering for nucleation and domain orientation control in WSe_2 epitaxy on c-plane sapphire. *Nat. Nanotechnol* 2023, 18, 1295.

[48] Zhou H, Wang C, Shaw JC, et al. Large area growth and electrical properties of p-type WSe_2 atomic layers. *Nano Lett*. 2015, 15, 709.

[49] Moon D, Lee W, Lim C, et al. Hypotaxy of wafer-scale single-crystal transition metal dichalcogenides. *Nature* 2025, 638, 957.

[50] Chou A-S, Lin Y-T, Lin YC, et al. High-performance monolayer WSe_2 p/n FETs via antimony-platinum modulated contact technology towards 2D CMOS electronics. IEEE, 2022,

7.2. 1.

[51] Zheng J, Miao T, Xu R, et al. Chemical Synthesis and Integration of Highly Conductive $PdTe_2$ with Low-Dimensional Semiconductors for p-Type Transistors with Low Contact Barriers. *Adv. Mater*. 2021, 33, 2101150.

[52] Jang J, Ra HS, Ahn J, et al. Fermi-Level Pinning-Free WSe_2 Transistors via 2D Van der Waals Metal Contacts and Their Circuits. *Adv. Mater*. 2022, 34, 2109899.

[53] Kwon G, Choi Y-H, Lee H, et al. Interaction-and defect-free van der Waals contacts between metals and two-dimensional semiconductors. *Nat. Electron*. 2022, 5, 241.

[54] Kong L, Zhang X, Tao Q, et al. Doping-free complementary WSe_2 circuit via van der Waals metal integration. *Nat. Commun*. 2020, 11, 1866.

[55] Wu H, Yan Z, Xie Z, Zhu S. WSe_2/Pd Schottky diode combining van der Waals integrated and evaporated metal contacts. *Appl. Phys. Lett*. 2021, 119, 213102.
